# Supplementary material for: Does public transport use prevent declines in walking speed among older adults living in England? A prospective cohort study
Source: BMJ Open. 2017 Sep 28;7(9):e017702. doi: 10.1136/bmjopen-2017-017702 (PMC5652510; doi:10.1136/bmjopen-2017-017702)
Supplement: Supplementary data [file bmjopen-2017-017702supp001.pdf]

**Does public transport use prevent declines in walking speed among older adults living in England? A prospective cohort study**

**Patrick Rouxel, PhD[1], Elizabeth Webb, PhD [2] and Tarani Chandola PhD [3]**

[1]CLOSER, Department of Social Science, University College London Institute of Education, London, UK, ORCID: 0000-0003-0330-554X, [2] International Centre for Lifecourse Studies, Department of Epidemiology and Public Health, University College London, London, UK, ORCID: 0000-0001-6106-5367, and [3]CMIST and Social Statistics, University of Manchester, UK, ORCID: 0000-0002-1864-3413

**To BMJ Open**

**Corresponding author**

Patrick Rouxel, PhD, CLOSER, Department of Social Science, University College London Institute of Education, 55-59 Gordon Square, WC1H 0NU, London, UK. E-mail: patrick.rouxel@ucl.ac.uk

Supplementary Table S1 Log odds (95% CI) from logit model of any missing data in the walking speed analyses, conditional on having a baseline walking speed measurement: ELSA waves 2-6

| | **Log odds** | **Lower 95% CI** | **Upper 95% CI** |
|---|---|---|---|
| *Intercept* | **0.54** | -0.17 | 1.26 |
| *Gender (ref: Male)* | **-0.22** | -0.31 | -0.13 |
| *Age centered (linear term)* | **-0.02** | -0.03 | -0.01 |
| *Gender*Age (ref: Male)* | **-0.01** | -0.02 | -0.004 |
| *Age (quadratic term)* | **0.01** | 0.01 | 0.01 |
| *Frequency of public transport use(ref: every day or nearly every day )* | | | |
| two or three times a week | -0.07 | -0.24 | 0.09 |
| once a week | **-0.32** | -0.48 | -0.16 |
| did not use because no need | 0.02 | -0.12 | 0.17 |
| did not use because health problems | **0.69** | 0.43 | 0.95 |
| did not use because structural reasons | 0.04 | -0.11 | 0.19 |
| *Access to car/van (ref: yes access)* | | | |
| No access to car/van | **0.25** | 0.11 | 0.38 |
| *Employment status (ref: employed)* | | | |
| Retired | **0.14** | 0.02 | 0.25 |
| Other | 0.03 | -0.10 | 0.15 |
| *Social class (ref: Managerial & Prof)* | | | |
| Intermediate occupations | 0.03 | -0.09 | 0.16 |
| Self-employed | **0.14** | 0.01 | 0.27 |
| Lower supervisory & technical | 0.05 | -0.09 | 0.18 |
| Semi-routine & routine | **0.21** | 0.11 | 0.31 |
| *Urban/Rural (ref: Urban)* | | | |
| Town & Fringe | 0.02 | -0.10 | 0.13 |
| Village | -0.13 | -0.25 | 0.002 |
| Hamlet/Isolated | 0.08 | -0.12 | 0.27 |
| *Mobility difficulties (ref: none)* | | | |
| 1-3 difficulties | -0.05 | -0.14 | 0.04 |
| 4+ difficulties | **0.15** | 0.01 | 0.29 |
| *Activities of Daily living impairments (ref: none)* | | | |
| At least one ADL impairment | **0.16** | 0.03 | 0.28 |
| *Physical Activity levels (ref: sedentary)* | | | |
| Low | **-0.80** | -1.04 | -0.55 |
| Moderate | **-0.98** | -1.22 | -0.74 |
| High | **-1.00** | -1.25 | -0.74 |
| *Marital status (ref: married)* | | | |
| Separated/Divorced | **0.43** | 0.25 | 0.61 |
| Widowed | **0.45** | 0.24 | 0.65 |
| Never married | **0.66** | 0.43 | 0.89 |
| *Cohabitation (ref: not living with partner)* | | | |
| Living with partner | **0.69** | 0.51 | 0.88 |
| *CESD- Depression score* | **0.04** | 0.01 | 0.06 |

Supplementary Table S1 continued Log odds (95% CI) from logit model of any missing data in the walking speed analyses, conditional on having a baseline walking speed measurement: ELSA waves 2-6

| | **Log odds** | **Lower 95% CI** | **Upper 95% CI** |
|---|---|---|---|
| *Smoking status (ref: never smoker)* | | | |
| Ex-Smoker | -0.02 | -0.10 | 0.06 |
| Current smoker | **0.25** | 0.13 | 0.36 |
| *Date/Day orientation (ref: no correct answer)* | | | |
| 1 correct answer | 0.005 | -0.93 | 0.94 |
| 2 correct answers | 0.04 | -0.67 | 0.75 |
| 3 correct answers | -0.47 | -1.10 | 0.16 |
| All correct answers | -0.52 | -1.14 | 0.11 |
| *Memory test* | **-0.06** | -0.07 | -0.04 |

**Boldface** indicates statistical significance ($p<0.05$)

Supplementary Table S2 Coefficients (95% CI) from the Fixed Effects Model of walking speed (m/s), ELSA waves 2-6

| | **Coefficients** | **Lower 95% CI** | **Upper 95% CI** |
|---|---|---|---|
| *Intercept* | **0.82** | 0.76 | 0.88 |
| *Age centered (linear term)* | **-0.01** | -0.01 | -0.01 |
| *Age (quadratic term)* | **-0.0003** | -0.0004 | -0.0003 |
| *Frequency of public transport use(ref: every day or nearly every day )* | | | |
| two or three times a week | -0.01 | -0.02 | 0.0001 |
| once a week | **-0.02** | -0.03 | -0.01 |
| did not use because no need | **-0.02** | -0.03 | -0.003 |
| did not use because health problems | **-0.06** | -0.08 | -0.04 |
| did not use because structural reasons | **-0.02** | -0.03 | -0.01 |
| *Wealth quintiles (ref: poorest quintile)* | | | |
| Quintile 2 | 0.01 | -0.01 | 0.02 |
| Quintile 3 | 0.01 | -0.01 | 0.03 |
| Quintile 4 | 0.02 | -0.01 | 0.04 |
| Richest quintile | **0.03** | 0.003 | 0.05 |
| *Access to car/van (ref: yes access)* | | | |
| No access to car/van | -0.01 | -0.02 | 0.0005 |
| *Employment status (ref: employed)* | | | |
| Retired | 0.00 | -0.01 | 0.01 |
| Other | 0.00 | -0.01 | 0.02 |
| *Social class (ref: Managerial & Prof)* | | | |
| Intermediate occupations | -0.02 | -0.06 | 0.02 |
| Self-employed | 0.01 | -0.03 | 0.05 |
| Lower supervisory & technical | -0.02 | -0.06 | 0.03 |
| Semi-routine & routine | 0.02 | -0.02 | 0.06 |
| *Urban/Rural (ref: Urban)* | | | |
| Town & Fringe | 0.02 | -0.03 | 0.06 |
| Village | 0.01 | -0.03 | 0.05 |
| Hamlet/Isolated | 0.03 | -0.03 | 0.08 |
| *Mobility difficulties (ref: none)* | | | |
| 1-3 difficulties | **-0.01** | -0.02 | -0.01 |
| 4+ difficulties | **-0.05** | -0.06 | -0.04 |
| *Activities of Daily living impairments (ref: none)* | | | |
| At least one ADL impairment | **-0.03** | -0.04 | -0.02 |
| *Physical Activity levels (ref: sedentary)* | | | |
| Low | **0.03** | 0.02 | 0.04 |
| Moderate | **0.05** | 0.04 | 0.07 |
| High | **0.06** | 0.05 | 0.08 |
| *Marital status (ref: married)* | | | |
| Separated/Divorced | **0.04** | 0.01 | 0.07 |
| Widowed | 0.01 | -0.02 | 0.04 |
| Never married | 0.02 | -0.04 | 0.07 |
| *Cohabitation (ref: not living with partner)* | | | |
| Living with partner | 0.01 | -0.02 | 0.05 |
| *CESD- Depression score* | -0.01 | -0.01 | -0.01 |

Supplementary Table S2 continued Coefficients (95% CI) from the Fixed Effects Model of walking speed (m/s), ELSA waves 2-6

| *Smoking status (ref: never smoker)* | **Coefficients** | **Lower 95% CI** | **Upper 95% CI** |
|---|---|---|---|
| Ex-Smoker | 0.00 | -0.02 | 0.03 |
| Current smoker | 0.01 | -0.02 | 0.04 |
| *Date/Day orientation (ref: no correct answer)* | | | |
| 1 correct answer | 0.02 | -0.03 | 0.07 |
| 2 correct answers | 0.04 | -0.01 | 0.08 |
| 3 correct answers | **0.05** | 0.01 | 0.09 |
| All correct answers | **0.05** | 0.01 | 0.09 |
| *Memory test* | **0.001** | 0.00002 | 0.002 |
| Number of observations | 27509 | | |
| Adjusted R-squared | 0.73 | | |

**Boldface** indicates statistical significance (p<0.05)

Supplementary Table S3 Coefficients (95% CI) from the Multilevel Growth Curve Model of walking speed (m/s), ELSA waves 2-6

| **Fixed Part** | **Coefficients** | **Lower 95% CI** | **Upper 95% CI** |
|---|---|---|---|
| *Intercept* | **0.83** | 0.79 | 0.87 |
| *Age centered (linear term)* | **-0.01** | -0.01 | -0.004 |
| *Age (quadratic term)* | **-0.0002** | -0.0002 | -0.0002 |
| *Frequency of public transport use(ref: every day or nearly every day )* | | | |
| two or three times a week | -0.01 | -0.02 | 0.01 |
| once a week | -0.01 | -0.02 | 0.01 |
| did not use because no need | -0.005 | -0.02 | 0.01 |
| did not use because health problems | **-0.12** | -0.14 | -0.09 |
| did not use because structural reasons | -0.002 | -0.014 | 0.011 |
| *Interaction between Age (linear term) and Frequency of public transport use (ref: nearly/every day use)* | | | |
| Age*two or three times a week | -0.0003 | -0.0017 | 0.0011 |
| Age*once a week | -0.0010 | -0.0024 | 0.0004 |
| Age*did not use because no need | **-0.0012** | -0.0024 | -0.00002 |
| Age*did not use because health problems | **0.0017** | 0.0001 | 0.0033 |
| Age*did not use because structural reasons | **-0.0014** | -0.0026 | -0.0002 |
| *Sex (ref: men)* | | | |
| Women | **-0.02** | -0.03 | -0.02 |
| *Wealth quintiles (ref: poorest quintile)* | | | |
| Quintile 2 | **0.02** | 0.01 | 0.03 |
| Quintile 3 | **0.04** | 0.03 | 0.05 |
| Quintile 4 | **0.06** | 0.05 | 0.07 |
| Richest quintile | **0.09** | 0.08 | 0.10 |
| *Access to car/van (ref: yes access)* | | | |
| No access to car/van | **-0.02** | -0.03 | -0.02 |
| *Employment status (ref: employed)* | | | |
| Retired | -0.01 | -0.02 | 0.00002 |
| Other | **-0.02** | -0.03 | -0.01 |
| *Social class (ref: Managerial & Prof)* | | | |
| Intermediate occupations | **-0.02** | -0.03 | -0.01 |
| Self-employed | **-0.02** | -0.03 | -0.01 |
| Lower supervisory & technical | **-0.05** | -0.06 | -0.04 |
| Semi-routine & routine | **-0.04** | -0.05 | -0.03 |
| *Urban/Rural (ref: Urban)* | | | |
| Town & Fringe | **0.01** | 0.003 | 0.02 |
| Village | **0.02** | 0.01 | 0.03 |
| Hamlet/Isolated | **0.02** | 0.00 | 0.04 |
| *Mobility difficulties (ref: none)* | | | |
| 1-3 difficulties | **-0.04** | -0.04 | -0.03 |
| 4+ difficulties | **-0.12** | -0.13 | -0.11 |
| *Activities of Daily living impairments (ref: none)* | | | |
| At least one ADL impairment | **-0.06** | -0.06 | -0.05 |

Supplementary Table S3 continued Coefficients (95% CI) from the Multilevel Growth Curve Model of walking speed (m/s), ELSA waves 2-6

| *Physical Activity levels (ref: sedentary)* | **Coefficients** | **Lower 95% CI** | **Upper 95% CI** |
|---|---|---|---|
| Low | **0.05** | 0.04 | 0.06 |
| Moderate | **0.10** | 0.08 | 0.11 |
| High | **0.12** | 0.11 | 0.13 |
| *Marital status (ref: married)* | | | |
| Separated/Divorced | 0.001 | -0.01 | 0.01 |
| Widowed | -0.003 | -0.01 | 0.01 |
| Never married | **-0.03** | -0.05 | -0.01 |
| *Cohabitation (ref: not living with partner)* | | | |
| Living with partner | 0.01 | -0.01 | 0.03 |
| *CESD- Depression score* | **-0.01** | -0.01 | -0.01 |
| *Smoking status (ref: never smoker)* | | | |
| Ex-Smoker | -0.01 | -0.01 | 0.001 |
| Current smoker | **-0.03** | -0.04 | -0.02 |
| *Date/Day orientation (ref: no correct answer)* | | | |
| 1 correct answer | 0.01 | -0.03 | 0.05 |
| 2 correct answers | **0.03** | -0.01 | 0.07 |
| 3 correct answers | **0.05** | 0.02 | 0.08 |
| All correct answers | **0.05** | 0.02 | 0.09 |
| *Memory test* | **0.01** | 0.004 | 0.01 |
| **Random Part** | | | |
| *Level 2 (Individual)* | | | |
| Intercept variance | 0.02 | | |
| Age centered (linear term) variance | 0.00002 | | |
| Covariance of intercept and age centered | -0.0004 | | |
| *Level 1 (wave)* | | | |
| Intercept | 0.02 | | |
| | | | |
| Number of observations (level 1) | 9656 | | |
| Number of clusters (level 2) | 27509 | | |
| -2*Log Likelihood | -13746.54 | | |

**Boldface** indicates statistical significance ($p<0.05$)

Supplementary Table S4 Selected coefficients (95% CI) from two Fixed Effects Models of walking speed (m/s), ELSA waves 2-6

| **Model 1: excluding chair stand and physical activity** | **Coefficients** | **Lower 95% CI** | **Upper 95% CI** |
|---|---|---|---|
| *Frequency of public transport use(ref: every day or nearly every day )* | | | |
| two or three times a week | -0.02 | -0.04 | 0.01 |
| once a week | **-0.03** | -0.05 | -0.001 |
| did not use because no need | -0.02 | -0.04 | 0.01 |
| did not use because health problems | **-0.08** | -0.11 | -0.05 |
| did not use because structural reasons | -0.02 | -0.04 | 0.01 |

Model 1 includes age, age squared, frequency of public transport use, gender, wealth, car/van access, employment status, social class, urban/rural, mobility difficulties, disability, marital status, cohabitation, CES-D depression score, smoking status memory test, time/date orientation

| **Model 2: including chair stand and physical activity** | **Coefficients** | **Lower 95% CI** | **Upper 95% CI** |
|---|---|---|---|
| *Frequency of public transport use(ref: every day or nearly every day )* | | | |
| two or three times a week | -0.01 | -0.03 | 0.01 |
| once a week | -0.02 | -0.05 | 0.003 |
| did not use because no need | -0.01 | -0.04 | 0.01 |
| did not use because health problems | **-0.06** | -0.09 | -0.03 |
| did not use because structural reasons | -0.01 | -0.04 | 0.01 |

Model 2 includes all the variables in Model 1 as well as chair stand, physical activity and the interaction between physical activity and age. Model 1 is nested within Model 2.

**Boldface** indicates statistical significance (p<0.05)

Supplementary Table S5 Coefficients (95% CI) from the Fixed Effects multinomial model of public transport use, ELSA waves 2-6. Coefficients are log odds of not using public transport for different reasons relative to using public transport at least once a week

| | **no use: no need** | | | **no use: health problems** | | | **no use: structural reasons** | | |
|---|---|---|---|---|---|---|---|---|---|
| | Log odds | Lower 95% CI | Upper 95% CI | Log odds | Lower 95% CI | Upper 95% CI | Log odds | Lower 95% CI | Upper 95% CI |
| *Walking speed* | -0.16 | -0.55 | 0.22 | **-2.39** | -3.29 | -1.48 | -0.26 | -0.64 | 0.12 |
| *Age centered (linear term)* | **0.05** | 0.02 | 0.08 | **0.12** | 0.04 | 0.19 | -0.003 | -0.03 | 0.03 |
| *Age (quadratic term)* | **0.004** | 0.002 | 0.01 | **0.01** | 0.01 | 0.01 | **0.005** | 0.003 | 0.01 |
| *Wealth quintiles (ref: poorest quintile)* | | | | | | | | | |
| Quintile 2 | -0.33 | -0.76 | 0.10 | **-0.96** | -1.80 | -0.11 | -0.20 | -0.69 | 0.29 |
| Quintile 3 | -0.24 | -0.70 | 0.22 | -0.73 | -1.64 | 0.17 | -0.18 | -0.69 | 0.34 |
| Quintile 4 | -0.15 | -0.64 | 0.34 | -0.82 | -1.81 | 0.16 | -0.02 | -0.56 | 0.52 |
| Richest quintile | -0.38 | -0.91 | 0.14 | -1.12 | -2.28 | 0.04 | -0.30 | -0.87 | 0.28 |
| *Access to car/van (ref: yes access)* | | | | | | | | | |
| No access to car/van | **-1.20** | -1.49 | -0.90 | **-0.57** | -0.97 | -0.16 | **-1.02** | -1.35 | -0.68 |
| *Employment status (ref: employed)* | | | | | | | | | |
| Retired | -0.18 | -0.44 | 0.09 | 0.42 | -0.61 | 1.45 | **-0.36** | -0.62 | -0.10 |
| Other | -0.16 | -0.49 | 0.17 | 0.51 | -0.57 | 1.59 | **-0.45** | -0.78 | -0.12 |
| *Social class (ref: Managerial & Prof)* | | | | | | | | | |
| Intermediate occupations | -0.11 | -0.97 | 0.74 | -14.63 | -1940.9 | 1911.7 | -0.23 | -1.05 | 0.58 |
| Self-employed | -0.35 | -1.19 | 0.48 | -2.87 | -6.39 | 0.65 | **-0.89** | -1.72 | -0.06 |
| Lower supervisory & technical | 0.89 | -0.16 | 1.94 | -0.50 | -5.30 | 4.29 | 0.51 | -0.49 | 1.50 |
| Semi-routine & routine | -0.36 | -1.15 | 0.44 | -2.35 | -4.81 | 0.11 | -0.72 | -1.47 | 0.04 |
| *Urban/Rural (ref: Urban)* | | | | | | | | | |
| Town & Fringe | **0.75** | 0.01 | 1.49 | -1.20 | -2.78 | 0.38 | 0.60 | -0.06 | 1.25 |
| Village | 0.38 | -0.62 | 1.37 | 1.23 | -0.39 | 2.84 | 0.59 | -0.24 | 1.43 |
| Hamlet/Isolated | 0.29 | -1.19 | 1.77 | -0.90 | -4.16 | 2.35 | **2.01** | 0.66 | 3.37 |

Supplementary Table S5 continued Coefficients (95% CI) from the Fixed Effects multinomial model of public transport use, ELSA waves 2-6. Coefficients are log odds of not using public transport for different reasons relative to using public transport at least once a week

| | **no use: no need** | | | **no use: health problems** | | | **no use: structural reasons** | | |
|---|---|---|---|---|---|---|---|---|---|
| *Mobility difficulties (ref:none)* | Log odds | Lower 95% CI | Upper 95% CI | Log odds | Lower 95% CI | Upper 95% CI | Log odds | Lower 95% CI | Upper 95% CI |
| 1-3 difficulties | -0.10 | -0.26 | 0.06 | **0.60** | 0.07 | 1.14 | 0.03 | -0.13 | 0.20 |
| 4+ difficulties | -0.06 | -0.33 | 0.21 | **1.43** | 0.83 | 2.03 | 0.26 | -0.01 | 0.54 |
| *Activities of Daily living impairments (ref: none)* | | | | | | | | | |
| At least one ADL impairment | 0.10 | -0.11 | 0.31 | 0.16 | -0.15 | 0.46 | 0.06 | -0.15 | 0.27 |
| *Physical Activity levels (ref: sedentary)* | | | | | | | | | |
| Low | 0.01 | -0.32 | 0.35 | -0.26 | -0.67 | 0.14 | -0.24 | -0.58 | 0.11 |
| Moderate | 0.09 | -0.24 | 0.43 | **-0.57** | -1.04 | -0.09 | -0.26 | -0.62 | 0.09 |
| High | 0.10 | -0.27 | 0.48 | **-0.98** | -1.77 | -0.19 | -0.27 | -0.65 | 0.12 |
| *Marital status (ref: married)* | | | | | | | | | |
| Separated/Divorced | 0.17 | -0.56 | 0.90 | -0.98 | -2.41 | 0.45 | 0.44 | -0.30 | 1.18 |
| Widowed | -0.01 | -0.67 | 0.65 | -0.19 | -1.39 | 1.01 | -0.28 | -0.96 | 0.39 |
| Never married | 0.57 | -0.78 | 1.92 | -0.68 | -3.87 | 2.52 | 0.29 | -1.13 | 1.71 |
| *Cohabitation (ref: not living with partner)* | | | | | | | | | |
| Living with partner | 0.61 | -0.02 | 1.24 | 0.22 | -1.00 | 1.44 | 0.34 | -0.30 | 0.98 |
| *CESD- Depression score* | -0.04 | -0.08 | 0.01 | **0.10** | 0.02 | 0.18 | 0.002 | -0.04 | 0.05 |
| *Smoking status (ref: never smoker)* | | | | | | | | | |
| Ex-Smoker | -0.24 | -0.77 | 0.29 | **-1.18** | -2.21 | -0.14 | -0.27 | -0.82 | 0.27 |
| Current smoker | 0.12 | -0.55 | 0.79 | -0.67 | -1.94 | 0.59 | -0.04 | -0.73 | 0.65 |
| *Date/Day orientation (ref: no correct answer)* | | | | | | | | | |
| 1 correct answer | 1.02 | -0.23 | 2.27 | 1.06 | -0.45 | 2.57 | 0.69 | -0.60 | 1.99 |
| 2 correct answers | 0.70 | -0.40 | 1.79 | 0.38 | -1.01 | 1.77 | 0.53 | -0.61 | 1.67 |
| 3 correct answers | 0.49 | -0.53 | 1.52 | 0.52 | -0.82 | 1.86 | 0.13 | -0.94 | 1.19 |
| All correct answers | 0.56 | -0.46 | 1.58 | 0.55 | -0.79 | 1.88 | 0.11 | -0.95 | 1.17 |
| *Memory test* | 0.00 | -0.03 | 0.02 | 0.00 | -0.05 | 0.05 | -0.01 | -0.04 | 0.01 |

**Boldface** indicates statistical significance ($p<0.05$)

Supplementary Table S6 Coefficients (95% CI) from the Fixed Effects Model of grip strength (kg), ELSA waves 2-6

| | Coefficients | Lower 95% CI | Upper 95% CI |
|---|---|---|---|
| *Intercept* | **28.47** | 26.21 | 30.72 |
| *Age centered (linear term)* | **-0.44** | -0.47 | -0.41 |
| *Age (quadratic term)* | **-0.005** | -0.007 | -0.003 |
| *Frequency of public transport use(ref: every day or nearly every day )* | | | |
| two or three times a week | -0.37 | -0.88 | 0.14 |
| once a week | -0.12 | -0.68 | 0.44 |
| did not use because no need | -0.03 | -0.63 | 0.56 |
| did not use because health problems | **-1.35** | -2.09 | -0.61 |
| did not use because structural reasons | -0.13 | -0.70 | 0.45 |
| *Wealth quintiles (ref: poorest quintile)* | | | |
| Quintile 2 | -0.42 | -1.17 | 0.33 |
| Quintile 3 | -0.35 | -1.15 | 0.46 |
| Quintile 4 | -0.32 | -1.17 | 0.54 |
| Richest quintile | -0.06 | -1.00 | 0.88 |
| *Access to car/van (ref: yes access)* | | | |
| No access to car/van | 0.08 | -0.40 | 0.56 |
| *Employment status (ref: employed)* | | | |
| Retired | -0.22 | -0.67 | 0.22 |
| Other | -0.96 | -1.59 | -0.33 |
| *Social class (ref: Managerial & Prof)* | | | |
| Intermediate occupations | 0.28 | -1.43 | 1.99 |
| Self-employed | -0.39 | -1.44 | 0.65 |
| Lower supervisory & technical | -0.76 | -1.98 | 0.47 |
| Semi-routine & routine | 0.22 | -0.86 | 1.31 |
| *Urban/Rural (ref: Urban)* | | | |
| Town & Fringe | 0.31 | -0.65 | 1.27 |
| Village | 0.08 | -1.16 | 1.32 |
| Hamlet/Isolated | 0.05 | -1.35 | 1.45 |
| *Mobility difficulties (ref: none)* | | | |
| 1-3 difficulties | -0.19 | -0.47 | 0.08 |
| 4+ difficulties | -1.03 | -1.50 | -0.57 |
| *Activities of Daily living impairments (ref: none)* | | | |
| At least one ADL impairment | **-0.69** | -1.07 | -0.30 |
| *Physical Activity levels (ref: sedentary)* | | | |
| Low | **0.69** | 0.07 | 1.30 |
| Moderate | **0.73** | 0.10 | 1.37 |
| High | **0.95** | 0.24 | 1.67 |
| *Marital status (ref: married)* | | | |
| Separated/Divorced | -0.81 | -1.90 | 0.28 |
| Widowed | -0.23 | -1.32 | 0.87 |
| Never married | -1.47 | -3.75 | 0.82 |
| *Cohabitation (ref: not living with partner)* | | | |
| Living with partner | -0.47 | -1.56 | 0.63 |
| *CESD- Depression score* | -0.05 | -0.12 | 0.03 |

Supplementary Table S6 continued Coefficients (95% CI) from the Fixed Effects Model of grip strength (kg), ELSA waves 2-6

| *Smoking status (ref: never smoker)* | Coefficients | Lower 95% CI | Upper 95% CI |
|---|---|---|---|
| Ex-Smoker | 0.46 | -0.35 | 1.27 |
| Current smoker | 0.76 | -0.36 | 1.87 |
| *Date/Day orientation (ref: no correct answer)* | | | |
| 1 correct answer | 0.70 | -1.31 | 2.71 |
| 2 correct answers | **1.94** | 0.17 | 3.71 |
| 3 correct answers | **1.93** | 0.35 | 3.51 |
| All correct answers | **1.87** | 0.30 | 3.44 |
| *Memory test* | 0.02 | -0.02 | 0.07 |
| Number of observations | 21835 | | |
| Adjusted R-squared | 0.87 | | |

**Boldface** indicates statistical significance (p<0.05)

Supplementary Table S7 Coefficients (95% CI) from the Fixed Effects multinomial model of chair stand outcomes, ELSA waves 2-6. Coefficients are log odds of not completing the test or taking longer than the median time to complete the test relative to using completing the chair stand test faster than the median time

| | **Did not complete chair stand test** | | | **Took longer to complete 5/10 chair rises** | | |
|---|---|---|---|---|---|---|
| *Intercept* | **Log odds** | **Lower 95% CI** | **Upper 95% CI** | **Log odds** | **Lower 95% CI** | **Upper 95% CI** |
| *Age centered (linear term)* | **0.09** | 0.06 | 0.11 | **0.10** | 0.09 | 0.12 |
| *Age (quadratic term)* | **0.004** | 0.003 | 0.006 | **0.002** | 0.001 | 0.003 |
| *Frequency of public transport use(ref: every day or nearly every day )* | | | | | | |
| two or three times a week | 0.00 | -0.38 | 0.39 | -0.01 | -0.31 | 0.30 |
| once a week | 0.10 | -0.34 | 0.53 | -0.12 | -0.46 | 0.21 |
| did not use because no need | 0.31 | -0.12 | 0.75 | 0.15 | -0.18 | 0.47 |
| did not use because health problems | **1.28** | 0.63 | 1.92 | 0.43 | -0.16 | 1.02 |
| did not use because structural reasons | **0.45** | 0.02 | 0.89 | 0.05 | -0.27 | 0.38 |
| *Wealth quintiles (ref: poorest quintile)* | | | | | | |
| Quintile 2 | -0.39 | -0.92 | 0.15 | -0.29 | -0.73 | 0.15 |
| Quintile 3 | -0.12 | -0.70 | 0.47 | -0.42 | -0.89 | 0.04 |
| Quintile 4 | -0.32 | -0.95 | 0.31 | -0.46 | -0.94 | 0.03 |
| Richest quintile | -0.24 | -0.94 | 0.46 | -0.42 | -0.93 | 0.10 |
| *Access to car/van (ref: yes access)* | | | | | | |
| No access to car/van | -0.20 | -0.58 | 0.19 | -0.28 | -0.62 | 0.05 |
| *Employment status (ref: employed)* | | | | | | |
| Retired | **0.50** | 0.16 | 0.84 | 0.05 | -0.16 | 0.26 |
| Other | **0.41** | 0.01 | 0.81 | 0.09 | -0.18 | 0.37 |
| *Social class (ref: Managerial & Prof)* | | | | | | |
| Intermediate occupations | 0.78 | -0.13 | 1.70 | -0.08 | -0.67 | 0.50 |
| Self-employed | 0.44 | -0.42 | 1.29 | 0.19 | -0.37 | 0.74 |
| Lower supervisory & technical | -0.14 | -1.19 | 0.90 | -0.25 | -0.97 | 0.46 |
| Semi-routine & routine | 0.16 | -0.66 | 0.99 | 0.48 | -0.05 | 1.01 |

Supplementary Table S7 continued Coefficients (95% CI) from the Fixed Effects multinomial model of chair stand outcomes, ELSA waves 2-6. Coefficients are log odds of not completing the test or taking longer than the median time to complete the test relative to using completing the chair stand test faster than the median time

| | **Did not complete chair stand test** | | | **Took longer to complete 5/10 chair rises** | | |
|---|---|---|---|---|---|---|
| *Urban/Rural (ref: Urban)* | **Log odds** | **Lower 95% CI** | **Upper 95% CI** | **Log odds** | **Lower 95% CI** | **Upper 95% CI** |
| Town & Fringe | 0.09 | -0.85 | 1.03 | 0.26 | -0.40 | 0.92 |
| Village | -0.69 | -1.63 | 0.26 | 0.19 | -0.55 | 0.93 |
| Hamlet/Isolated | **1.54** | 0.03 | 3.05 | 0.47 | -0.49 | 1.43 |
| *Mobility difficulties (ref: none)* | | | | | | |
| 1-3 difficulties | 0.10 | -0.15 | 0.34 | 0.11 | -0.05 | 0.26 |
| 4+ difficulties | **0.83** | 0.46 | 1.19 | **0.37** | 0.10 | 0.63 |
| *Activities of Daily living impairments (ref: none)* | | | | | | |
| At least one ADL impairment | **0.46** | 0.18 | 0.73 | **0.06** | -0.17 | 0.29 |
| *Physical Activity levels (ref: sedentary)* | | | | | | |
| Low | -0.14 | -0.63 | 0.35 | 0.20 | -0.25 | 0.66 |
| Moderate | -0.48 | -0.98 | 0.01 | -0.09 | -0.55 | 0.36 |
| High | -0.21 | -0.76 | 0.33 | -0.11 | -0.58 | 0.37 |
| *Marital status (ref: married)* | | | | | | |
| Separated/Divorced | -0.38 | -1.22 | 0.46 | -0.38 | -1.00 | 0.25 |
| Widowed | -0.55 | -1.37 | 0.28 | -0.37 | -0.97 | 0.23 |
| Never married | -0.31 | -1.93 | 1.30 | -1.22 | -2.45 | 0.01 |
| *Cohabitation (ref: not living with partner)* | | | | | | |
| Living with partner | -0.66 | -1.45 | 0.13 | -0.29 | -0.85 | 0.27 |
| *CESD- Depression score* | **0.07** | 0.02 | 0.13 | 0.01 | -0.04 | 0.05 |
| *Smoking status (ref: never smoker)* | | | | | | |
| Ex-Smoker | -0.30 | -0.99 | 0.39 | 0.15 | -0.35 | 0.66 |
| Current smoker | -0.58 | -1.43 | 0.27 | -0.01 | -0.63 | 0.61 |

Supplementary Table S7 continued Coefficients (95% CI) from the Fixed Effects multinomial model of chair stand outcomes, ELSA waves 2-6. Coefficients are log odds of not completing the test or taking longer than the median time to complete the test relative to using completing the chair stand test faster than the median time

| | **Did not complete chair stand test** | | | **Took longer to complete 5/10 chair rises** | | |
|---|---|---|---|---|---|---|
| Date/Day orientation (ref: no correct answer) | **Log odds** | **Lower 95% CI** | **Upper 95% CI** | **Log odds** | **Lower 95% CI** | **Upper 95% CI** |
| 1 correct answer | 0.01 | -2.15 | 2.17 | -0.02 | -1.72 | 1.67 |
| 2 correct answers | -0.89 | -2.59 | 0.80 | -0.01 | -1.43 | 1.40 |
| 3 correct answers | -0.58 | -2.19 | 1.03 | 0.34 | -1.01 | 1.69 |
| All correct answers | -0.68 | -2.28 | 0.92 | 0.21 | -1.14 | 1.55 |
| *Memory test* | -0.03 | -0.06 | 0.01 | 0.00 | -0.03 | 0.02 |
| Number of observations | 12463 | | | | | |
| Adjusted R-squared | 0.10 | | | | | |

**Boldface** indicates statistical significance (p<0.05)

Supplementary Table S8 Coefficients (95% CI) from the Fixed Effects multinomial model of physical activity, ELSA waves 2-6. Coefficients are log odds of sedentary/low/moderate physical activity, relative to being in the high physical activity category

| | **Sedentary physical activity** | | | **Low physical activity** | | | **Moderate physical activity** | | |
|---|---|---|---|---|---|---|---|---|---|
| | **Log odds** | **Lower 95% CI** | **Upper 95% CI** | **Log odds** | **Lower 95% CI** | **Upper 95% CI** | **Log odds** | **Lower 95% CI** | **Upper 95% CI** |
| *Age centered (linear term)* | **0.17** | 0.11 | 0.22 | **0.10** | 0.06 | 0.13 | 0.02 | -0.01 | 0.05 |
| *Age (quadratic term)* | **0.006** | 0.004 | 0.007 | **0.005** | 0.004 | 0.006 | **0.002** | 0.001 | 0.003 |
| *Frequency of public transport use(ref: every day or nearly every day )* | | | | | | | | | |
| two or three times a week | -0.18 | -0.64 | 0.28 | **-0.33** | -0.62 | -0.03 | -0.15 | -0.40 | 0.10 |
| once a week | 0.06 | -0.47 | 0.60 | -0.24 | -0.57 | 0.08 | -0.10 | -0.37 | 0.17 |
| did not use because no need | -0.22 | -0.75 | 0.30 | -0.16 | -0.49 | 0.16 | -0.08 | -0.35 | 0.19 |
| did not use because health problems | 1.37 | 0.65 | 2.09 | 0.81 | 0.23 | 1.39 | 0.39 | -0.17 | 0.94 |
| did not use because structural reasons | 0.10 | -0.43 | 0.63 | -0.10 | -0.42 | 0.22 | -0.08 | -0.35 | 0.19 |
| *Interaction between Age (linear term) and Frequency of public transport use (ref: Nearly/Every day use)* | | | | | | | | | |
| Age*two or three times a week | 0.04 | -0.01 | 0.08 | 0.03 | -0.001 | 0.07 | 0.03 | -0.001 | 0.06 |
| Age*once a week | 0.04 | -0.01 | 0.09 | 0.02 | -0.02 | 0.06 | 0.02 | -0.01 | 0.05 |
| Age*did not use because no need | **0.06** | 0.01 | 0.11 | 0.02 | -0.01 | 0.06 | **0.04** | 0.01 | 0.07 |
| Age*did not use because health problems | 0.06 | -0.003 | 0.13 | 0.02 | -0.03 | 0.08 | 0.02 | -0.04 | 0.07 |
| Age*did not use because structural reasons | **0.06** | 0.01 | 0.11 | 0.03 | -0.01 | 0.06 | **0.04** | 0.01 | 0.07 |
| *Wealth quintiles (ref: poorest quintile)* | | | | | | | | | |
| Quintile 2 | 0.21 | -0.36 | 0.78 | 0.34 | -0.03 | 0.72 | **0.35** | 0.04 | 0.66 |
| Quintile 3 | -0.02 | -0.64 | 0.60 | 0.21 | -0.20 | 0.61 | 0.30 | -0.04 | 0.63 |
| Quintile 4 | -0.28 | -0.95 | 0.39 | 0.02 | -0.40 | 0.45 | 0.26 | -0.09 | 0.61 |
| Richest quintile | -0.66 | -1.45 | 0.13 | 0.03 | -0.43 | 0.49 | 0.29 | -0.08 | 0.67 |
| *Access to car/van (ref: yes access)* | | | | | | | | | |
| No access to car/van | **0.40** | 0.05 | 0.76 | 0.08 | -0.21 | 0.38 | -0.01 | -0.28 | 0.25 |
| *Employment status (ref: employed)* | | | | | | | | | |
| Retired | **0.79** | 0.24 | 1.33 | 0.06 | -0.15 | 0.28 | 0.01 | -0.15 | 0.16 |
| Other | **0.73** | 0.16 | 1.30 | 0.08 | -0.18 | 0.34 | 0.02 | -0.18 | 0.23 |

Supplementary Table S8 continued Coefficients (95% CI) from the Fixed Effects multinomial model of physical activity, ELSA waves 2-6. Coefficients are log odds of sedentary/low/moderate physical activity, relative to being in the high physical activity category

| | **Sedentary physical activity** | | | **Low physical activity** | | | **Moderate physical activity** | | |
|---|---|---|---|---|---|---|---|---|---|
| *Social class (ref: Managerial & Prof)* | **Log odds** | **Lower 95% CI** | **Upper 95% CI** | **Log odds** | **Lower 95% CI** | **Upper 95% CI** | **Log odds** | **Lower 95% CI** | **Upper 95% CI** |
| Intermediate occupations | -0.97 | -2.46 | 0.52 | -0.53 | -1.09 | 0.02 | -0.21 | -0.60 | 0.17 |
| Self-employed | 0.09 | -1.37 | 1.55 | **-0.79** | -1.36 | -0.22 | **-0.47** | -0.85 | -0.09 |
| Lower supervisory & technical | **-1.85** | -3.52 | -0.18 | -0.65 | -1.36 | 0.07 | -0.06 | -0.51 | 0.40 |
| Semi-routine & routine | -1.23 | -2.67 | 0.21 | -0.35 | -0.87 | 0.18 | -0.19 | -0.54 | 0.16 |
| *Urban/Rural (ref: Urban)* | | | | | | | | | |
| Town & Fringe | -0.62 | -1.81 | 0.57 | 0.41 | -0.21 | 1.04 | 0.28 | -0.20 | 0.76 |
| Village | 0.23 | -1.22 | 1.69 | 0.12 | -0.53 | 0.76 | -0.42 | -0.91 | 0.08 |
| Hamlet/Isolated | -0.01 | -1.77 | 1.76 | -0.22 | -1.16 | 0.73 | -0.35 | -1.09 | 0.39 |
| *Mobility difficulties (ref: none)* | | | | | | | | | |
| 1-3 difficulties | -0.05 | -0.36 | 0.26 | **0.25** | 0.10 | 0.39 | **0.15** | 0.03 | 0.26 |
| 4+ difficulties | **0.86** | 0.46 | 1.26 | **0.97** | 0.71 | 1.23 | **0.52** | 0.29 | 0.75 |
| *Activities of Daily living impairments (ref: none)* | | | | | | | | | |
| At least one ADL impairment | **0.63** | 0.36 | 0.90 | **0.27** | 0.07 | 0.48 | 0.07 | -0.11 | 0.26 |
| *Marital status (ref: married)* | | | | | | | | | |
| Separated/Divorced | 0.48 | -0.35 | 1.31 | 0.25 | -0.29 | 0.79 | 0.23 | -0.20 | 0.65 |
| Widowed | 0.12 | -0.74 | 0.98 | -0.02 | -0.58 | 0.55 | 0.12 | -0.34 | 0.59 |
| Never married | 1.07 | -0.50 | 2.63 | -0.06 | -1.01 | 0.89 | -0.05 | -0.78 | 0.68 |
| *Cohabitation (ref: not living with partner)* | | | | | | | | | |
| Living with partner | 0.62 | -0.23 | 1.47 | 0.45 | -0.08 | 0.98 | 0.23 | -0.19 | 0.65 |
| *CESD- Depression score* | **0.19** | 0.14 | 0.25 | **0.14** | 0.10 | 0.18 | **0.06** | 0.03 | 0.09 |
| *Smoking status (ref: never smoker)* | | | | | | | | | |
| Ex-Smoker | -0.47 | -1.31 | 0.36 | -0.33 | -0.81 | 0.15 | -0.28 | -0.67 | 0.11 |
| Current smoker | -0.63 | -1.60 | 0.34 | -0.57 | -1.16 | 0.01 | -0.28 | -0.76 | 0.20 |

Supplementary Table S8 continued Coefficients (95% CI) from the Fixed Effects multinomial model of physical activity, ELSA waves 2-6. Coefficients are log odds of sedentary/low/moderate physical activity, relative to being in the high physical activity category

| | **Sedentary physical activity** | | | **Low physical activity** | | | **Moderate physical activity** | | |
|---|---|---|---|---|---|---|---|---|---|
| Date/Day orientation (ref: no correct answer) | **Log odds** | **Lower 95% CI** | **Upper 95% CI** | **Log odds** | **Lower 95% CI** | **Upper 95% CI** | **Log odds** | **Lower 95% CI** | **Upper 95% CI** |
| 1 correct answer | 0.26 | -1.42 | 1.94 | -0.36 | -1.91 | 1.18 | -0.63 | -2.12 | 0.85 |
| 2 correct answers | -0.70 | -2.20 | 0.79 | -0.63 | -2.02 | 0.76 | -0.70 | -2.03 | 0.63 |
| 3 correct answers | -0.91 | -2.34 | 0.52 | -0.55 | -1.88 | 0.78 | -0.57 | -1.84 | 0.71 |
| All correct answers | -1.05 | -2.48 | 0.37 | -0.62 | -1.95 | 0.70 | -0.62 | -1.89 | 0.65 |
| *Memory test* | **-0.05** | -0.09 | -0.02 | **-0.03** | -0.05 | -0.004 | -0.02 | -0.03 | 0.003 |
| Number of observations | 32,276 | | | | | | | | |
| Adjusted R-squared | 0.06 | | | | | | | | |

**Boldface** indicates statistical significance (p<0.05)

Supplementary Table S9 Coefficients (95% CI) from the Multilevel Growth Curve Model of walking speed (m/s), ELSA waves 2-6: Only participants with 3 or more waves of data

| **Fixed Part** | **Coefficients** | **Lower 95% CI** | **Upper 95% CI** |
|---|---|---|---|
| *Intercept* | **0.80** | 0.75 | 0.86 |
| *Age centered (linear term)* | **-0.01** | -0.01 | -0.01 |
| *Age (quadratic term)* | **-0.0002** | -0.0003 | -0.0002 |
| *Frequency of public transport use(ref: every day or nearly every day )* | | | |
| two or three times a week | -0.01 | -0.02 | 0.0003 |
| once a week | **-0.01** | -0.03 | -0.001 |
| did not use because no need | **-0.02** | -0.03 | -0.003 |
| did not use because health problems | **-0.09** | -0.11 | -0.08 |
| did not use because structural reasons | **-0.011** | -0.023 | 0.001 |
| *Interaction between Age (linear term) and Frequency of public transport use (ref: nearly/every day use)* | | | |
| Age*two or three times a week | -0.0003 | -0.0018 | 0.0013 |
| Age*once a week | -0.0016 | -0.0032 | 0.00003 |
| Age*did not use because no need | -0.0012 | -0.0027 | 0.0004 |
| Age*did not use because health problems | 0.0005 | -0.0014 | 0.0024 |
| Age*did not use because structural reasons | **-0.0019** | -0.0034 | -0.0004 |
| *Sex (ref: men)* | | | |
| Women | -0.03 | -0.03 | -0.02 |
| *Wealth quintiles (ref: poorest quintile)* | | | |
| Quintile 2 | **0.02** | 0.01 | 0.03 |
| Quintile 3 | **0.03** | 0.02 | 0.04 |
| Quintile 4 | **0.05** | 0.04 | 0.07 |
| Richest quintile | **0.08** | 0.06 | 0.09 |
| *Access to car/van (ref: yes access)* | | | |
| No access to car/van | **-0.02** | -0.03 | -0.01 |
| *Employment status (ref: employed)* | | | |
| Retired | -0.01 | -0.02 | 0.002 |
| Other | **-0.02** | -0.03 | -0.003 |
| *Social class (ref: Managerial & Prof)* | | | |
| Intermediate occupations | **-0.02** | -0.04 | -0.01 |
| Self-employed | **-0.02** | -0.04 | -0.01 |
| Lower supervisory & technical | **-0.04** | -0.05 | -0.02 |
| Semi-routine & routine | **-0.05** | -0.06 | -0.04 |
| *Urban/Rural (ref: Urban)* | | | |
| Town & Fringe | 0.01 | -0.004 | 0.02 |
| Village | **0.02** | 0.01 | 0.03 |
| Hamlet/Isolated | **0.02** | 0.00 | 0.04 |
| *Mobility difficulties (ref: none)* | | | |
| 1-3 difficulties | **-0.03** | -0.04 | -0.03 |
| 4+ difficulties | **-0.11** | -0.12 | -0.10 |
| *Activities of Daily living impairments (ref: none)* | | | |
| At least one ADL impairment | **-0.05** | -0.06 | -0.04 |

Supplementary Table S9 continued Coefficients (95% CI) from the Multilevel Growth Curve Model of walking speed (m/s), ELSA waves 2-6: Only participants with 3 or more waves of data

| *Physical Activity levels (ref: sedentary)* | **Coefficients** | **Lower 95% CI** | **Upper 95% CI** |
|---|---|---|---|
| Low | **0.04** | 0.03 | 0.06 |
| Moderate | **0.09** | 0.07 | 0.10 |
| High | **0.11** | 0.09 | 0.12 |
| *Marital status (ref: married)* | | | |
| Separated/Divorced | **0.03** | 0.003 | 0.05 |
| Widowed | 0.02 | -0.01 | 0.04 |
| Never married | -0.004 | -0.03 | 0.03 |
| *Cohabitation (ref: not living with partner)* | | | |
| Living with partner | 0.02 | -0.002 | 0.04 |
| *CESD- Depression score* | **-0.01** | -0.01 | -0.01 |
| *Smoking status (ref: never smoker)* | | | |
| Ex-Smoker | **-0.01** | -0.02 | -0.002 |
| Current smoker | **-0.03** | -0.04 | -0.01 |
| *Date/Day orientation (ref: no correct answer)* | | | |
| 1 correct answer | 0.02 | -0.04 | 0.08 |
| 2 correct answers | 0.04 | -0.01 | 0.09 |
| 3 correct answers | 0.05 | -0.001 | 0.09 |
| All correct answers | **0.05** | 0.004 | 0.10 |
| *Memory test* | **0.004** | 0.003 | 0.01 |
| **Random Part** | | | |
| *Level 2 (Individual)* | | | |
| Intercept variance | 0.02 | | |
| Age centered (linear term) variance | 0.00003 | | |
| Covariance of intercept and age centered | -0.0003 | | |
| *Level 1 (wave)* | | | |
| Intercept | 0.02 | | |
| | | | |
| Number of observations (level 1) | 5547 | | |
| Number of clusters (level 2) | 21573 | | |
| -2*Log Likelihood | -11874.03 | | |

**Boldface** indicates statistical significance (p<0.05)
