# Supplementary material for: Does public transport use prevent declines in walking speed among older adults living in England? A prospective cohort study
Source: BMJ Open. 2017 Sep 28;7(9):e017702. doi: 10.1136/bmjopen-2017-017702 (PMC5652510; doi:10.1136/bmjopen-2017-017702)
Supplement: Author's manuscript [file bmjopen-2017-017702.draft_revisions.pdf]

BMJ Open

BMJ Open is committed to open peer review. As part of this commitment we make the peer review history of every article we publish publicly available.

When an article is published we post the peer reviewers' comments and the authors' responses online. We also post the versions of the paper that were used during peer review. These are the versions that the peer review comments apply to.

The versions of the paper that follow are the versions that were submitted during the peer review process. They are not the versions of record or the final published versions. They should not be cited or distributed as the published version of this manuscript.

BMJ Open is an open access journal and the full, final, typeset and author-corrected version of record of the manuscript is available on our site with no access controls, subscription charges or pay-per-view fees (http://bmjopen.bmj.com).

If you have any questions on BMJ Open's open peer review process please email info.bmjopen@bmj.com

BMJ Open

Frequent public transport use reduces age-related decline in walking speed among older adults living in England

Journal:	*BMJ Open*
Manuscript ID	bmjopen-2017-017702
Article Type:	Research
Date Submitted by the Author:	11-May-2017
Complete List of Authors:	Rouxel, Patrick; University College London Institute of Education, Social Science<br>Webb, Elizabeth; University College London, International Centre for Lifecourse Studies, Epidemiology and Public Health<br>Chandola, Tarani; University of Manchester, The Cathie Mash Centre for Census and Survey Research
**Primary Subject Heading**:	Epidemiology
Secondary Subject Heading:	Public health
Keywords:	public transport, ageing, walking speed, physical activity

SCHOLARONE™
Manuscripts

For peer review only

Frequent public transport use reduces age-related decline in walking speed among older adults living in England

Patrick Rouxel [1], Elizabeth Webb [2] and Tarani Chandola [3]

[1]CLOSER, Department of Social Science, University College London Institute of Education, London, UK, ORCID: 0000-0003-0330-554X, [2] International Centre for Lifecourse Studies, Department of Epidemiology and Public Health, University College London, London, UK, ORCID: 0000-0001-6106-5367 and [3]CMIST and Social Statistics, University of Manchester, UK, ORCID: 0000-0002-1864-3413

Corresponding author

Patrick Rouxel, CLOSER, Department of Social Science, University College London Institute of Education, 55-59 Gordon Square, WC1H 0NU, London, UK. E-mail: patrick.rouxel@ucl.ac.uk; phone: +44 7527565347

Word count 2,832

For peer review only

Abstract

Objectives: Although there is some evidence that public transport use confers public health benefits, the evidence is limited by cross-sectional study designs and health-related confounding factors. This study examines the effect of public transport use on changes in walking speed among older adults living in England, comparing frequent users of public transport to their peers who did not use public transport because of structural barriers (poor public transport infrastructure), or through choice.

Design: Prospective cohort study.

Setting: England, UK

Participants: Older adults aged 60 or older eligible for the walking speed test. 6,246 individuals at wave 2 (2004-05); 5,909 individuals at wave 3 (2006-07); 7,321 individuals at wave 4 (2008-09); 7,535 individuals at wave 5 (2010-11); and 7,664 individuals at wave 6 (2012-13) of the English Longitudinal Study of Ageing.

Main outcome measure: The walking speed was estimated from the time taken to walk 2.4 metres. Fixed effects models and growth curve models were used to examine the associations between public transport use and walking speed.

Results: Older adults who did not use public transport through choice or because of structural reasons had slower walking speeds [-0.02 m/s (95%CI -0.03,-0.003) and -0.02 m/s (95%CI -0.03,-0.01), respectively] and took an extra 0.07 seconds to walk 2.4 meters compared to their peers who used public transport frequently. The age-related trajectories of decline in walking speed were slower for frequent users of public transport compared to non-users.

Conclusions: Frequent use of public transport may prevent age-related decline in physical capability by promoting physical activity among older adults. The association between public transport use and slower decline in walking speed among older adults is unlikely to be confounded by health related selection factors. Improving access to good quality public transport could improve the health of older adults.

Strengths and limitations of the study

- Previous cross-sectional research on the protective role of public transport use in relation to age related functional declines may have been biased by health selection processes as people age.
- Older adults with deteriorating health are less likely to use public transport and their poor health could determine their functional decline, rather than their lack of public transport use.

For peer review only

- This longitudinal study of over 7,000 adults living in England suggests that the inference that frequent use of public transport may prevent age-related decline in physical capability is robust to such potential biases.
- While this is not a causal analysis, we have controlled for within- and between-person factors that could bias the association between public transport use and walking speed decline.
- Given the current context of cuts in public transport availability in England, this research suggests that such cuts may result in faster declines in physical functioning as people age.

For peer review only

Introduction

Declines in walking speed and grip strength are markers of ageing and are associated with all-cause mortality [1] and poorer health in older populations. [2] Maintaining physical capability is a prerequisite for older people to engage independently in many social activities [3] and for reducing social exclusion. [4 5]

Compelling evidence supports the efficacy of physical activity in maintaining muscle mass, strength and function in older adults. [6 7] Older adults may add regular physical activity into daily life by walking and maintaining balance on moving vehicles such as bus or train. [6] Public transport related physical activity was associated with a larger reduction in mortality for older adults (above 70 years) compared to younger adults. [8] Public transport users are more physically active than non-users of public transport. [9-11]

Poor physical capability is an important barrier to the use of public transport by older people. Other barriers include the costs and poor quality of public transport. [5 12 13] It is particularly important to consider such barriers to public transport use given the current context of cuts to local bus services in England. [14]

Despite concessionary bus passes in the UK offering free bus travel to those over the State Pension Age, in England a third of older adults aged over 65 never use public transport, whilst another third use it very infrequently. [15] Evidence from the English Longitudinal Study of Ageing (ELSA) [16 17] and other studies [5 18] showed that free bus travel for older people was associated with increased active travel and raised physical activity.

Although walking speed declines with age, [19 20] there is some cross-sectional evidence that older women with a free bus pass use public transport more often and have faster walking speeds than those who do not hold a bus pass. [16] Moreover, public transport use is associated with lower levels of obesity and may have a protective effect against becoming obese. [17]

However, the key limitation in existing evidence is that negative health selection is not taken into account. Older adults with deteriorating health may be less likely to use public transport and their poor health could determine the decline in walking speed, rather than their lack of public transport use. There is also a lack of analyses on different reasons for not using public transport. Older adults may not use public transport because of health

For peer review only

problems and disabilities [3 21] or because reliable public transport is not available, [12] or because they prefer using their own vehicles. [22] Separating out these reasons for not using public transport is important: if a negative effect of not using public transport on walking speed is observed among older adults who do not use public transport due to structural reasons (poor public transport infrastructure) or through choice, this suggests the observed association between public transport use and walking speed is not confounded by health.

An additional test of the specificity of the public transport-walking speed association is whether similar associations are observed between public transport use and grip strength. As public transport use is unlikely to have an impact on grip strength, any association between public transport use and grip strength is likely to be caused by confounding factors.

Our paper will address the following research questions:

RQ1: Do older adults who frequently use public transport have faster walking speeds than those who do not use public transport for structural reasons or through choice?

RQ2: Is the association of public transport use with muscle function deficit specific to walking speed? Does public transport use also predict stronger grip strength?

RQ3: Are declines of walking speed with age slower for older adults who use public transport often, compared to their peers who do not use public transport for structural reasons or through choice?

Methods

Data

The data come from waves 2 to 6 of the English Longitudinal Study of Ageing (ELSA), where individuals aged 50 and over living in private households in England were followed and re-interviewed every two years. [23] The ELSA sample was refreshed at waves 3, 4, and 6 to ensure the sample remained representative of the population aged 50 and older. The National Research Ethics Service approved the study, and all participants gave their informed consent. We used data from older adults aged 60 or older - those who were eligible for the walking speed test - consisting of 6,246 individuals at wave 2 (2004-05); 5,909 individuals at wave 3 (2006-07); 7,321 individuals at wave 4 (2008-09); 7,535 individuals at wave 5 (2010-11); and 7,664 individuals at wave 6 (2012-13). Data from wave 1 were omitted, since the reasons why people did not use public transport were not asked. Data were collected though face-to-face interview using computer-assisted personal interview, and a self-

For peer review only - http://bmjopen.bmj.com/site/about/guidelines.xhtml

completion questionnaire. In addition, there was a nurse visit at waves 2, 4 and 6, which assessed grip strength and collected blood samples and anthropometric measurements. The ELSA data and documentation are publicly available from the UK Data Service. (http://www.esds.ac.uk/findingData/snDescription.asp?sn=5050).

Variables

Walking speed (m/s): participants aged 60 and older were asked to twice walk a distance of 8 feet (2.4m) at their usual pace. Walking aids were permitted. We used the mean walking speed based on the two timings.

Grip strength (kg): participants were asked to squeeze a grip gauge up to three times with each hand. We used the mean of the three measurements for the dominant hand.

Frequency of public transport use: participants were asked how often they used public transport. In addition, those who rarely or never used public transport were asked to provide the reasons of not using public transport more often. We derived the frequency variable as follows: every day/nearly every day; 2-3 times a week; once a week; no use because did not need to (or in other words through "choice"); no use because of health problems; no use because of structural reasons. Structural reasons, for these purposes, include: not convenient, does not go where they want, infrequent, unreliable, too expensive, too dirty, fear of crime.

At wave 2, the frequency of use variable had different responses which we mapped onto the later wave responses as follows: a lot=nearly every day; quite often=2-3 times a week; sometimes=once a week; rarely/never=no use. [17]

Covariates

A quadratic term for continuous age was specified to characterize non-linear age effects.

Other covariates included gender; marital status (married; divorced/separated; widowed; never married), cohabiting status (currently living with a partner or not); urban/rural areas (urban; town; village; hamlet); quintiles of non-pension wealth; access to car; employment status (employed; retired; other); National Statistics Socio-economic Classification (NS-SEC) social class; smoking (never; former; current). Mobility difficulties were assessed by asking participants whether they had difficulties with 10 common functions (e.g. walking 100 yards; climbing several flights of stairs without resting; climbing one flight of stairs without resting; lifting or

For peer review only - http://bmjopen.bmj.com/site/about/guidelines.xhtml

carrying weights). We derived a variable with three categories: no difficulties; 1-3 difficulties, 4 or more difficulties. The number of functional limitations in Activities of Daily Living (ADLs) provides an indication of disability. The ADLs scale [24] comprises problems with dressing, walking, bathing, eating (such as cutting up food), getting out of bed, and using the toilet. We derived a binary variable no limitations in ADLs; at least one limitation in ADLs. Depressive symptoms were measured using the eight-item version of the Center for Epidemiologic Study Depression (CES-D) scale. [25] Participants were asked how often they participated in mild, moderate or vigorous physical activity including participation in occupations that involve physical work. Based on their responses, they were classified into the following categories: sedentary; low; moderate or high level of physical activity. [26]

Analysis

For RQs 1 and 2: Fixed effects models were used to regress walking speed and grip strength on the frequency of public transport. These models investigate the effects of within individual (time varying) changes in public transport use on changes in walking speed and grip strength, taking into account other time varying covariates. We estimated these models in Stata 14.0.

For RQ3: We used multilevel (random effects) growth curve models to estimate age-related trajectories of walking speed for different categories of public transport use. These models can be used to describe how different trajectories of walking speed change with age, by interacting age with the frequency of public transport use. Individual trajectories (at level 2) of walking speed (at level 1) are estimated, with a random slope of age (at level 2) characterizing differences in individual trajectories of walking speed. We estimated these models in MLwiN 2.1.

Missing data

There are 29,894 observations of walking speed between waves 2 to 6, which reduced to 27,525 observations in the statistical models due to missing data in the covariates.

The ELSA study team provides longitudinal weights for the core ELSA members present at each wave from the first wave. We did not use these for our analyses as, since they are not available for the ELSA refreshment sample members, using the longitudinal weights would have reduced our sample size by more than half. Instead,

For peer review only

we used the wave specific cross-sectional weights in both the fixed effects and multilevel growth curve, in order to make our analyses representative of non-institutionalized older adults living in England.

Results

Table 1 shows the distribution of all the variables used in the analysis. The mean walking speed was 0.85 m/s and the mean age was 70.5. 34% of ELSA respondents across waves 2-6 reported taking public transport at least once a week, while 33% reported not using public transport because of structural reasons. Most of the sample (73%) lived in urban areas, and almost 60% reported at least one difficulty with mobility.

For peer review only

Table 1 Distribution (percentage/mean) of all the variables in the analysis, observations (n) across ELSA waves 2-6 among respondents with walking speed data

Variables	%/Mean (sd)	n obs across 6 waves	Variables	%/Mean (sd)	n obs across 6 waves
**Walking speed (m/s)**	0.85 (0.28)	29894	**Age**	70.47 (7.70)	29,894
**Frequency of public transport use**			**CES-D depression score**	1.34 (1.82)	29579
every day or nearly every day	8.5%	2,540	**Social class**		
two or three times a week	14.4%	4,300	managerial	32.2%	9,458
once a week	11.2%	3,342	intermediate	14.0%	4,093
no use: no need	27.9%	8,337	self-employed	11.7%	3,434
no use: health problems	5.5%	1,631	lower supervisory	10.5%	3,083
no use: structural reasons	32.6%	9,741	semi-routine	31.6%	9,270
**Gender**			**Functional status (ADLS)**		
male	45.9%	13,728	no limitation	82.1%	24,538
female	54.1%	16,166	at least 1 limitation	17.9%	5,355
**Wealth quintiles**			**Urban/rural**		
poorest	16.4%	4,660	urban	72.6%	21,679
2nd	18.8%	5,327	town	12.6%	3,771
3rd	21.2%	5,999	village	10.8%	3,213
4th	21.6%	6,135	hamlet	4.0%	1,198
richest	22.0%	6,245			
**Access to car**			**Cohabiting status**		
yes access to car	83.4%	24,944	living alone	33.2%	9,909
no access to car	16.6%	4,950	living with partner	66.9%	19,985
**Employment status**			**Smoking status**		
employed	16.8%	5,010	never smoked	36.6%	10,931
retired	74.0%	22,081	ex-smoker	52.1%	15,551
other	9.2%	2,748	current smoker	11.2%	3,351
**Marital status**			**Physical activity**		
married	65.0%	19,429	sedentary	5.0%	1,503
separated/divorced	9.8%	2,923	low	26.1%	7,770
widowed	20.5%	6,117	moderate	51.1%	15,236
never married	4.8%	1,421	high	17.8%	5,321
**Mobility difficulties**					
none	40.7%	12,155			
1 to 3	37.6%	11,233			
4 or more	21.8%	6,502			

Table 2 reports the mean walking speed by frequency of public transport use at waves 2-6. Those who did not use public transport due to health problems had the slowest walking speed, while those who did not use public transport because of structural reasons had the fastest walking speed. There was no pattern of slower walking speed in later waves due to refreshment samples which resulted in the mean age at wave 6 being younger than at wave 2.

For peer review only

Table 2 Weighted mean (95% CI) of walking speed (m/s) by frequency of public transport use at waves 2-6 of ELSA

	every day or nearly every day	two or three times a week	once a week	no use: no need	no use: health problems	no use: structural reasons
wave 2	0.79 (0.77, 0.81)	0.79 (0.77, 0.81)	0.85 (0.84, 0.87)	0.84 (0.83, 0.86)	0.45 (0.42, 0.47)	0.90 (0.89, 0.92)
n	652	695	1,071	1,475	333	1,252
wave 3	0.77 (0.75, 0.80)	0.79 (0.77, 0.80)	0.79 (0.76, 0.81)	0.84 (0.82, 0.85)	0.44 (0.42, 0.47)	0.90 (0.88, 0.91)
n	389	694	462	1,431	289	1,863
wave 4	0.78 (0.76, 0.81)	0.80 (0.78, 0.82)	0.85 (0.83, 0.88)	0.84 (0.83, 0.85)	0.47 (0.44, 0.5)	0.89 (0.88, 0.91)
n	499	956	556	1,584	295	2,157
wave 5	0.81 (0.78, 0.83)	0.84 (0.83, 0.86)	0.86 (0.84, 0.88)	0.88 (0.87, 0.89)	0.47 (0.44, 0.49)	0.91 (0.90, 0.93)
n	472	935	624	1,610	315	2,148
wave 6	0.81 (0.79, 0.84)	0.84 (0.83, 0.86)	0.84 (0.82, 0.87)	0.90 (0.88, 0.91)	0.49 (0.46, 0.51)	0.93 (0.91, 0.94)
n	450	906	547	1,884	346	1,881

Table 3 reports the results of the fixed effects and multilevel growth curve (random effects) models of walking speed. Only the coefficients for age and the frequency variable are shown- the full models are shown in online Supplementary file Tables 1 and 2. In the fixed effects model, the linear and quadratic terms of age were negative, suggesting that as age increased, walking speed declined faster. Compared to the reference group who used public transport nearly every day, the coefficients for all the other frequency groups were negative, suggesting that using public transport nearly every day had a protective effect on walking speed. Those who did not use public transport because of health problems had the biggest decline in walking speed (-0.06 m/s), but those who did not use public transport because they did not need to, or because of structural reasons were also more likely to have a decline in walking speed (-0.02 m/s). A difference of 0.02 m/s is an extra 0.07 seconds taken to do the walking speed test. The interaction between age and frequency of use of public transport was not significant in the fixed effects model.

For peer review only

Table 3 Selected coefficients (95% CI) from the Fixed Effects and Growth Curve Models of walking speed (m/s), ELSA waves 2-6

	Fixed Effects Model	Growth Curve Model 1	Growth Curve Model 2
**Fixed Part**			
Intercept	**0.879 (0.831, 0.926)**	**0.871 (0.844, 0.899)**	**0.863 (0.835, 0.892)**
Age in years centered (linear term)	**-0.008 (-0.01, -0.007)**	**-0.007 (-0.008, -0.007)**	**-0.006 (-0.008, -0.005)**
Age (quadratic term)	**-0.0003 (-0.0004, -0.0003)**	-0.0002 (0.00002, -0.0002)	**-0.0002 (-0.0003, -0.0002)**
p, 2df	*<0.001*	*<0.001*	*<0.001*
Frequency of public transport use(ref: every day or nearly every day)			
two or three times a week	-0.012 (-0.024, 0.0005)	-0.006 (-0.016, 0.004)	-0.003 (-0.016, 0.01)
once a week	**-0.020 (-0.034, -0.005)**	**-0.012 (-0.023, -0.001)**	-0.005 (-0.018, 0.009)
no use: no need	**-0.018 (-0.032, -0.003)**	**-0.012 (-0.022, -0.001)**	-0.004 (-0.017, 0.009)
no use: health problems	**-0.058 (-0.075, -0.04)**	**-0.088 (-0.102, -0.075)**	**-0.115 (-0.135, -0.094)**
no use: structural reasons	**-0.020 (-0.034, -0.005)**	-0.009 (-0.02, 0.002)	-0.0001 (-0.013, 0.013)
p, 5df	*<0.001*	*<0.001*	*<0.001*
Interaction between Age (linear term) and Frequency of public transport use			
Age*two or three times a week			-0.0004 (-0.002, 0.001)
Age*once a week			-0.001 (-0.002, 0.0003)
Age*no use: no need			**-0.001 (-0.003, -0.00001)**
Age*no use: health problems			0.002 (-0.00001, 0.003)
Age*no use: structural reasons			**-0.002 (-0.003, -0.0003)**
p, 5df			*<0.001*
**Random Part**			
Level 2 (Individual)			
Intercept variance		0.024	0.024
Age variance		0.00002	0.00002
Covariance of intercept and age		-0.0004	-0.0004
Level 1 (wave)			
Intercept		0.020	0.020
N observations (level 1)	27,525	27,525	27,525
N clusters (level 2)	9,659	9,659	9,659
Goodness of fit	Adj R-sq: 0.727	-2*LL: -13524.7	-2*LL: -13552.4

Boldface indicates statistical significance (p<0.05)

The results of the fixed effects model predicting grip strength are shown in online Supplementary file Table 3. Unsurprisingly, older adults who did not use public transport due to health problems had weaker grip strength than those who used public transport nearly/every day. However, there were no differences in grip strength between the latter group and those who did not use public transport due to structural reasons or because they did not need to.

For peer review only

Turning to the 1st growth curve model (Table 3, column 3), we see similar estimates for the intercept and age coefficients compared to the fixed effects model. All the coefficients for the frequency variable were also negative, although the negative effect of not using public transport due to structural reasons on walking speed was small and not different from those who used public transport nearly every day (the reference group). The second growth curve model adds in the interaction between the frequency and age. With increasing age, the effect on walking speed of not using public transport for structural reasons or because the respondent did not feel the need to, became increasingly more negative. The trajectories of these three groups (those who used public transport nearly every day, those who did not use because of structural reasons, and those who did not use because they did not need to) are shown in Figures 1 and 2. In both figures, the decline in walking speed with age started to diverge around age 75, when there was a slower decline in walking speed for those who used public transport nearly every day, and a much steeper decline for those who did not use public transport because of structural reasons, or a lack of need. The upper 95% confidence intervals of the latter two groups clearly did not overlap with the estimated trajectories of the frequent public transport users after about the age of 75.

[Fig.1 and Fig.2 here]

Discussion

This study found evidence that older adults living in England who frequently used public transport had faster walking speeds than their peers who did not use public transport. Results from fixed effects and multilevel growth curve models showed similar patterns. In fixed effects models, frequent public transport use among older adults had a protective effect on walking speed. Unsurprisingly, not using public transport due to health reasons had the largest negative effect on walking speed. However, not using public transport due to other reasons also had a negative effect on walking speed.

The results of the fixed effects models were corroborated by the trajectories of walking speed decline shown in the growth curve models. In the growth curve models, older adults who did not use public transport due to structural reasons or because of a lack of need ("through choice") had a faster decline in walking speed after the age of 75 than those who used public transport nearly every day.

The association between public transport use and muscle function deficits was specific to walking speed, and did not extend to another aging related muscle function deficit, grip strength. It is plausible that the frequent use of

For peer review only - http://bmjopen.bmj.com/site/about/guidelines.xhtml

public transport delays declines in muscles involved in walking, which in turn specifically impacts on walking speed and not other aging related muscle function declines. The specificity of the association also suggests that potential confounders related to strength, fitness, and health were unlikely to cause the public transport use-walking speed association.

Existing studies have found that use of public transport contributes to better health by increasing physical activity [10 11 16] and reducing obesity. [16 17 27] However these studies have not examined the reasons why people do not use public transport. Limiting health is potentially a key factor that could confound any association between public transport use and subsequent health. The use of repeated measurements of public transport use (and the underlying reasons for non-use) and walking speed from a large, representative sample of older adults has been useful in taking account of this key confounding factor. Respondents who "use public transport nearly every day" may be positively health selected. In the analyses, we take account of changes in health conditions in a number of ways. First we control for different health conditions (depression, mobility problems, ADL) that vary across waves. Secondly, respondents could select limiting health as the main reason why they could not use public transport- this is the main negative health selection group. Respondents who chose other reasons for not using public transport are thus less likely to be negatively health selected.

Limitations

Longitudinal attrition across waves may have resulted in a biased sample. We used the wave specific cross sectional survey weights to make the analyses representative of the older population but this may not adequately deal with attrition biases. Furthermore, there may be unobserved factors that cause the association between public transport use and walking speed. While we controlled for a number of factors in the analyses, the reported associations may still be biased.

Conclusion

It has become increasingly important for research to show a positive health impact from public transport use, especially among older adults, given cuts to public transport availability in England. [14] Although no cost-benefit analyses were undertaken in this study, savings to local government from cutting public transport may result in future increased expenditure on aging related conditions. Older adults who do not use public transport frequently are at risk of faster declines in their walking speed compared to those who use public transport every day. This

For peer review only - http://bmjopen.bmj.com/site/about/guidelines.xhtml

risk was evident not just among older adults who did not use public transport because of health problems, but also among those who did not use public transport because of structural barriers.

For peer review only

Acknowledgements

The data were made available through the UK Data Archive. ELSA was developed by a team of researchers based at the NatCen Social Research, University College London and the Institute for Fiscal Studies. The data were collected by NatCen Social Research. The funding is provided by the National Institute of Aging in the United States, and a consortium of UK government departments co-ordinated by the Office for National Statistics. The developers and funders of ELSA and the Archive do not bear any responsibility for the analyses or interpretations presented here.

PR participated in a Newton Fund/British Council/CONFAP Researcher Links Workshop, "Indicators for a Healthy City", in Belo Horizonte, Brazil, May 2016, which was coordinated by Professor Jennifer Mindell (UCL) and Professor Waleska Caiaffa (Federal University of Minas Gerais, UFMG).

Contributors: PR is responsible for study conception, study design, data analysis, and manuscript preparation and revision; had access to all of the data in the study; and takes responsibility for the integrity of the data and the accuracy of the data analysis. TC contributed to conception and design of the study, data analysis, data interpretation, manuscript preparation and revision. EW contributed to the design of the study, manuscript revision and provided input on the analysis of the study. All authors read and approved the final manuscript.

Funding: PR was supported by a grant from the Economic and Social Research Council [ES/P010075/1]. EW and TC were supported by a grant from the Economic and Social Research Council [ES/J019119/1]. TC was supported by a grant from the Medical Research Council [G1001375].

Disclaimer: The sponsors had no role in the study design; collection, analysis, and interpretation of the data; writing the report; or the decision to submit the report for publication.

Ethics approval: Ethical approval for all the ELSA waves was granted from NHS Research Ethics Committees under the National Research and Ethics Service (NRES). For further information see: http://www.nres.nhs.uk/.

Competing interest: None declared.

Data sharing statement: No additional data are available.

For peer review only

References

1. Cooper R, Kuh D, Hardy R. Objectively measured physical capability levels and mortality: systematic review and meta-analysis. *BMJ* 2010;341:c4467. doi: 10.1136/bmj.c4467 [published Online First: 2010/09/11]

2. Cooper R, Kuh D, Cooper C, et al. Objective measures of physical capability and subsequent health: a systematic review. *Age Ageing* 2011;40(1):14-23. doi: 10.1093/ageing/afq117 [published Online First: 2010/09/17]

3. Risser R, Lexell EM, Bell D, et al. Use of local public transport among people with cognitive impairments – A literature review. *Transportation Research Part F: Traffic Psychology and Behaviour* 2015;29:83-97. doi: http://dx.doi.org/10.1016/j.trf.2015.01.002

4. Schwanen T, Lucas K, Akyelken N, et al. Rethinking the links between social exclusion and transport disadvantage through the lens of social capital. *Transportation Research Part A: Policy and Practice* 2015;74:123-35. doi: http://dx.doi.org/10.1016/j.tra.2015.02.012

5. Green J, Jones A, Roberts H. More than A to B: the role of free bus travel for the mobility and wellbeing of older citizens in London. *Ageing and Society* 2012;34(3):472-94. doi: 10.1017/S0144686X12001110

6. Montero-Fernandez N, Serra-Rexach JA. Role of exercise on sarcopenia in the elderly. *Eur J Phys Rehabil Med* 2013;49(1):131-43. [published Online First: 2013/04/12]

7. Freiberger E, Sieber C, Pfeifer K. Physical activity, exercise, and sarcopenia - future challenges. *Wien Med Wochenschr* 2011;161(17-18):416-25. doi: 10.1007/s10354-011-0001-z [published Online First: 2011/07/28]

8. Samitz G, Egger M, Zwahlen M. Domains of physical activity and all-cause mortality: systematic review and dose-response meta-analysis of cohort studies. *Int J Epidemiol* 2011;40(5):1382-400. doi: 10.1093/ije/dyr112

9. Barr A, Bentley R, Simpson JA, et al. Associations of public transport accessibility with walking, obesity, metabolic syndrome and diabetes. *Journal of Transport & Health* 2016;3(2):141-53. doi: http://dx.doi.org/10.1016/j.jth.2016.01.006

10. Rissel C, Curac N, Greenaway M, et al. Physical Activity Associated with Public Transport Use—A Review and Modelling of Potential Benefits. *Int J Environ Res Public Health* 2012;9(7):2454-78. doi: 10.3390/ijerph9072454

11. Saelens BE, Vernez Moudon A, Kang B, et al. Relation Between Higher Physical Activity and Public Transit Use. *Am J Public Health* 2014;104(5):854-59. doi: 10.2105/AJPH.2013.301696

12. Mackett R. Improving accessibility for older people – Investing in a valuable asset. *Journal of Transport & Health* 2015;2(1):5-13. doi: http://dx.doi.org/10.1016/j.jth.2014.10.004

13. Mackett R. Overcoming the Barriers to Access for Older People. Report Produced for the Age Action Alliance 2014 [Available from: http://ageactionalliance.org/wordpress/wp-content/uploads/2014/11/Overcoming-the-barriers-to-access-Nov-14.pdf accessed 10 Nov 2016.

14. Department for Transport. Annual bus statistics: England 2015/16 2016 [Available from: https://www.gov.uk/government/uploads/system/uploads/attachment_data/file/561060/annual-bus-statistics-year-ending-march-2016.pdf accessed 10 Nov 2016.

15. Holley-Moore G, Creighton H. The Future of Transport in an Ageing Society London: ILC-UK; 2015 [updated November 2016 Available from: http://www.ilcuk.org.uk/index.php/publications/publication_details/the_future_of_transport_in_an_ageing_society accessed 10 Nov 2016.

For peer review only

16. Webb E, Laverty A, Mindell J, et al. Free Bus Travel and Physical Activity, Gait Speed, and Adiposity in the English Longitudinal Study of Ageing. *Am J Public Health* 2016;106(1):136-42. doi: 10.2105/AJPH.2015.302907

17. Webb E, Netuveli G, Millett C. Free bus passes, use of public transport and obesity among older people in England. *J Epidemiol Community Health* 2012;66(2):176-80. doi: 10.1136/jech.2011.133165

18. Coronini-Cronberg S, Millett C, Laverty AA, et al. The impact of a free older persons' bus pass on active travel and regular walking in England. *Am J Public Health* 2012;102(11):2141-8. doi: 10.2105/ajph.2012.300946 [published Online First: 2012/09/22]

19. Weber D. Differences in physical aging measured by walking speed: evidence from the English Longitudinal Study of Ageing. *BMC Geriatr* 2016;16:31. doi: 10.1186/s12877-016-0201-x [published Online First: 2016/01/30]

20. Zaninotto P, Sacker A, Head J. Relationship between wealth and age trajectories of walking speed among older adults: evidence from the English Longitudinal Study of Ageing. *J Gerontol A Biol Sci Med Sci* 2013;68(12):1525-31. doi: 10.1093/gerona/glt058 [published Online First: 2013/05/18]

21. Broome K, McKenna K, Fleming J, et al. Bus use and older people: A literature review applying the Person–Environment–Occupation model in macro practice. *Scand J Occup Ther* 2009;16(1):3-12. doi: 10.1080/11038120802326222

22. Broome K, Nalder E, Worrall L, et al. Age-friendly buses? A comparison of reported barriers and facilitators to bus use for younger and older adults. *Australasian journal on ageing* 2010;29(1):33-8. doi: 10.1111/j.1741-6612.2009.00382.x [published Online First: 2010/04/20]

23. Steptoe A, Breeze E, Banks J, et al. Cohort Profile: The English Longitudinal Study of Ageing. *Int J Epidemiol* 2013,6(42):1640-09. doi:10.1093/ije/dys168.

24. Katz S, Ford AB, Moskowitz RW, et al. Studies of illness in the aged. The index of ADL: A standardized measure of biological and psychological function. *JAMA. 1963;185(12):914-919. doi:10.1001/jama.1963.03060120024016.*

25. Radloff LS. The CES-D Scale: A Self-Report Depression Scale for Research in the General Population. *Appl Psychol Meas* 1977;1(3):385-401. doi: 10.1177/014662167700100306

26. Demakakos P, Hamer M, Stamatakis E, et al. Low-intensity physical activity is associated with reduced risk of incident type 2 diabetes in older adults: evidence from the English Longitudinal Study of Ageing. *Diabetologia* 2010;53(9):1877-85. doi: 10.1007/s00125-010-1785-x [published Online First: 2010/05/25]

27. Flint E, Webb E, Cummins S. Change in commute mode and body-mass index: prospective, longitudinal evidence from UK Biobank. *The Lancet Public Health* 2016 doi: 10.1016/S2468-2667(16)30006-8

For peer review only

Figure Captions

Figure 1

Predicted decline in walking speed with age by public transport use, comparing ELSA respondents who use public transport every day and those who do not use public transport because of structural reasons

Figure 2

Predicted decline in walking speed with age by public transport use, comparing ELSA respondents who use public transport every day and those who do not use public transport because they do not need to

For peer review only

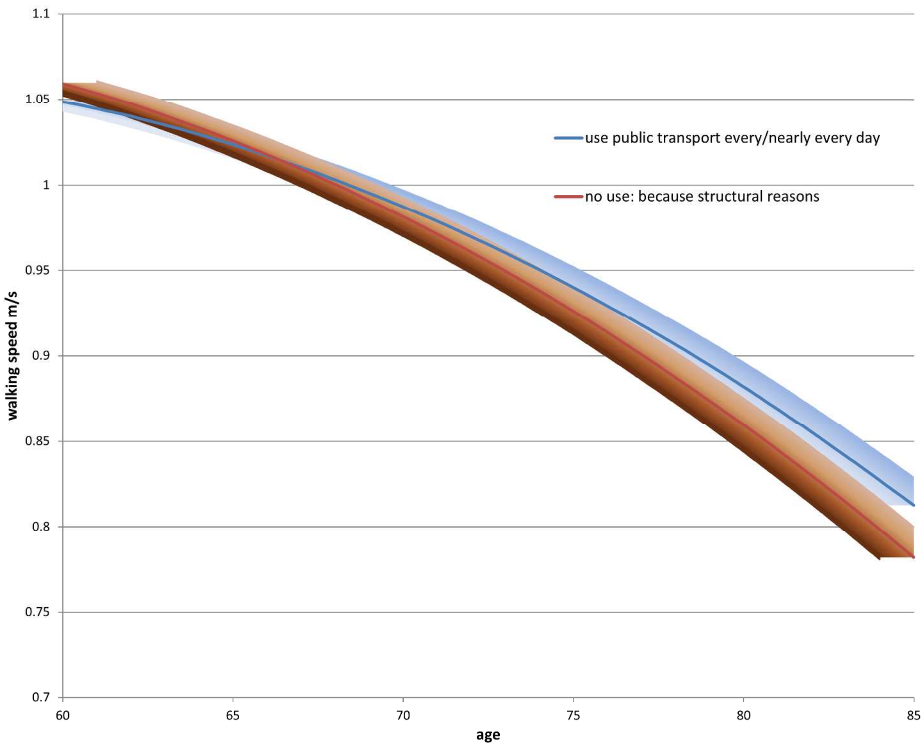

Predicted decline in walking speed with age by public transport use, comparing ELSA respondents who use public transport every day and those who do not use public transport because of structural reasons

254x190mm (300 x 300 DPI)

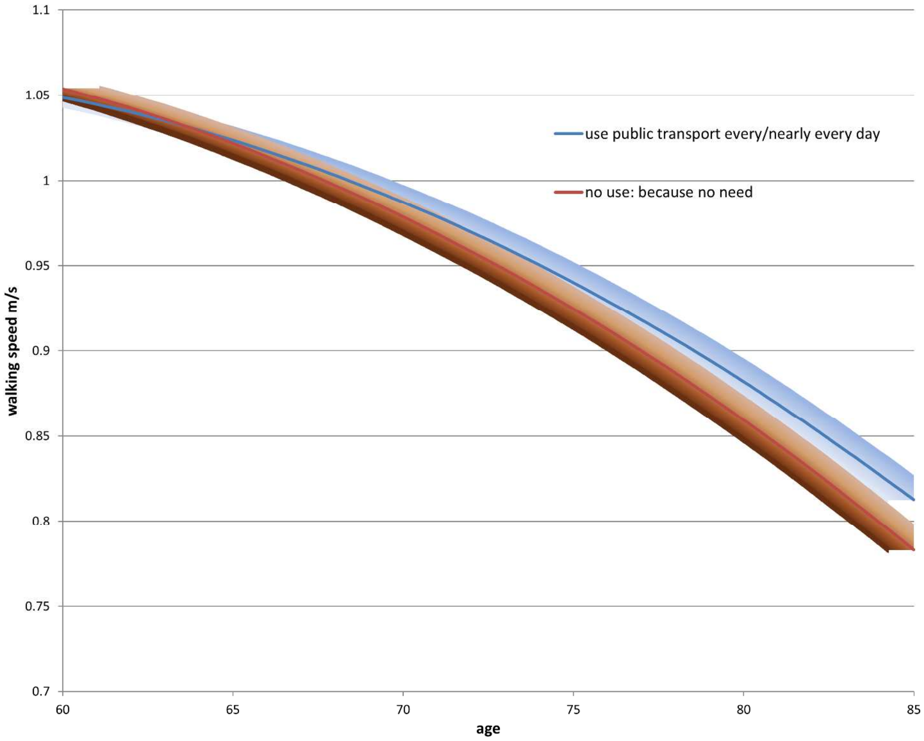

Predicted decline in walking speed with age by public transport use, comparing ELSA respondents who use public transport every day and those who do not use public transport because they do not need to

254x190mm (300 x 300 DPI)

For peer review only - http://bmjopen.bmj.com/site/about/guidelines.xhtml

Frequent public transport use reduces age-related decline in walking speed among older adults

Patrick Rouxel, PhD[1], Elizabeth Webb, PhD [2] and Tarani Chandola PhD [3]

[1]CLOSER, Department of Social Science, University College London Institute of Education, London, UK, ORCID: 0000-0003-0330-554X, [2] International Centre for Lifecourse Studies, Department of Epidemiology and Public Health, University College London, London, UK, ORCID: 0000-0001-6106-5367, and [3]CMIST and Social Statistics, University of Manchester, UK, ORCID: 0000-0002-1864-3413

To the Journal of Epidemiology and Community Health

Corresponding author

Patrick Rouxel, PhD, CLOSER, Department of Social Science, University College London Institute of Education, 55-59 Gordon Square, WC1H 0NU, London, UK. E-mail: patrick.rouxel@ucl.ac.uk

For peer review only

Supplementary Table 1 Coefficients (95% CI) from the Fixed Effects Model of walking speed (m/s), ELSA waves 2-6

**Fixed Part**	Coefficients	Lower 95% CI	Upper 95% CI
Intercept	**0.879**	0.831	0.926
Age centered (linear term)	**-0.008**	-0.010	-0.007
Age (quadratic term)	**-0.0003**	-0.0004	-0.0003
Public transport use(ref: every/nearly every day)			
two or three times a week	-0.012	-0.024	0.0005
once a week	**-0.020**	-0.034	-0.005
did not use because no need	**-0.018**	-0.032	-0.003
did not use because health problems	**-0.058**	-0.075	-0.040
did not use because structural reasons	**-0.020**	-0.034	-0.005
Wealth quintiles (ref: poorest quintile)			
Quintile 2	0.008	-0.010	0.025
Quintile 3	0.009	-0.011	0.028
Quintile 4	0.017	-0.004	0.037
Richest quintile	**0.026**	0.004	0.049
Access to car/van (ref: yes access)			
No access to car/van	**-0.011**	-0.022	-0.0003
Employment status (ref: employed)			
Retired	0.002	-0.011	0.014
Other	0.001	-0.014	0.016
Social class (ref: Managerial & Prof)			
Intermediate occupations	-0.020	-0.058	0.019
Self-employed	0.009	-0.031	0.049
Lower supervisory & technical	-0.015	-0.064	0.034
Semi-routine & routine	0.019	-0.017	0.056
Urban/Rural (ref: Urban)			
Town & Fringe	0.017	-0.025	0.058
Village	0.014	-0.027	0.056
Hamlet/Isolated	0.028	-0.026	0.082
Mobility difficulties (ref: none)			
1-3 difficulties	**-0.013**	-0.020	-0.005
4+ difficulties	**-0.052**	-0.063	-0.041
Activities of Daily living impairments (ref: none)			
At least one ADL impairment	**-0.032**	-0.040	-0.024
Physical Activity levels (ref: sedentary)			
Low	**0.030**	0.017	0.043
Moderate	**0.054**	0.040	0.068
High	**0.065**	0.049	0.081
Marital status (ref: married)			
Separated/Divorced	**0.038**	0.007	0.069
Widowed	0.010	-0.022	0.041
Never married	0.015	-0.036	0.066
Cohabitation (ref: not living with partner)			
Living with partner	0.013	-0.019	0.046
CESD- Depression score	**-0.007**	-0.009	-0.005
Smoking status (ref: never smoker)			
Ex-Smoker	0.003	-0.020	0.027
Current smoker	0.010	-0.019	0.039
Number of observations	27,525		
Number of clusters	9,659		
Adjusted R-squared	0.727		

Boldface indicates statistical significance ($p<0.05$)

For peer review only

For peer review only - http://bmjopen.bmj.com/site/about/guidelines.xhtml

Supplementary Table 2 Coefficients (95% CI) from the Multilevel Growth Curve Model of walking speed (m/s), ELSA waves 2-6

**Fixed Part**	Coefficients	Lower 95% CI	Upper 95% CI
Intercept	**0.863**	0.835	0.892
Age centered (linear term)	**-0.006**	-0.008	-0.005
Age (quadratic term)	**-0.0002**	-0.0003	-0.0002
Public transport use(ref: every/nearly every day)			
two or three times a week	-0.003	-0.016	0.010
once a week	-0.005	-0.018	0.009
did not use because no need	-0.004	-0.017	0.009
did not use because health problems	**-0.115**	-0.135	-0.094
did not use because structural reasons	-0.0001	-0.013	0.013
Interaction between Age and Public transport use			
Age*two or three times a week	0.000	-0.002	0.001
Age*once a week	-0.001	-0.002	0.0003
Age*did not use because no need	**-0.001**	-0.003	-0.00001
Age*did not use because health problems	0.002	0.0000	0.003
Age*did not use because structural reasons	**-0.002**	-0.003	-0.0003
Sex (ref: men)			
Women	**-0.020**	-0.028	-0.012
Wealth quintiles (ref: poorest quintile)			
Quintile 2	**0.026**	0.016	0.036
Quintile 3	**0.043**	0.033	0.054
Quintile 4	**0.066**	0.055	0.077
Richest quintile	**0.094**	0.082	0.105
Access to car/van (ref: yes access)			
No access to car/van	**-0.027**	-0.035	-0.019
Employment status (ref: employed)			
Retired	**-0.010**	-0.019	-0.0005
Other	**-0.019**	-0.031	-0.008
Social class (ref: Managerial & Prof)			
Intermediate occupations	**-0.020**	-0.031	-0.008
Self-employed	**-0.022**	-0.034	-0.010
Lower supervisory & technical	**-0.055**	-0.068	-0.043
Semi-routine & routine	**-0.051**	-0.061	-0.042
Urban/Rural (ref: Urban)			
Town & Fringe	**0.015**	0.004	0.026
Village	**0.019**	0.008	0.031
Hamlet/Isolated	0.017	-0.001	0.036
Mobility difficulties (ref: none)			
1-3 difficulties	**-0.037**	-0.043	-0.031
4+ difficulties	**-0.121**	-0.130	-0.113
Activities of Daily living impairments (ref: none)			
At least one ADL impairment	**-0.055**	-0.062	-0.048

For peer review only

Supplementary Table 2 continued, Coefficients (95% CI) from the Multilevel Growth Curve Model of walking speed (m/s), ELSA waves 2-6

Physical Activity levels (ref: sedentary)			
Low	**0.052**	0.040	0.063
Moderate	**0.099**	0.087	0.111
High	**0.122**	0.109	0.135
Marital status (ref: married)			
Separated/Divorced	0.011	-0.008	0.031
Widowed	0.009	-0.011	0.030
Never married	-0.015	-0.039	0.009
Cohabitation (ref: not living with partner)			
Living with partner	0.011	-0.008	0.031
CESD- Depression score	**-0.011**	-0.012	-0.009
Smoking status (ref: never smoker)			
Ex-Smoker	-0.006	-0.014	0.001
Current smoker	**-0.028**	-0.039	-0.016
**Random Part**			
Level 2 (Individual)			
Intercept variance			0.024
Age centered (linear term) variance			0.00002
Covariance of intercept and age centered			-0.0004
Level 1 (wave)			
Intercept			0.020
Number of observations (level 1)			27,525
Number of clusters (level 2)			9,659
-2*Log Likelihood			-13552.4

Boldface indicates statistical significance ($p<0.05$)

For peer review only

For peer review only - http://bmjopen.bmj.com/site/about/guidelines.xhtml

Supplementary Table 3 Coefficients (95% CI) from the Fixed Effects Model of grip strength (kg), ELSA waves 2-6

**Fixed Part**	Coefficients	Lower 95% CI	Upper 95% CI
Intercept	**28.01**	26.48	29.53
Age centered (linear term)	**-0.41**	-0.44	-0.38
Age (quadratic term)	0.00	-0.01	0.00
Public transport use(ref: every/nearly every day)			
two or three times a week	-0.18	-0.64	0.28
once a week	-0.18	-0.70	0.34
did not use because no need	-0.07	-0.60	0.46
did not use because health problems	**-0.80**	-1.49	-0.11
did not use because structural reasons	-0.22	-0.74	0.31
Wealth quintiles (ref: poorest quintile)			
Quintile 2	-0.40	-1.06	0.26
Quintile 3	-0.31	-1.02	0.39
Quintile 4	-0.21	-0.96	0.55
Richest quintile	-0.06	-0.87	0.75
Access to car/van (ref: yes access)			
No access to car/van	0.14	-0.31	0.58
Employment status (ref: employed)			
Retired	0.01	-0.36	0.38
Other	**-0.52**	-1.01	-0.04
Social class (ref: Managerial & Prof)			
Intermediate occupations	0.04	-0.86	0.93
Self-employed	-0.57	-1.55	0.42
Lower supervisory & technical	-0.40	-1.51	0.71
Semi-routine & routine	-0.03	-0.93	0.86
Urban/Rural (ref: Urban)			
Town & Fringe	-0.01	-1.00	0.99
Village	-0.26	-1.41	0.90
Hamlet/Isolated	1.03	-0.41	2.47
Mobility difficulties (ref: none)			
1-3 difficulties	-0.15	-0.42	0.11
4+ difficulties	**-0.85**	-1.28	-0.41
Activities of Daily living impairments (ref: none)			
At least one ADL impairment	**-0.64**	-0.99	-0.29
Physical Activity levels (ref: sedentary)			
Low	**0.79**	0.20	1.39
Moderate	**0.89**	0.27	1.50
High	**1.08**	0.41	1.75
Marital status (ref: married)			
Separated/Divorced	-0.66	-1.72	0.41
Widowed	-0.52	-1.58	0.53
Never married	-1.10	-3.27	1.07
Cohabitation (ref: not living with partner)			
Living with partner	-0.57	-1.63	0.50
CESD- Depression score	-0.07	-0.14	0.00

For peer review only

For peer review only - http://bmjopen.bmj.com/site/about/guidelines.xhtml

Supplementary Table 3 continued, Coefficients (95% CI) from the Fixed Effects Model of grip strength, ELSA waves 2-6

Smoking status (ref: never smoker)			
Ex-Smoker	0.43	-0.31	1.17
Current smoker	0.59	-0.43	1.61
Number of observations	21,799		
Adjusted R-squared	0.8721		

Boldface indicates statistical significance ($p<0.05$)

For peer review only

STROBE 2007 (v4) Statement—Checklist of items that should be included in reports of *cohort studies*

Section/Topic	Item #	Recommendation	Reported on page #
**Title and abstract**	1	(*a*) Indicate the study's design with a commonly used term in the title or the abstract	2
		(*b*) Provide in the abstract an informative and balanced summary of what was done and what was found	2
**Introduction**			
Background/rationale	2	Explain the scientific background and rationale for the investigation being reported	4-5
Objectives	3	State specific objectives, including any prespecified hypotheses	5
**Methods**			
Study design	4	Present key elements of study design early in the paper	5
Setting	5	Describe the setting, locations, and relevant dates, including periods of recruitment, exposure, follow-up, and data collection	5-6
Participants	6	(*a*) Give the eligibility criteria, and the sources and methods of selection of participants. Describe methods of follow-up	5-6
		(*b*) For matched studies, give matching criteria and number of exposed and unexposed	NA
Variables	7	Clearly define all outcomes, exposures, predictors, potential confounders, and effect modifiers. Give diagnostic criteria, if applicable	6-7
Data sources/ measurement	8*	For each variable of interest, give sources of data and details of methods of assessment (measurement). Describe comparability of assessment methods if there is more than one group	6-7
Bias	9	Describe any efforts to address potential sources of bias	7-8
Study size	10	Explain how the study size was arrived at	7
Quantitative variables	11	Explain how quantitative variables were handled in the analyses. If applicable, describe which groupings were chosen and why	6-7
Statistical methods	12	(*a*) Describe all statistical methods, including those used to control for confounding	7
		(*b*) Describe any methods used to examine subgroups and interactions	7
		(*c*) Explain how missing data were addressed	7-8
		(*d*) If applicable, explain how loss to follow-up was addressed	7-8
		(*e*) Describe any sensitivity analyses	NA
**Results**			

For peer review only

Participants	13*	(a) Report numbers of individuals at each stage of study—eg numbers potentially eligible, examined for eligibility, confirmed eligible, included in the study, completing follow-up, and analysed	7
		(b) Give reasons for non-participation at each stage	7-8
		(c) Consider use of a flow diagram	NA
Descriptive data	14*	(a) Give characteristics of study participants (eg demographic, clinical, social) and information on exposures and potential confounders	8-9
		(b) Indicate number of participants with missing data for each variable of interest	9-10
		(c) Summarise follow-up time (eg, average and total amount)	10
Outcome data	15*	Report numbers of outcome events or summary measures over time	
Main results	16	(*a*) Give unadjusted estimates and, if applicable, confounder-adjusted estimates and their precision (eg, 95% confidence interval). Make clear which confounders were adjusted for and why they were included	11
		(*b*) Report category boundaries when continuous variables were categorized	11
		(*c*) If relevant, consider translating estimates of relative risk into absolute risk for a meaningful time period	10
Other analyses	17	Report other analyses done—eg analyses of subgroups and interactions, and sensitivity analyses	Supplementary materials
**Discussion**			
Key results	18	Summarise key results with reference to study objectives	12
**Limitations**			
Interpretation	20	Give a cautious overall interpretation of results considering objectives, limitations, multiplicity of analyses, results from similar studies, and other relevant evidence	13
Generalisability	21	Discuss the generalisability (external validity) of the study results	13
**Other information**			
Funding	22	Give the source of funding and the role of the funders for the present study and, if applicable, for the original study on which the present article is based	15

*Give information separately for cases and controls in case-control studies and, if applicable, for exposed and unexposed groups in cohort and cross-sectional studies.

Note: An Explanation and Elaboration article discusses each checklist item and gives methodological background and published examples of transparent reporting. The STROBE checklist is best used in conjunction with this article (freely available on the Web sites of PLoS Medicine at http://www.plosmedicine.org/, Annals of Internal Medicine at http://www.annals.org/, and Epidemiology at http://www.epidem.com/). Information on the STROBE Initiative is available at www.strobe-statement.org.

For peer review only

BMJ Open

Does public transport use prevent declines in walking speed among older adults living in England? A prospective cohort study

Journal:	*BMJ Open*
Manuscript ID	bmjopen-2017-017702.R1
Article Type:	Research
Date Submitted by the Author:	04-Jul-2017
Complete List of Authors:	Rouxel, Patrick; University College London Institute of Education, Social Science<br>Webb, Elizabeth; University College London, International Centre for Lifecourse Studies, Epidemiology and Public Health<br>Chandola, Tarani; University of Manchester, The Cathie Mash Centre for Census and Survey Research
**Primary Subject Heading**:	Epidemiology
Secondary Subject Heading:	Public health
Keywords:	public transport, ageing, walking speed, physical activity

SCHOLARONE™ Manuscripts

For peer review only

Does public transport use prevent declines in walking speed among older adults living in England? A prospective cohort study

Patrick Rouxel [1], Elizabeth Webb [2] and Tarani Chandola [3]

[1]CLOSER, Department of Social Science, University College London Institute of Education, London, UK, ORCID: 0000-0003-0330-554X, [2] International Centre for Lifecourse Studies, Department of Epidemiology and Public Health, University College London, London, UK, ORCID: 0000-0001-6106-5367 and [3]CMIST and Social Statistics, University of Manchester, UK, ORCID: 0000-0002-1864-3413

Corresponding author

Patrick Rouxel, CLOSER, Department of Social Science, University College London Institute of Education, 55-59 Gordon Square, WC1H 0NU, London, UK. E-mail: patrick.rouxel@ucl.ac.uk; phone: +44 7527565347

Word count 4,113

For peer review only

Abstract

Objectives: Although there is some evidence that public transport use confers public health benefits, the evidence is limited by cross-sectional study designs and health-related confounding factors. This study examines the effect of public transport use on changes in walking speed among older adults living in England, comparing frequent users of public transport to their peers who did not use public transport because of structural barriers (poor public transport infrastructure), or through choice.

Design: Prospective cohort study.

Setting: England, UK

Participants: Older adults aged 60 or older eligible for the walking speed test. 6,246 individuals at wave 2 (2004-05); 5,909 individuals at wave 3 (2006-07); 7,321 individuals at wave 4 (2008-09); 7,535 individuals at wave 5 (2010-11); and 7,664 individuals at wave 6 (2012-13) of the English Longitudinal Study of Ageing.

Main outcome measure: The walking speed was estimated from the time taken to walk 2.4 metres. Fixed effects models and growth curve models were used to examine the associations between public transport use and walking speed.

Results: Older adults who did not use public transport through choice or because of structural reasons had slower walking speeds [-0.02 m/s (95%CI -0.03,-0.003) and -0.02 m/s (95%CI -0.03,-0.01), respectively] and took an extra 0.07 seconds to walk 2.4 meters compared to their peers who used public transport frequently. The age-related trajectories of decline in walking speed were slower for frequent users of public transport compared to non-users.

Conclusions: Frequent use of public transport may prevent age-related decline in physical capability by promoting physical activity and lower limb muscle strength among older adults. The association between public transport use and slower decline in walking speed among older adults is unlikely to be confounded by health related selection factors. Improving access to good quality public transport could improve the health of older adults.

Strengths and limitations of the study

- Previous cross-sectional research on the protective role of public transport use in relation to age related functional declines may have been biased by health selection processes as people age.
- Older adults with deteriorating health are less likely to use public transport and their poor health could determine their functional decline, rather than their lack of public transport use.

For peer review only

- This longitudinal study of over 7,000 adults living in England suggests that the inference that frequent use of public transport may prevent age-related decline in physical capability is robust to such potential biases.
- While this is not a causal analysis, we have controlled for within- and between-person factors that could bias the association between public transport use and walking speed decline.
- Given the current context of cuts in public transport availability in England, this research suggests that such cuts may result in faster declines in physical functioning as people age.

For peer review only

Introduction

Declines in walking speed and grip strength are markers of ageing and are associated with all-cause mortality [1] and poorer health in older populations. [2] Maintaining physical capability is a prerequisite for older people to engage independently in many social activities [3] and for reducing social exclusion. [4 5]

Functional capacity and muscle strength are key dimensions of sarcopenia. [6] Compelling evidence supports the efficacy of physical activity in maintaining muscle mass, strength and function in older adults. [7 8] Older adults may add regular physical activity into daily life by walking and maintaining balance on moving vehicles such as buses or trains. [7] Public transport related physical activity was associated with a larger reduction in mortality for older adults (above 70 years) compared to younger adults. [9] Public transport users are more physically active than non-users of public transport. [10-12]

Disability is not the only barrier to the use of public transport for many older people. Other barriers include the costs and poor quality of public transport. [5 13 14] These barriers, in turn, suppress leisure, social interactions and shopping activities. [13 15] Accessible, affordable and convenient transport is important to enable older people to access services and amenities. It is particularly important to consider such barriers to public transport use given the current context of cuts to local bus services in England. [16]

Despite concessionary bus passes in the UK offering free bus travel to those over the State Pension Age, in England a third of older adults aged over 65 never use public transport, whilst another third use it very infrequently. [17] Evidence from the English Longitudinal Study of Ageing (ELSA) [18 19] and other studies [5 20] showed that free bus travel for older people was associated with increased active travel and raised physical activity. A review has reported between 8 and 33 minutes of additional physical activity by walking through use of public transport, although most of the studies included were not focused on older adults. [11] Although walking speed declines with age, [21 22] there is some cross-sectional evidence that older women with a free bus pass use public transport more often and have faster walking speeds than those who do not hold a bus pass. [18] Moreover, public transport use is associated with lower levels of obesity and may have a protective effect against becoming obese. [19] A recent longitudinal study on the use of active or public transport versus cars to commute to work showed that people who changed from active or public commuting to car commuting had an increase in BMI of

For peer review only - http://bmjopen.bmj.com/site/about/guidelines.xhtml

0.3 kg/m^2 while those who changed from car commuting to active or public commuting had a decrease in BMI of 0.3 kg/m^2. [23]

However, the key limitation in the existing evidence is that negative health selection is not taken into account. Older adults with deteriorating health may be less likely to use public transport and their poor health could determine the decline in walking speed, rather than their lack of public transport use. There is also a lack of analyses on different reasons for not using public transport. Older adults may not use public transport because of health problems and disabilities [3 24] or because reliable public transport is not available, [13] or because they prefer using their own vehicles. [25] Separating out these reasons for not using public transport is important: if a negative effect of not using public transport on walking speed is observed among older adults who do not use public transport due to structural reasons (poor public transport infrastructure) or through choice, this suggests the observed association between public transport use and walking speed is not confounded by health.

An additional test of the specificity of the public transport-walking speed association is whether similar associations are observed between public transport use and upper body strength as measured by grip strength. As public transport use is unlikely to have an impact on upper body strength, any association between public transport use and grip strength is likely to be caused by confounding factors such as stronger people being selected into using public transport. Moreover, if public transport use affects walking speed, the mechanisms are likely to be through walking related physical activity and slower declines in lower limb strength.

Our study will address the following research questions:

RQ1: Do older adults who frequently use public transport have faster walking speeds than those who do not use public transport for structural reasons or through choice? Is some of the association between public transport use and walking speed going through the mechanisms of physical activity and lower limb strength?

RQ2: Is the association of public transport use with muscle function deficit specific to lower limb muscle strength? Does public transport use also predict stronger grip strength?

RQ3: Are declines of walking speed with age slower for older adults who use public transport often, compared to their peers who do not use public transport for structural reasons or through choice?

Methods

For peer review only - http://bmjopen.bmj.com/site/about/guidelines.xhtml

Data

The data come from waves 2 to 6 of the English Longitudinal Study of Ageing (ELSA), where individuals aged 50 and over living in private households in England were followed and re-interviewed every two years. [26] The ELSA sample was refreshed at waves 3, 4, and 6 to ensure the sample remained representative of the population aged 50 and older. The National Research Ethics Service approved the study, and all participants gave their informed consent. We used data from older adults aged 60 or older - those who were eligible for the walking speed test - consisting of 6,246 individuals at wave 2 (2004-05); 5,909 individuals at wave 3 (2006-07); 7,321 individuals at wave 4 (2008-09); 7,535 individuals at wave 5 (2010-11); and 7,664 individuals at wave 6 (2012-13). Data from wave 1 were omitted, since the reasons why people did not use public transport were not asked. Data were collected though face-to-face interview and a self-completion questionnaire. In addition, there was a nurse visit at waves 2, 4 and 6. The ELSA data and documentation are publicly available from the UK Data Service. (http://www.esds.ac.uk/findingData/snDescription.asp?sn=5050).

Variables

Walking speed (m/s) was measured among participants aged 60 and older at every ELSA wave. They were asked to twice walk a distance of 8 feet (2.4m) at their usual pace. Walking aids were permitted. We used the mean walking speed based on the two timings.

Grip strength (kg) was used as an indicator of upper body strength. Participants were asked to squeeze a hand-held dynamometer up to three times with each hand in waves 2, 4 and 6. We used the mean of the three measurements for the dominant hand

Chair stands were used as an indicator of lower limb muscle strength. Respondents at waves 2, 4 and 6 aged under 70 were asked to do 10 chair stands and those aged 70+ were asked to do 5 chair stands. This was grouped into people who could not complete either 5 or 10 chair stands (including those who could not complete a single chair stand), those who took longer than the median time to complete 5/10 chair stands, and those who completed the task in less than the median time.

Participants were asked how often they used public transport. In addition, those who rarely or never used public transport were asked to provide the reasons of not using public transport more often. We then derived the

For peer review only - http://bmjopen.bmj.com/site/about/guidelines.xhtml

frequency variable as follows: every day/nearly every day; 2-3 times a week; once a week; no use because did not need to (or in other words through "choice"); no use because of health problems; no use because of structural reasons. Structural reasons, for these purposes, include: not convenient, does not go where they want, infrequent, unreliable, too expensive, too dirty, fear of crime. At wave 2, the frequency of use variable had different responses which we mapped onto the later wave responses as follows: a lot=nearly every day; quite often=2-3 times a week; sometimes=once a week; rarely/never=no use. [19]

Covariates

A quadratic term for continuous age was specified to characterize non-linear age effects.

Other covariates included gender; marital status (married; divorced/separated; widowed; never married), cohabiting status (currently living with a partner or not); urban/rural areas (urban; town; village; hamlet) - the rural–urban definition is applied to the Census Output Area that each individual lives in; quintiles of non-pension wealth; access to car (whether driver or passenger); employment status (employed; retired; other); National Statistics Socio-economic Classification (NS-SEC) social class; smoking (never; former; current). Mobility difficulties were assessed by asking participants whether they had difficulties with 10 common functions (e.g. walking 100 yards; climbing several flights of stairs without resting; climbing one flight of stairs without resting; lifting or carrying weights). We derived a variable with three categories: no difficulties; 1-3 difficulties, 4 or more difficulties. The number of functional limitations in Activities of Daily Living (ADLs) provides an indication of disability. The ADLs scale [27] comprises problems with dressing, walking, bathing, eating (such as cutting up food), getting out of bed, and using the toilet. We derived a binary variable no limitations in ADLs; at least one limitation in ADLs. Depressive symptoms were measured using the eight-item version of the Center for Epidemiologic Study Depression (CES-D) scale. [28] Participants were asked how often they participated in mild, moderate or vigorous physical activity including participation in occupations that involve physical work. Based on their responses, they were classified into the following categories: sedentary; low; moderate or high level of physical activity. [29] Cognitive function was assessed through two memory tests. Study participants were asked to recall a list of 10 words immediately after reading them and then again after a 5-minute delay. We computed an overall memory score (range, 0– 20) using both the immediate and delayed recall results (between-test correlation coefficient = 0.70). Orientation in time (day, month, year and day of the week) is another test of memory.

For peer review only - http://bmjopen.bmj.com/site/about/guidelines.xhtml

Analysis

For RQs 1 and 2: Fixed effects linear and multinomial regression models were used to regress walking speed, physical activity, chair stands and grip strength on the frequency of using public transport. These models investigate the effects of within individual (time varying) changes in public transport use on changes in walking speed, physical activity, lower limb and upper body muscle strength, taking into account other time varying covariates. We also used fixed effects multinomial logit models to examine whether the direction of association could be in the opposite direction, i.e. whether changes in the use/non-use of public transport (for different reasons) were predicted by changes in walking speed and other covariates. We estimated these models in Stata 14.0.

For RQ3: We used multilevel (random effects) growth curve models to estimate age-related trajectories of walking speed for different categories of public transport use. These models can be used to describe how different trajectories of walking speed change with age, by interacting age with the frequency of public transport use. Individual trajectories (at level 2) of walking speed (at level 1) are estimated, with a random slope of age (at level 2) characterizing differences in individual trajectories of walking speed. As participants with a single walking speed measurement can contribute can contribute to the overall growth curve model, we additionally estimated age-related trajectories of walking speed for those participants with at least three waves of walking speed measurements. We estimated these models in MLwiN 2.1.

Missing data

For the walking speed analyses, there are 29,894 observations of walking speed between waves 2 to 6, which reduced to 27,525 observations in the statistical models due to missing data in the covariates. Attrition between waves was not strictly monotonic- some ELSA participants returned to the study after missing one or two waves of data collection. Hence rather than analysing factors only associated with attrition, we analysed factors associated with missing data in any of the independent and dependent variables and covariates, conditional on observation of a participant’s walking speed at baseline.

Analyses of the pattern of missingness in the cohort with a baseline walking speed measurement revealed that 33% of that cohort had subsequently dropped out by wave 6, 16% were missing a walking speed measure at

For peer review only

wave 6, 5% were missing both a walking speed and wealth measurement, and 3% were missing a wealth measurement. Other covariates accounted for less than 2% of the missing data.

We modelled the odds of having any missing data (conditional on having a baseline walking speed measurement) as shown in Supplementary Table S1. Women and older participants were less likely to have any missing data, especially older women participants. Participants who did not use public transport because of health problems were more likely to be missing compared to those who used public transport frequently, but those who did not use public transport for other reasons were not more likely to be missing. Socio-economic disadvantage (not having access to a car/van and being in the semi-routine and routine occupational class) was associated with higher odds of being missing, as was having a disability, low physical activity, low memory scores and higher levels of depressive symptoms.

The ELSA study team provides longitudinal weights for the core ELSA members present at each wave from the first wave. We did not use these for our analyses as, since they are not available for the ELSA refreshment sample members, using the longitudinal weights would have reduced our sample size by more than half. Instead, we used the wave specific cross-sectional weights in both the fixed effects and multilevel growth curve models, in order to make our analyses representative of non-institutionalized older adults living in England. These cross-sectional weights take into account the greater likelihood of non-response by participants who have poorer health and who are more socio-economically disadvantaged.[30]

Results

Table 1 shows the distribution of all the variables used in the analysis. The mean walking speed was 0.85 m/s and the mean age was 70.5. 34% of ELSA respondents across waves 2-6 reported taking public transport at least once a week, while 33% reported not using public transport because of structural reasons. Most of the sample (73%) lived in urban areas, and almost 60% reported at least one difficulty with mobility.

For peer review only - http://bmjopen.bmj.com/site/about/guidelines.xhtml

Table 1 Distribution (percentage/mean) of all the variables in the analysis, observations (n) across ELSA waves 2-6 among respondents with walking speed data

Variables	%/Mean (sd)	n obs across 6 waves	Variables	%/Mean (sd)	n obs across 6 waves
**Walking speed (m/s)**	0.9 (0.3)	29894	**Urban/rural**		
**Chair stands**			urban	72.6%	21,679
could not complete test	17.5%	2780	town	12.6%	3,771
completed test slower	45.8%	7288	village	10.8%	3,213
completed test faster	36.8%	5857	hamlet	4.0%	1,198
**Frequency of public transport use**			**Marital status**		
every day or nearly every day	8.5%	2,540	married	65.0%	19,429
two or three times a week	14.4%	4,300	separated/divorced	9.8%	2,923
once a week	11.2%	3,342	widowed	20.5%	6,117
no use: no need	27.9%	8,337	never married	4.8%	1,421
no use: health problems	5.5%	1,631	**Mobility difficulties**		
no use: structural reasons	32.6%	9,741	none	40.7%	12,155
**Age**	70.5 (7.7)	29,894	1 to 3	37.6%	11,233
**Gender**			4 or more	21.8%	6,502
male	45.9%	13,728	**Functional status (ADLS)**		
female	54.1%	16,166	no limitation	82.1%	24,538
**Wealth quintiles**			at least 1 limitation	17.9%	5,355
poorest	16.4%	4,660	**CES-D depression score**	1.3 (1.8)	29579
2nd	18.8%	5,327	**Physical activity**		
3rd	21.2%	5,999	sedentary	5.0%	1,503
4th	21.6%	6,135	low	26.1%	7,770
richest	22.0%	6,245	moderate	51.1%	15,236
**Employment status**			high	17.8%	5,321
employed	16.8%	5,010	**Smoking status**		
retired	74.0%	22,081	never smoked	36.6%	10,931
other	9.2%	2,748	ex-smoker	52.1%	15,551
**Social class**			current smoker	11.2%	3,351
managerial	32.2%	9,458	**Date/Day orientation**		
intermediate	14.0%	4,093	All dates/day incorrect	0.7%	198
self-employed	11.7%	3,434	3 incorrect	0.6%	192
lower supervisory	10.5%	3,083	2 incorrect	1.9%	579
semi-routine	31.6%	9,270	1 incorrect	17.9%	5357
**Cohabiting status**			All dates/day correct	78.8%	23548
living alone	33.2%	9,909	**Access to car**		
living with partner	66.9%	19,985	yes access to car	83.4%	24,944
**Memory test (n of words)**	9.9 (3.6)	29,833	no access to car	16.6%	4,950

For peer review only

Table 2 reports the mean walking speed by frequency of public transport use at waves 2-6. Those who did not use public transport due to health problems had the slowest walking speed, while those who did not use public transport because of structural reasons had the fastest walking speed. There was no pattern of slower walking speed in later waves due to refreshment samples which resulted in the mean age at wave 6 being younger than at wave 2.

Table 2 Weighted mean (95% CI) of walking speed (m/s) by frequency of public transport use at waves 2-6 of ELSA

	every day or nearly every day	two or three times a week	once a week	no use: no need	no use: health problems	no use: structural reasons
wave 2	0.79 (0.77, 0.81)	0.79 (0.77, 0.81)	0.85 (0.84, 0.87)	0.84 (0.83, 0.86)	0.45 (0.42, 0.47)	0.90 (0.89, 0.92)
n	652	695	1,071	1,475	333	1,252
wave 3	0.77 (0.75, 0.80)	0.79 (0.77, 0.80)	0.79 (0.76, 0.81)	0.84 (0.82, 0.85)	0.44 (0.42, 0.47)	0.90 (0.88, 0.91)
n	389	694	462	1,431	289	1,863
wave 4	0.78 (0.76, 0.81)	0.80 (0.78, 0.82)	0.85 (0.83, 0.88)	0.84 (0.83, 0.85)	0.47 (0.44, 0.5)	0.89 (0.88, 0.91)
n	499	956	556	1,584	295	2,157
wave 5	0.81 (0.78, 0.83)	0.84 (0.83, 0.86)	0.86 (0.84, 0.88)	0.88 (0.87, 0.89)	0.47 (0.44, 0.49)	0.91 (0.90, 0.93)
n	472	935	624	1,610	315	2,148
wave 6	0.81 (0.79, 0.84)	0.84 (0.83, 0.86)	0.84 (0.82, 0.87)	0.90 (0.88, 0.91)	0.49 (0.46, 0.51)	0.93 (0.91, 0.94)
n	450	906	547	1,884	346	1,881

Table 3 reports the results of the fixed effects and multilevel growth curve (random effects) models of walking speed- the full models are shown in online Supplementary Tables S2 and S3. In the fixed effects model, the linear and quadratic terms of age were negative, suggesting that as age increased, walking speed declined faster. Compared to the reference group who used public transport nearly every day, the coefficients for all the other frequency groups were negative, suggesting that using public transport nearly every day had a protective effect on walking speed. Those who did not use public transport because of health problems had the biggest decline in walking speed (-0.06 m/s), but those who did not use public transport because they did not need to, or because of structural reasons were also more likely to have a decline in walking speed (-0.02 m/s). A difference of 0.02 m/s is an extra 0.07 seconds taken to do the walking speed test. The interaction between age and frequency of use of public transport was not significant in the fixed effects model.

For peer review only

Table 3 Selected coefficients (95% CI) from the Fixed Effects and Growth Curve Models of walking speed (m/s), ELSA waves 2-6

	Fixed Effects Model	Growth Curve Model 1	Growth Curve Model 2
**Fixed Part**			
Intercept	**0.820 (0.756,0.884)**	**0.823 (0.779,0.866)**	**0.830 (0.791,0.87)**
Age centered (linear term)	**-0.008 (-0.01,-0.007)**	**-0.007 (-0.008,-0.006)**	**-0.006 (-0.007,-0.004)**
Age (quadratic term)	**-0.0003 (-0.0004,-0.0003)**	**-0.0002 (-0.0002,-0.0001)**	**-0.0002 (-0.0002,-0.0002)**
p, 2df	*<0.001*	*<0.001*	*<0.001*
Frequency of public transport use(ref: every day or nearly every day)			
two or three times a week	-0.012 (-0.024,0.0001)	-0.006 (-0.016,0.003)	-0.005 (-0.018,0.008)
once a week	**-0.020 (-0.034,-0.005)**	**-0.011 (-0.022,-0.0002)**	-0.006 (-0.019,0.008)
no use: no need	**-0.018 (-0.032,-0.003)**	**-0.011 (-0.022,-0.001)**	-0.005 (-0.018,0.008)
no use: health problems	**-0.058 (-0.075,-0.040)**	**-0.090 (-0.104,-0.077)**	**-0.115 (-0.135,-0.095)**
no use: structural reasons	**-0.020 (-0.035,-0.006)**	**-0.009 (-0.019,0.002)**	-0.002 (-0.014,0.011)
p, 5df		*<0.001*	*<0.001*
Interaction between Age (linear term) and Frequency of public transport use			
Age*two or three times a week			-0.0003 (-0.002,0.001)
Age*once a week			-0.001 (-0.002,0.0004)
Age*no use: no need			**-0.001 (-0.002,-0.00002)**
Age*no use: health problems			**0.002 (0.0001,0.003)**
Age*no use: structural reasons			**-0.001 (-0.003,-0.0002)**
p, 5df			*<0.001*
**Random Part**			
Level 2 (Individual)			
Intercept variance		0.0238	0.0238
Age centered (linear term) variance		0.00002	0.00002
Covariance of intercept and age centered		-0.0004	-0.0004
Level 1 (wave)			
Intercept		0.020	0.020
N observations (level 1)	27509	27509	27509
N clusters (level 2)	9656	9656	9656
Goodness of fit	Adj R-sq: 0.7273	Deviance: -13719.59	Deviance: -13746.54

Boldface indicates statistical significance (p<0.05)

We additionally examined whether the association between public transport use and walking speed decreased when taking into account lower limb muscle strength (chair stands) and the interaction between physical activity and age (Supplementary Table S4). Similar to the coefficients reported in Table 3, the coefficients in the model without controlling for these potential mechanisms for those who did not use public transport because they did not need to or because of structural reasons was -0.02 m/s; although due to the smaller sample size (chair stands were only collected at waves 2,4 and 6), the 95% confidence intervals overlapped 0. Once chair stands and the interaction between physical activity and age were controlled for, these coefficients reduced by about half to -

For peer review only - http://bmjopen.bmj.com/site/about/guidelines.xhtml

0.01, suggesting that some of the association between public transport use and walking speed among older adults is statistically explained by lower limb muscle strength and physical activity.

We also used fixed effects models to examine whether changes in the use/non-use of public transport (for different reasons) were predicted by walking speed and the other covariates (Supplementary Table S5). For this analysis, we grouped the frequency variable (the dependent variable) into fewer groups: (1) used public transport, the reference category (2) did not use because of no need (3) did not use because of health problems and (4) did not use because of structural reasons. Slower walking speed and poorer health did not predict changes in the use/non-use of public transport because of the lack of need or structural reasons, but slower respondents were more likely not to use public transport for health reasons. Respondents with access to a car/van were less likely to use public transport. Respondents with mobility difficulties, low physical activity levels and higher levels of depressive symptoms were more likely to not use public transport for health reasons. Living in an isolated area increased the likelihood of a person reporting not using public transport because of structural reasons.

The results of the fixed effects model predicting grip strength are shown in online Supplementary Table S6. Unsurprisingly, older adults who did not use public transport due to health problems had weaker grip strength than those who used public transport nearly/every day. However, there were no differences in grip strength between the latter group and those who did not use public transport due to structural reasons or because they did not need to. In contrast, not using public transport because of structural barriers was associated with decrements in lower limb muscle strength. Such ELSA participants were more likely to become unable to complete the chair stand test, relative to those who completed the test quicker (Supplementary Table S7). Furthermore, older ELSA respondents who did not use public transport because they did not need to or because of structural barriers were also more likely to become sedentary relative to those engaging in high physical activity (Supplementary Table S8).

Turning to RQ3, in the 1st growth curve model (Table 3, column 3), we see similar estimates for the intercept and age coefficients compared to the fixed effects model. All the coefficients for the frequency variable were also negative, although the negative effect of not using public transport due to structural reasons on walking speed was small and not different from those who used public transport nearly every day (the reference group).

For peer review only - http://bmjopen.bmj.com/site/about/guidelines.xhtml

The second growth curve model added in the interaction between the frequency and age. With increasing age, the effect on walking speed of not using public transport for structural reasons or because the respondent did not feel the need to, became increasingly more negative. The trajectories of these three groups (those who used public transport nearly every day, those who did not use because of structural reasons, and those who did not use because they did not need to) are shown in Figures 1 and 2. In both figures, the decline in walking speed with age started to diverge around age 75, when there was a slower decline in walking speed for those who used public transport nearly every day, and a much steeper decline for those who did not use public transport because of structural reasons, or a lack of need. The upper 95% confidence intervals of the latter two groups clearly did not overlap with the estimated trajectories of the frequent public transport users after about the age of 75. We also estimated the growth curve model for those participants with at least three waves of walking speed measurements (Supplementary file Table S9) and found very similar estimates to the sample including all ELSA participants with at least one walking speed measurement (Table S3).

[Fig.1 and Fig.2 here]

Discussion

This study found evidence that older adults living in England who frequently used public transport had faster walking speeds than their peers who did not use public transport. Results from fixed effects and multilevel growth curve models showed similar patterns. In fixed effects models, frequent public transport use among older adults had a protective effect on walking speed. Unsurprisingly, not using public transport due to health reasons had the largest negative effect on walking speed. However, not using public transport due to other reasons also had a negative effect on walking speed. While the effect size of 0.02 m/s associated with not using public transport due to structural reasons may appear small, the predicted levels of walking speed in this cohort of older adults were well below the recommended 1.2 m/s walking speed needed for standard pedestrian crossings. [31] Any increase in the walking speed of older adults through factors such as physical activity and increased public transport use may help them cross the road safely.

The results of the fixed effects models were corroborated by the trajectories of walking speed decline shown in the growth curve models. In the growth curve models, older adults who did not use public transport due to structural reasons or because of a lack of need (“through choice”) had a faster decline in walking speed after the age of 75 than those who used public transport nearly every day.

For peer review only - http://bmjopen.bmj.com/site/about/guidelines.xhtml

The association between public transport use and muscle function deficits was specific to lower limb muscle strength, and did not extend to another aging related upper body muscle function deficit, grip strength. Frequent use of public transport appears to delay declines in muscles involved in walking, which in turn impacts on walking speed and related physical activity, not other aging related muscle function declines. The specificity of the association also suggests that potential confounders related to strength, fitness, and health were unlikely to cause the public transport use-walking speed association.

Existing studies have found that use of public transport contributes to better health by increasing physical activity [11 12 18] and reducing obesity. [18 19 23] However these studies have not examined the reasons why people do not use public transport. Limiting health is potentially a key factor that could confound any association between public transport use and subsequent health. The use of repeated measurements of public transport use (and the underlying reasons for non-use) and walking speed from a large, representative sample of older adults has been useful in taking account of this key confounding factor. Respondents who "use public transport nearly every day" may be positively health selected. In the analyses, we take account of changes in health conditions in a number of ways. First we control for different health conditions (depression, mobility problems, ADL) that vary across waves. Secondly, respondents could select limiting health as the main reason why they could not use public transport- this is the main negative health selection group. Moreover, we found little evidence that respondents with poorer health and slower walking speeds were more likely to report not using public transport because they did not need to or because of structural barriers.

Limitations

Longitudinal attrition across waves and other missing data may have resulted in a biased sample. The longitudinal sample tended to be older, healthier and more socio-economically advantaged. We used the wave specific cross sectional survey weights, which takes account of such predictors of non-response, in order to make the analyses representative of the older population but this may not adequately deal with attrition biases. Furthermore, there may be unobserved factors that cause the association between public transport use and walking speed.

Conclusion

For peer review only - http://bmjopen.bmj.com/site/about/guidelines.xhtml

It has become increasingly important for research to show a positive health impact from public transport use, especially among older adults, given cuts to public transport availability in England. [16] Savings to local government from cutting public transport may result in future increased expenditure on aging related conditions. Older adults who do not use public transport frequently are at risk of faster declines in their physical activity, lower limb muscle strength and walking speed compared to those who use public transport every day. This risk was evident not just among older adults who did not use public transport because of health problems, but also among those who did not use public transport because of structural barriers.

For peer review only

Acknowledgements

The data were made available through the UK Data Archive. ELSA was developed by a team of researchers based at the NatCen Social Research, University College London and the Institute for Fiscal Studies. The data were collected by NatCen Social Research. The funding is provided by the National Institute of Aging in the United States, and a consortium of UK government departments co-ordinated by the Office for National Statistics. The developers and funders of ELSA and the Archive do not bear any responsibility for the analyses or interpretations presented here.

PR participated in a Newton Fund/British Council/CONFAP Researcher Links Workshop, "Indicators for a Healthy City", in Belo Horizonte, Brazil, May 2016, which was coordinated by Professor Jennifer Mindell (UCL) and Professor Waleska Caiaffa (Federal University of Minas Gerais, UFMG).

Contributors: PR is responsible for study conception, study design, data analysis, and manuscript preparation and revision; had access to all of the data in the study; and takes responsibility for the integrity of the data and the accuracy of the data analysis. TC contributed to conception and design of the study, data analysis, data interpretation, manuscript preparation and revision. EW contributed to the design of the study, manuscript revision and provided input on the analysis of the study. All authors read and approved the final manuscript.

Funding: PR was supported by a grant from the Economic and Social Research Council [ES/P010075/1]. EW and TC were supported by a grant from the Economic and Social Research Council [ES/J019119/1]. TC was supported by a grant from the Medical Research Council [G1001375].

Disclaimer: The sponsors had no role in the study design; collection, analysis, and interpretation of the data; writing the report; or the decision to submit the report for publication.

Ethics approval: Ethical approval for all the ELSA waves was granted from NHS Research Ethics Committees under the National Research and Ethics Service (NRES). For further information see: http://www.nres.nhs.uk/.

Competing interest: None declared.

Data sharing statement: No additional data are available.

For peer review only

References

1. Cooper R, Kuh D, Hardy R. Objectively measured physical capability levels and mortality: systematic review and meta-analysis. *BMJ* 2010;341:c4467. doi: 10.1136/bmj.c4467 [published Online First: 2010/09/11]

2. Cooper R, Kuh D, Cooper C, et al. Objective measures of physical capability and subsequent health: a systematic review. *Age Ageing* 2011;40(1):14-23. doi: 10.1093/ageing/afq117 [published Online First: 2010/09/17]

3. Risser R, Lexell EM, Bell D, et al. Use of local public transport among people with cognitive impairments – A literature review. *Transportation Research Part F: Traffic Psychology and Behaviour* 2015;29:83-97. doi: 10.1016/j.trf.2015.01.002

4. Schwanen T, Lucas K, Akyelken N, et al. Rethinking the links between social exclusion and transport disadvantage through the lens of social capital. *Transportation Research Part A: Policy and Practice* 2015;74:123-35. doi: 10.1016/j.tra.2015.02.012

5. Green J, Jones A, Roberts H. More than A to B: the role of free bus travel for the mobility and wellbeing of older citizens in London. *Ageing and Society* 2012;34(3):472-94. doi: 10.1017/S0144686X12001110

6. Correa-de-Araujo R, Hadley E. Skeletal muscle function deficit: a new terminology to embrace the evolving concepts of sarcopenia and age-related muscle dysfunction. *J Gerontol A Biol Sci Med Sci* 2014;69(5):591-4. doi: 10.1093/gerona/glt208 [published Online First: 2014/04/17]

7. Montero-Fernandez N, Serra-Rexach JA. Role of exercise on sarcopenia in the elderly. *Eur J Phys Rehabil Med* 2013;49(1):131-43. [published Online First: 2013/04/12]

8. Freiberger E, Sieber C, Pfeifer K. Physical activity, exercise, and sarcopenia - future challenges. *Wien Med Wochenschr* 2011;161(17-18):416-25. doi: 10.1007/s10354-011-0001-z [published Online First: 2011/07/28]

9. Samitz G, Egger M, Zwahlen M. Domains of physical activity and all-cause mortality: systematic review and dose-response meta-analysis of cohort studies. *Int J Epidemiol* 2011;40(5):1382-400. doi: 10.1093/ije/dyr112

10. Barr A, Bentley R, Simpson JA, et al. Associations of public transport accessibility with walking, obesity, metabolic syndrome and diabetes. *Journal of Transport & Health* 2016;3(2):141-53. doi: 10.1016/j.jth.2016.01.006

11. Rissel C, Curac N, Greenaway M, et al. Physical Activity Associated with Public Transport Use—A Review and Modelling of Potential Benefits. *Int J Environ Res Public Health* 2012;9(7):2454-78. doi: 10.3390/ijerph9072454

12. Saelens BE, Vernez Moudon A, Kang B, et al. Relation Between Higher Physical Activity and Public Transit Use. *Am J Public Health* 2014;104(5):854-59. doi: 10.2105/AJPH.2013.301696

13. Mackett R. Improving accessibility for older people – Investing in a valuable asset. *Journal of Transport & Health* 2015;2(1):5-13. doi: 10.1016/j.jth.2014.10.004

14. Mackett R. Overcoming the Barriers to Access for Older People. Report Produced for the Age Action Alliance 2014. http://ageactionalliance.org/wordpress/wp-content/uploads/2014/11/Overcoming-the-barriers-to-access-Nov-14.pdf (accessed 10 Nov 2016).

15. Syed ST, Gerber BS, Sharp LK. Traveling towards disease: transportation barriers to health care access. *J Community Health* 2013;38(5):976-93. doi: 10.1007/s10900-013-9681-1

For peer review only

16. Department for Transport. Annual bus statistics: England 2015/16 2016. https://www.gov.uk/government/uploads/system/uploads/attachment_data/file/561060/annual-bus-statistics-year-ending-march-2016.pdf (accessed 10 Nov 2016).

17. Holley-Moore G, Creighton H. The Future of Transport in an Ageing Society London: ILC-UK; 2015. http://www.ilcuk.org.uk/index.php/publications/publication_details/the_future_of_transport_in_an_ageing_society (accessed 10 Nov 2016).

18. Webb E, Laverty A, Mindell J, et al. Free Bus Travel and Physical Activity, Gait Speed, and Adiposity in the English Longitudinal Study of Ageing. *Am J Public Health* 2016;106(1):136-42. doi: 10.2105/AJPH.2015.302907

19. Webb E, Netuveli G, Millett C. Free bus passes, use of public transport and obesity among older people in England. *J Epidemiol Community Health* 2012;66(2):176-80. doi: 10.1136/jech.2011.133165

20. Coronini-Cronberg S, Millett C, Laverty AA, et al. The impact of a free older persons' bus pass on active travel and regular walking in England. *Am J Public Health* 2012;102(11):2141-8. doi: 10.2105/ajph.2012.300946 [published Online First: 2012/09/22]

21. Weber D. Differences in physical aging measured by walking speed: evidence from the English Longitudinal Study of Ageing. *BMC Geriatr* 2016;16:31. doi: 10.1186/s12877-016-0201-x [published Online First: 2016/01/30]

22. Zaninotto P, Sacker A, Head J. Relationship between wealth and age trajectories of walking speed among older adults: evidence from the English Longitudinal Study of Ageing. *J Gerontol A Biol Sci Med Sci* 2013;68(12):1525-31. doi: 10.1093/gerona/glt058 [published Online First: 2013/05/18]

23. Flint E, Webb E, Cummins S. Change in commute mode and body-mass index: prospective, longitudinal evidence from UK Biobank. *The Lancet Public Health* 2016 doi: 10.1016/S2468-2667(16)30006-8

24. Broome K, McKenna K, Fleming J, et al. Bus use and older people: A literature review applying the Person–Environment–Occupation model in macro practice. *Scand J Occup Ther* 2009;16(1):3-12. doi: 10.1080/11038120802326222

25. Broome K, Nalder E, Worrall L, et al. Age-friendly buses? A comparison of reported barriers and facilitators to bus use for younger and older adults. *Australasian journal on ageing* 2010;29(1):33-8. doi: 10.1111/j.1741-6612.2009.00382.x [published Online First: 2010/04/20]

26. Steptoe A, Breeze E, Banks J, et al. Cohort Profile: The English Longitudinal Study of Ageing. *Int J Epidemiol* 2013,6(42):1640-09. doi:10.1093/ije/dys168.

27. Katz S, Ford AB, Moskowitz RW, et al. Studies of illness in the aged. The index of ADL: A standardized measure of biological and psychological function. *JAMA. 1963;185(12):914-919. doi:10.1001/jama.1963.03060120024016.*

28. Radloff LS. The CES-D Scale: A Self-Report Depression Scale for Research in the General Population. *Appl Psychol Meas* 1977;1(3):385-401. doi: 10.1177/014662167700100306

29. Demakakos P, Hamer M, Stamatakis E, et al. Low-intensity physical activity is associated with reduced risk of incident type 2 diabetes in older adults: evidence from the English Longitudinal Study of Ageing. *Diabetologia* 2010;53(9):1877-85. doi: 10.1007/s00125-010-1785-x [published Online First: 2010/05/25]

30. Bridges S, Hussey D, Blake M. The dynamics of ageing: The 2012 English Longitudinal Study of Ageing (Wave 6) Technical Report. 2015. http://doc.ukdataservice.ac.uk/doc/5050/mrdoc/pdf/5050_elsa_w6_technical_report_v1.pdf (accessed 15 June 2017).

For peer review only

31. Bohannon RW, Williams Andrews A. Normal walking speed: a descriptive meta-analysis. *Physiotherapy* 2011;97(3):182-9. doi: 10.1016/j.physio.2010.12.004 [published Online First: 2011/08/09]

For peer review only

Figure Captions

Figure 1

Predicted decline in walking speed with age by public transport use, comparing ELSA respondents who use public transport every day and those who do not use public transport because of structural reasons

Figure 2

Predicted decline in walking speed with age by public transport use, comparing ELSA respondents who use public transport every day and those who do not use public transport because they do not need to

For peer review only

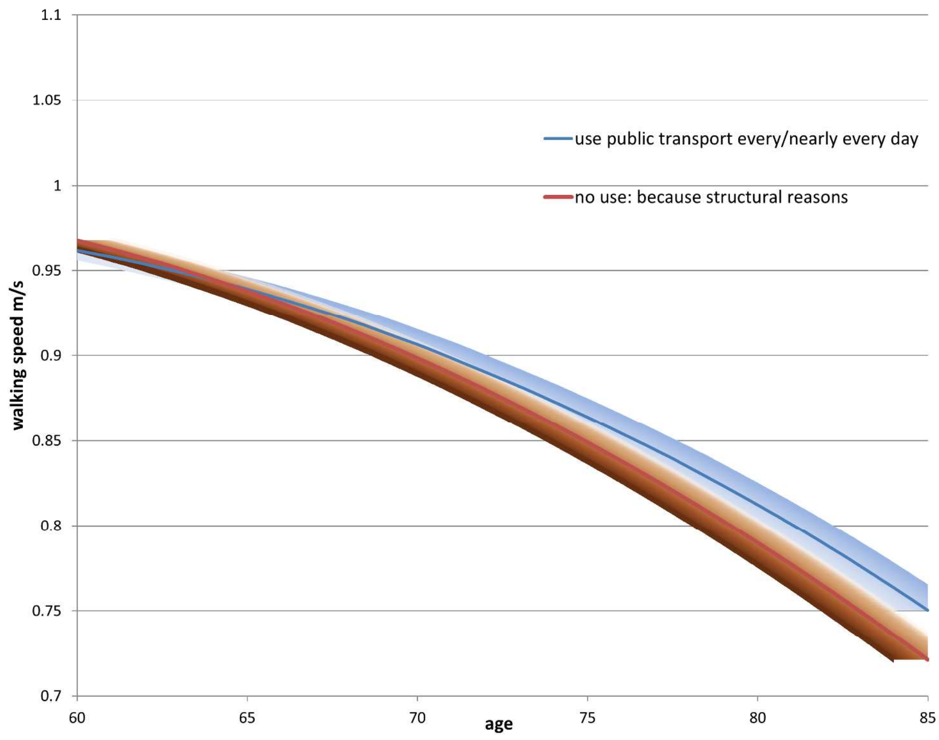

Predicted decline in walking speed with age by public transport use, comparing ELSA respondents who use public transport every day and those who do not use public transport because of structural reasons

254x190mm (300 x 300 DPI)

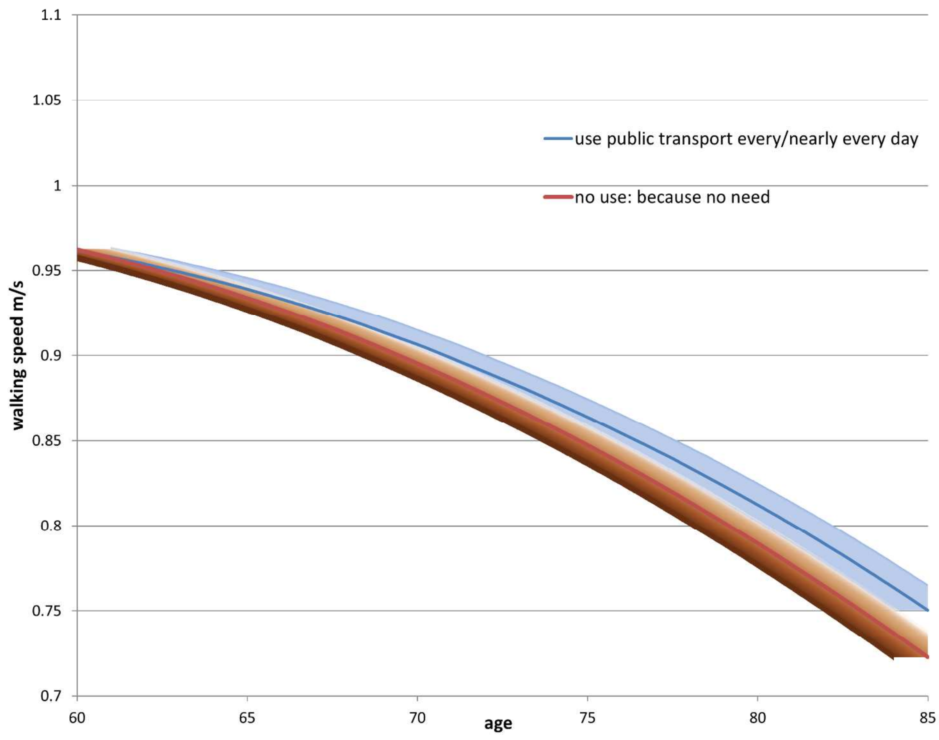

Predicted decline in walking speed with age by public transport use, comparing ELSA respondents who use public transport every day and those who do not use public transport because they do not need to

254x190mm (300 x 300 DPI)

Does public transport use prevent declines in walking speed among older adults living in England? A prospective cohort study

Patrick Rouxel, PhD[1], Elizabeth Webb, PhD [2] and Tarani Chandola PhD [3]

[1]CLOSER, Department of Social Science, University College London Institute of Education, London, UK, ORCID: 0000-0003-0330-554X, [2] International Centre for Lifecourse Studies, Department of Epidemiology and Public Health, University College London, London, UK, ORCID: 0000-0001-6106-5367, and [3]CMIST and Social Statistics, University of Manchester, UK, ORCID: 0000-0002-1864-3413

To BMJ Open

Corresponding author

Patrick Rouxel, PhD, CLOSER, Department of Social Science, University College London Institute of Education, 55-59 Gordon Square, WC1H 0NU, London, UK. E-mail: patrick.rouxel@ucl.ac.uk

For peer review only

Supplementary Table S1 Log odds (95% CI) from logit model of any missing data in the walking speed analyses, conditional on having a baseline walking speed measurement: ELSA waves 2-6

	Log odds	Lower 95% CI	Upper 95% CI
Intercept	**0.54**	-0.17	1.26
Gender (ref: Male)	**-0.22**	-0.31	-0.13
Age centered (linear term)	**-0.02**	-0.03	-0.01
*Gender*Age (ref: Male)*	**-0.01**	-0.02	-0.004
Age (quadratic term)	**0.01**	0.01	0.01
Frequency of public transport use(ref: every day or nearly every day)			
two or three times a week	-0.07	-0.24	0.09
once a week	**-0.32**	-0.48	-0.16
did not use because no need	0.02	-0.12	0.17
did not use because health problems	**0.69**	0.43	0.95
did not use because structural reasons	0.04	-0.11	0.19
Access to car/van (ref: yes access)			
No access to car/van	**0.25**	0.11	0.38
Employment status (ref: employed)			
Retired	**0.14**	0.02	0.25
Other	0.03	-0.10	0.15
Social class (ref: Managerial & Prof)			
Intermediate occupations	0.03	-0.09	0.16
Self-employed	**0.14**	0.01	0.27
Lower supervisory & technical	0.05	-0.09	0.18
Semi-routine & routine	**0.21**	0.11	0.31
Urban/Rural (ref: Urban)			
Town & Fringe	0.02	-0.10	0.13
Village	-0.13	-0.25	0.002
Hamlet/Isolated	0.08	-0.12	0.27
Mobility difficulties (ref: none)			
1-3 difficulties	-0.05	-0.14	0.04
4+ difficulties	**0.15**	0.01	0.29
Activities of Daily living impairments (ref: none)			
At least one ADL impairment	**0.16**	0.03	0.28
Physical Activity levels (ref: sedentary)			
Low	**-0.80**	-1.04	-0.55
Moderate	**-0.98**	-1.22	-0.74
High	**-1.00**	-1.25	-0.74
Marital status (ref: married)			
Separated/Divorced	**0.43**	0.25	0.61
Widowed	**0.45**	0.24	0.65
Never married	**0.66**	0.43	0.89
Cohabitation (ref: not living with partner)			
Living with partner	**0.69**	0.51	0.88
CESD- Depression score	**0.04**	0.01	0.06

For peer review only

Supplementary Table S1 continued Log odds (95% CI) from logit model of any missing data in the walking speed analyses, conditional on having a baseline walking speed measurement: ELSA waves 2-6

	**Log odds**	**Lower 95% CI**	**Upper 95% CI**
Smoking status (ref: never smoker)			
Ex-Smoker	-0.02	-0.10	0.06
Current smoker	**0.25**	0.13	0.36
Date/Day orientation (ref: no correct answer)			
1 correct answer	0.005	-0.93	0.94
2 correct answers	0.04	-0.67	0.75
3 correct answers	-0.47	-1.10	0.16
All correct answers	-0.52	-1.14	0.11
Memory test	**-0.06**	-0.07	-0.04

Boldface indicates statistical significance ($p<0.05$)

For peer review only

Supplementary Table S2 Coefficients (95% CI) from the Fixed Effects Model of walking speed (m/s), ELSA waves 2-6

	Coefficients	Lower 95% CI	Upper 95% CI
Intercept	**0.82**	0.76	0.88
Age centered (linear term)	**-0.01**	-0.01	-0.01
Age (quadratic term)	**-0.0003**	-0.0004	-0.0003
Frequency of public transport use(ref: every day or nearly every day)			
two or three times a week	-0.01	-0.02	0.0001
once a week	**-0.02**	-0.03	-0.01
did not use because no need	**-0.02**	-0.03	-0.003
did not use because health problems	**-0.06**	-0.08	-0.04
did not use because structural reasons	**-0.02**	-0.03	-0.01
Wealth quintiles (ref: poorest quintile)			
Quintile 2	0.01	-0.01	0.02
Quintile 3	0.01	-0.01	0.03
Quintile 4	0.02	-0.01	0.04
Richest quintile	**0.03**	0.003	0.05
Access to car/van (ref: yes access)			
No access to car/van	-0.01	-0.02	0.0005
Employment status (ref: employed)			
Retired	0.00	-0.01	0.01
Other	0.00	-0.01	0.02
Social class (ref: Managerial & Prof)			
Intermediate occupations	-0.02	-0.06	0.02
Self-employed	0.01	-0.03	0.05
Lower supervisory & technical	-0.02	-0.06	0.03
Semi-routine & routine	0.02	-0.02	0.06
Urban/Rural (ref: Urban)			
Town & Fringe	0.02	-0.03	0.06
Village	0.01	-0.03	0.05
Hamlet/Isolated	0.03	-0.03	0.08
Mobility difficulties (ref: none)			
1-3 difficulties	**-0.01**	-0.02	-0.01
4+ difficulties	**-0.05**	-0.06	-0.04
Activities of Daily living impairments (ref: none)			
At least one ADL impairment	**-0.03**	-0.04	-0.02
Physical Activity levels (ref: sedentary)			
Low	**0.03**	0.02	0.04
Moderate	**0.05**	0.04	0.07
High	**0.06**	0.05	0.08
Marital status (ref: married)			
Separated/Divorced	**0.04**	0.01	0.07
Widowed	0.01	-0.02	0.04
Never married	0.02	-0.04	0.07
Cohabitation (ref: not living with partner)			
Living with partner	0.01	-0.02	0.05
CESD- Depression score	-0.01	-0.01	-0.01

For peer review only

Supplementary Table S2 continued Coefficients (95% CI) from the Fixed Effects Model of walking speed (m/s), ELSA waves 2-6

Smoking status (ref: never smoker)	**Coefficients**	**Lower 95% CI**	**Upper 95% CI**
Ex-Smoker	0.00	-0.02	0.03
Current smoker	0.01	-0.02	0.04
Date/Day orientation (ref: no correct answer)			
1 correct answer	0.02	-0.03	0.07
2 correct answers	0.04	-0.01	0.08
3 correct answers	**0.05**	0.01	0.09
All correct answers	**0.05**	0.01	0.09
Memory test	**0.001**	0.00002	0.002
Number of observations	27509		
Adjusted R-squared	0.73		

Boldface indicates statistical significance (p<0.05)

For peer review only

Supplementary Table S3 Coefficients (95% CI) from the Multilevel Growth Curve Model of walking speed (m/s), ELSA waves 2-6

**Fixed Part**	**Coefficients**	**Lower 95% CI**	**Upper 95% CI**
Intercept	**0.83**	0.79	0.87
Age centered (linear term)	**-0.01**	-0.01	-0.004
Age (quadratic term)	**-0.0002**	-0.0002	-0.0002
Frequency of public transport use(ref: every day or nearly every day)			
two or three times a week	-0.01	-0.02	0.01
once a week	-0.01	-0.02	0.01
did not use because no need	-0.005	-0.02	0.01
did not use because health problems	**-0.12**	-0.14	-0.09
did not use because structural reasons	-0.002	-0.014	0.011
Interaction between Age (linear term) and Frequency of public transport use (ref: nearly/every day use)			
Age*two or three times a week	-0.0003	-0.0017	0.0011
Age*once a week	-0.0010	-0.0024	0.0004
Age*did not use because no need	**-0.0012**	-0.0024	-0.00002
Age*did not use because health problems	**0.0017**	0.0001	0.0033
Age*did not use because structural reasons	**-0.0014**	-0.0026	-0.0002
Sex (ref: men)			
Women	**-0.02**	-0.03	-0.02
Wealth quintiles (ref: poorest quintile)			
Quintile 2	**0.02**	0.01	0.03
Quintile 3	**0.04**	0.03	0.05
Quintile 4	**0.06**	0.05	0.07
Richest quintile	**0.09**	0.08	0.10
Access to car/van (ref: yes access)			
No access to car/van	**-0.02**	-0.03	-0.02
Employment status (ref: employed)			
Retired	-0.01	-0.02	0.00002
Other	**-0.02**	-0.03	-0.01
Social class (ref: Managerial & Prof)			
Intermediate occupations	**-0.02**	-0.03	-0.01
Self-employed	**-0.02**	-0.03	-0.01
Lower supervisory & technical	**-0.05**	-0.06	-0.04
Semi-routine & routine	**-0.04**	-0.05	-0.03
Urban/Rural (ref: Urban)			
Town & Fringe	**0.01**	0.003	0.02
Village	**0.02**	0.01	0.03
Hamlet/Isolated	**0.02**	0.00	0.04
Mobility difficulties (ref: none)			
1-3 difficulties	**-0.04**	-0.04	-0.03
4+ difficulties	**-0.12**	-0.13	-0.11
Activities of Daily living impairments (ref: none)			
At least one ADL impairment	**-0.06**	-0.06	-0.05

For peer review only

Supplementary Table S3 continued Coefficients (95% CI) from the Multilevel Growth Curve Model of walking speed (m/s), ELSA waves 2-6

Physical Activity levels (ref: sedentary)	**Coefficients**	**Lower 95% CI**	**Upper 95% CI**
Low	**0.05**	0.04	0.06
Moderate	**0.10**	0.08	0.11
High	**0.12**	0.11	0.13
Marital status (ref: married)			
Separated/Divorced	0.001	-0.01	0.01
Widowed	-0.003	-0.01	0.01
Never married	**-0.03**	-0.05	-0.01
Cohabitation (ref: not living with partner)			
Living with partner	0.01	-0.01	0.03
CESD- Depression score	**-0.01**	-0.01	-0.01
Smoking status (ref: never smoker)			
Ex-Smoker	-0.01	-0.01	0.001
Current smoker	**-0.03**	-0.04	-0.02
Date/Day orientation (ref: no correct answer)			
1 correct answer	0.01	-0.03	0.05
2 correct answers	**0.03**	-0.01	0.07
3 correct answers	**0.05**	0.02	0.08
All correct answers	**0.05**	0.02	0.09
Memory test	**0.01**	0.004	0.01
**Random Part**			
Level 2 (Individual)			
Intercept variance	0.02		
Age centered (linear term) variance	0.00002		
Covariance of intercept and age centered	-0.0004		
Level 1 (wave)			
Intercept	0.02		
Number of observations (level 1)	9656		
Number of clusters (level 2)	27509		
-2*Log Likelihood	-13746.54		

Boldface indicates statistical significance (p<0.05)

For peer review only

Supplementary Table S4 Selected coefficients (95% CI) from two Fixed Effects Models of walking speed (m/s), ELSA waves 2-6

**Model 1: excluding chair stand and physical activity**	**Coefficients**	**Lower 95% CI**	**Upper 95% CI**
Frequency of public transport use(ref: every day or nearly every day)			
two or three times a week	-0.02	-0.04	0.01
once a week	**-0.03**	-0.05	-0.001
did not use because no need	-0.02	-0.04	0.01
did not use because health problems	**-0.08**	-0.11	-0.05
did not use because structural reasons	-0.02	-0.04	0.01

Model 1 includes age, age squared, frequency of public transport use, gender, wealth, car/van access, employment status, social class, urban/rural, mobility difficulties, disability, marital status, cohabitation, CES-D depression score, smoking status memory test, time/date orientation

**Model 2: including chair stand and physical activity**	**Coefficients**	**Lower 95% CI**	**Upper 95% CI**
Frequency of public transport use(ref: every day or nearly every day)			
two or three times a week	-0.01	-0.03	0.01
once a week	-0.02	-0.05	0.003
did not use because no need	-0.01	-0.04	0.01
did not use because health problems	**-0.06**	-0.09	-0.03
did not use because structural reasons	-0.01	-0.04	0.01

Model 2 includes all the variables in Model 1 as well as chair stand, physical activity and the interaction between physical activity and age. Model 1 is nested within Model 2.

Boldface indicates statistical significance (p<0.05)

For peer review only

Supplementary Table S5 Coefficients (95% CI) from the Fixed Effects multinomial model of public transport use, ELSA waves 2-6. Coefficients are log odds of not using public transport for different reasons relative to using public transport at least once a week

	**no use: no need**			**no use: health problems**			**no use: structural reasons**		
	Log odds	Lower 95% CI	Upper 95% CI	Log odds	Lower 95% CI	Upper 95% CI	Log odds	Lower 95% CI	Upper 95% CI
Walking speed	-0.16	-0.55	0.22	**-2.39**	-3.29	-1.48	-0.26	-0.64	0.12
Age centered (linear term)	**0.05**	0.02	0.08	**0.12**	0.04	0.19	-0.003	-0.03	0.03
Age (quadratic term)	**0.004**	0.002	0.01	**0.01**	0.01	0.01	**0.005**	0.003	0.01
Wealth quintiles (ref: poorest quintile)									
Quintile 2	-0.33	-0.76	0.10	**-0.96**	-1.80	-0.11	-0.20	-0.69	0.29
Quintile 3	-0.24	-0.70	0.22	-0.73	-1.64	0.17	-0.18	-0.69	0.34
Quintile 4	-0.15	-0.64	0.34	-0.82	-1.81	0.16	-0.02	-0.56	0.52
Richest quintile	-0.38	-0.91	0.14	-1.12	-2.28	0.04	-0.30	-0.87	0.28
Access to car/van (ref: yes access)									
No access to car/van	**-1.20**	-1.49	-0.90	**-0.57**	-0.97	-0.16	**-1.02**	-1.35	-0.68
Employment status (ref: employed)									
Retired	-0.18	-0.44	0.09	0.42	-0.61	1.45	**-0.36**	-0.62	-0.10
Other	-0.16	-0.49	0.17	0.51	-0.57	1.59	**-0.45**	-0.78	-0.12
Social class (ref: Managerial & Prof)									
Intermediate occupations	-0.11	-0.97	0.74	-14.63	-1940.9	1911.7	-0.23	-1.05	0.58
Self-employed	-0.35	-1.19	0.48	-2.87	-6.39	0.65	**-0.89**	-1.72	-0.06
Lower supervisory & technical	0.89	-0.16	1.94	-0.50	-5.30	4.29	0.51	-0.49	1.50
Semi-routine & routine	-0.36	-1.15	0.44	-2.35	-4.81	0.11	-0.72	-1.47	0.04
Urban/Rural (ref: Urban)									
Town & Fringe	**0.75**	0.01	1.49	-1.20	-2.78	0.38	0.60	-0.06	1.25
Village	0.38	-0.62	1.37	1.23	-0.39	2.84	0.59	-0.24	1.43
Hamlet/Isolated	0.29	-1.19	1.77	-0.90	-4.16	2.35	**2.01**	0.66	3.37

For peer review only - http://bmjopen.bmj.com/site/about/guidelines.xhtml

Supplementary Table S5 continued Coefficients (95% CI) from the Fixed Effects multinomial model of public transport use, ELSA waves 2-6. Coefficients are log odds of not using public transport for different reasons relative to using public transport at least once a week

	**no use: no need**			**no use: health problems**			**no use: structural reasons**		
Mobility difficulties (ref:none)	Log odds	Lower 95% CI	Upper 95% CI	Log odds	Lower 95% CI	Upper 95% CI	Log odds	Lower 95% CI	Upper 95% CI
1-3 difficulties	-0.10	-0.26	0.06	**0.60**	0.07	1.14	0.03	-0.13	0.20
4+ difficulties	-0.06	-0.33	0.21	**1.43**	0.83	2.03	0.26	-0.01	0.54
Activities of Daily living impairments (ref: none)									
At least one ADL impairment	0.10	-0.11	0.31	0.16	-0.15	0.46	0.06	-0.15	0.27
Physical Activity levels (ref: sedentary)									
Low	0.01	-0.32	0.35	-0.26	-0.67	0.14	-0.24	-0.58	0.11
Moderate	0.09	-0.24	0.43	**-0.57**	-1.04	-0.09	-0.26	-0.62	0.09
High	0.10	-0.27	0.48	**-0.98**	-1.77	-0.19	-0.27	-0.65	0.12
Marital status (ref: married)									
Separated/Divorced	0.17	-0.56	0.90	-0.98	-2.41	0.45	0.44	-0.30	1.18
Widowed	-0.01	-0.67	0.65	-0.19	-1.39	1.01	-0.28	-0.96	0.39
Never married	0.57	-0.78	1.92	-0.68	-3.87	2.52	0.29	-1.13	1.71
Cohabitation (ref: not living with partner)									
Living with partner	0.61	-0.02	1.24	0.22	-1.00	1.44	0.34	-0.30	0.98
CESD- Depression score	-0.04	-0.08	0.01	**0.10**	0.02	0.18	0.002	-0.04	0.05
Smoking status (ref: never smoker)									
Ex-Smoker	-0.24	-0.77	0.29	**-1.18**	-2.21	-0.14	-0.27	-0.82	0.27
Current smoker	0.12	-0.55	0.79	-0.67	-1.94	0.59	-0.04	-0.73	0.65
Date/Day orientation (ref: no correct answer)									
1 correct answer	1.02	-0.23	2.27	1.06	-0.45	2.57	0.69	-0.60	1.99
2 correct answers	0.70	-0.40	1.79	0.38	-1.01	1.77	0.53	-0.61	1.67
3 correct answers	0.49	-0.53	1.52	0.52	-0.82	1.86	0.13	-0.94	1.19
All correct answers	0.56	-0.46	1.58	0.55	-0.79	1.88	0.11	-0.95	1.17
Memory test	0.00	-0.03	0.02	0.00	-0.05	0.05	-0.01	-0.04	0.01

Boldface indicates statistical significance ($p<0.05$)

For peer review only - http://bmjopen.bmj.com/site/about/guidelines.xhtml

Supplementary Table S6 Coefficients (95% CI) from the Fixed Effects Model of grip strength (kg), ELSA waves 2-6

	Coefficients	Lower 95% CI	Upper 95% CI
Intercept	**28.47**	26.21	30.72
Age centered (linear term)	**-0.44**	-0.47	-0.41
Age (quadratic term)	**-0.005**	-0.007	-0.003
Frequency of public transport use(ref: every day or nearly every day)			
two or three times a week	-0.37	-0.88	0.14
once a week	-0.12	-0.68	0.44
did not use because no need	-0.03	-0.63	0.56
did not use because health problems	**-1.35**	-2.09	-0.61
did not use because structural reasons	-0.13	-0.70	0.45
Wealth quintiles (ref: poorest quintile)			
Quintile 2	-0.42	-1.17	0.33
Quintile 3	-0.35	-1.15	0.46
Quintile 4	-0.32	-1.17	0.54
Richest quintile	-0.06	-1.00	0.88
Access to car/van (ref: yes access)			
No access to car/van	0.08	-0.40	0.56
Employment status (ref: employed)			
Retired	-0.22	-0.67	0.22
Other	-0.96	-1.59	-0.33
Social class (ref: Managerial & Prof)			
Intermediate occupations	0.28	-1.43	1.99
Self-employed	-0.39	-1.44	0.65
Lower supervisory & technical	-0.76	-1.98	0.47
Semi-routine & routine	0.22	-0.86	1.31
Urban/Rural (ref: Urban)			
Town & Fringe	0.31	-0.65	1.27
Village	0.08	-1.16	1.32
Hamlet/Isolated	0.05	-1.35	1.45
Mobility difficulties (ref: none)			
1-3 difficulties	-0.19	-0.47	0.08
4+ difficulties	-1.03	-1.50	-0.57
Activities of Daily living impairments (ref: none)			
At least one ADL impairment	**-0.69**	-1.07	-0.30
Physical Activity levels (ref: sedentary)			
Low	**0.69**	0.07	1.30
Moderate	**0.73**	0.10	1.37
High	**0.95**	0.24	1.67
Marital status (ref: married)			
Separated/Divorced	-0.81	-1.90	0.28
Widowed	-0.23	-1.32	0.87
Never married	-1.47	-3.75	0.82
Cohabitation (ref: not living with partner)			
Living with partner	-0.47	-1.56	0.63
CESD- Depression score	-0.05	-0.12	0.03

For peer review only - http://bmjopen.bmj.com/site/about/guidelines.xhtml

Supplementary Table S6 continued Coefficients (95% CI) from the Fixed Effects Model of grip strength (kg), ELSA waves 2-6

Smoking status (ref: never smoker)	Coefficients	Lower 95% CI	Upper 95% CI
Ex-Smoker	0.46	-0.35	1.27
Current smoker	0.76	-0.36	1.87
Date/Day orientation (ref: no correct answer)			
1 correct answer	0.70	-1.31	2.71
2 correct answers	**1.94**	0.17	3.71
3 correct answers	**1.93**	0.35	3.51
All correct answers	**1.87**	0.30	3.44
Memory test	0.02	-0.02	0.07
Number of observations	21835		
Adjusted R-squared	0.87		

Boldface indicates statistical significance (p<0.05)

For peer review only

For peer review only - http://bmjopen.bmj.com/site/about/guidelines.xhtml

Supplementary Table S7 Coefficients (95% CI) from the Fixed Effects multinomial model of chair stand outcomes, ELSA waves 2-6. Coefficients are log odds of not completing the test or taking longer than the median time to complete the test relative to using completing the chair stand test faster than the median time

	**Did not complete chair stand test**			**Took longer to complete 5/10 chair rises**		
Intercept	**Log odds**	**Lower 95% CI**	**Upper 95% CI**	**Log odds**	**Lower 95% CI**	**Upper 95% CI**
Age centered (linear term)	**0.09**	0.06	0.11	**0.10**	0.09	0.12
Age (quadratic term)	**0.004**	0.003	0.006	**0.002**	0.001	0.003
Frequency of public transport use(ref: every day or nearly every day)						
two or three times a week	0.00	-0.38	0.39	-0.01	-0.31	0.30
once a week	0.10	-0.34	0.53	-0.12	-0.46	0.21
did not use because no need	0.31	-0.12	0.75	0.15	-0.18	0.47
did not use because health problems	**1.28**	0.63	1.92	0.43	-0.16	1.02
did not use because structural reasons	**0.45**	0.02	0.89	0.05	-0.27	0.38
Wealth quintiles (ref: poorest quintile)						
Quintile 2	-0.39	-0.92	0.15	-0.29	-0.73	0.15
Quintile 3	-0.12	-0.70	0.47	-0.42	-0.89	0.04
Quintile 4	-0.32	-0.95	0.31	-0.46	-0.94	0.03
Richest quintile	-0.24	-0.94	0.46	-0.42	-0.93	0.10
Access to car/van (ref: yes access)						
No access to car/van	-0.20	-0.58	0.19	-0.28	-0.62	0.05
Employment status (ref: employed)						
Retired	**0.50**	0.16	0.84	0.05	-0.16	0.26
Other	**0.41**	0.01	0.81	0.09	-0.18	0.37
Social class (ref: Managerial & Prof)						
Intermediate occupations	0.78	-0.13	1.70	-0.08	-0.67	0.50
Self-employed	0.44	-0.42	1.29	0.19	-0.37	0.74
Lower supervisory & technical	-0.14	-1.19	0.90	-0.25	-0.97	0.46
Semi-routine & routine	0.16	-0.66	0.99	0.48	-0.05	1.01

For peer review only

Supplementary Table S7 continued Coefficients (95% CI) from the Fixed Effects multinomial model of chair stand outcomes, ELSA waves 2-6. Coefficients are log odds of not completing the test or taking longer than the median time to complete the test relative to using completing the chair stand test faster than the median time

	**Did not complete chair stand test**			**Took longer to complete 5/10 chair rises**		
Urban/Rural (ref: Urban)	**Log odds**	**Lower 95% CI**	**Upper 95% CI**	**Log odds**	**Lower 95% CI**	**Upper 95% CI**
Town & Fringe	0.09	-0.85	1.03	0.26	-0.40	0.92
Village	-0.69	-1.63	0.26	0.19	-0.55	0.93
Hamlet/Isolated	**1.54**	0.03	3.05	0.47	-0.49	1.43
Mobility difficulties (ref: none)						
1-3 difficulties	0.10	-0.15	0.34	0.11	-0.05	0.26
4+ difficulties	**0.83**	0.46	1.19	**0.37**	0.10	0.63
Activities of Daily living impairments (ref: none)						
At least one ADL impairment	**0.46**	0.18	0.73	**0.06**	-0.17	0.29
Physical Activity levels (ref: sedentary)						
Low	-0.14	-0.63	0.35	0.20	-0.25	0.66
Moderate	-0.48	-0.98	0.01	-0.09	-0.55	0.36
High	-0.21	-0.76	0.33	-0.11	-0.58	0.37
Marital status (ref: married)						
Separated/Divorced	-0.38	-1.22	0.46	-0.38	-1.00	0.25
Widowed	-0.55	-1.37	0.28	-0.37	-0.97	0.23
Never married	-0.31	-1.93	1.30	-1.22	-2.45	0.01
Cohabitation (ref: not living with partner)						
Living with partner	-0.66	-1.45	0.13	-0.29	-0.85	0.27
CESD- Depression score	**0.07**	0.02	0.13	0.01	-0.04	0.05
Smoking status (ref: never smoker)						
Ex-Smoker	-0.30	-0.99	0.39	0.15	-0.35	0.66
Current smoker	-0.58	-1.43	0.27	-0.01	-0.63	0.61

For peer review only

Supplementary Table S7 continued Coefficients (95% CI) from the Fixed Effects multinomial model of chair stand outcomes, ELSA waves 2-6. Coefficients are log odds of not completing the test or taking longer than the median time to complete the test relative to using completing the chair stand test faster than the median time

	**Did not complete chair stand test**			**Took longer to complete 5/10 chair rises**		
Date/Day orientation (ref: no correct answer)	**Log odds**	**Lower 95% CI**	**Upper 95% CI**	**Log odds**	**Lower 95% CI**	**Upper 95% CI**
1 correct answer	0.01	-2.15	2.17	-0.02	-1.72	1.67
2 correct answers	-0.89	-2.59	0.80	-0.01	-1.43	1.40
3 correct answers	-0.58	-2.19	1.03	0.34	-1.01	1.69
All correct answers	-0.68	-2.28	0.92	0.21	-1.14	1.55
Memory test	-0.03	-0.06	0.01	0.00	-0.03	0.02
Number of observations	12463					
Adjusted R-squared	0.10					

Boldface indicates statistical significance (p<0.05)

For peer review only - http://bmjopen.bmj.com/site/about/guidelines.xhtml

For peer review only

Supplementary Table S8 Coefficients (95% CI) from the Fixed Effects multinomial model of physical activity, ELSA waves 2-6. Coefficients are log odds of sedentary/low/moderate physical activity, relative to being in the high physical activity category

	**Sedentary physical activity**			**Low physical activity**			**Moderate physical activity**		
	**Log odds**	**Lower 95% CI**	**Upper 95% CI**	**Log odds**	**Lower 95% CI**	**Upper 95% CI**	**Log odds**	**Lower 95% CI**	**Upper 95% CI**
Age centered (linear term)	**0.17**	0.11	0.22	**0.10**	0.06	0.13	0.02	-0.01	0.05
Age (quadratic term)	**0.006**	0.004	0.007	**0.005**	0.004	0.006	**0.002**	0.001	0.003
Frequency of public transport use(ref: every day or nearly every day)									
two or three times a week	-0.18	-0.64	0.28	**-0.33**	-0.62	-0.03	-0.15	-0.40	0.10
once a week	0.06	-0.47	0.60	-0.24	-0.57	0.08	-0.10	-0.37	0.17
did not use because no need	-0.22	-0.75	0.30	-0.16	-0.49	0.16	-0.08	-0.35	0.19
did not use because health problems	1.37	0.65	2.09	0.81	0.23	1.39	0.39	-0.17	0.94
did not use because structural reasons	0.10	-0.43	0.63	-0.10	-0.42	0.22	-0.08	-0.35	0.19
Interaction between Age (linear term) and Frequency of public transport use (ref: Nearly/Every day use)									
Age*two or three times a week	0.04	-0.01	0.08	0.03	-0.001	0.07	0.03	-0.001	0.06
Age*once a week	0.04	-0.01	0.09	0.02	-0.02	0.06	0.02	-0.01	0.05
Age*did not use because no need	**0.06**	0.01	0.11	0.02	-0.01	0.06	**0.04**	0.01	0.07
Age*did not use because health problems	0.06	-0.003	0.13	0.02	-0.03	0.08	0.02	-0.04	0.07
Age*did not use because structural reasons	**0.06**	0.01	0.11	0.03	-0.01	0.06	**0.04**	0.01	0.07
Wealth quintiles (ref: poorest quintile)									
Quintile 2	0.21	-0.36	0.78	0.34	-0.03	0.72	**0.35**	0.04	0.66
Quintile 3	-0.02	-0.64	0.60	0.21	-0.20	0.61	0.30	-0.04	0.63
Quintile 4	-0.28	-0.95	0.39	0.02	-0.40	0.45	0.26	-0.09	0.61
Richest quintile	-0.66	-1.45	0.13	0.03	-0.43	0.49	0.29	-0.08	0.67
Access to car/van (ref: yes access)									
No access to car/van	**0.40**	0.05	0.76	0.08	-0.21	0.38	-0.01	-0.28	0.25
Employment status (ref: employed)									
Retired	**0.79**	0.24	1.33	0.06	-0.15	0.28	0.01	-0.15	0.16
Other	**0.73**	0.16	1.30	0.08	-0.18	0.34	0.02	-0.18	0.23

For peer review only - http://bmjopen.bmj.com/site/about/guidelines.xhtml

Supplementary Table S8 continued Coefficients (95% CI) from the Fixed Effects multinomial model of physical activity, ELSA waves 2-6. Coefficients are log odds of sedentary/low/moderate physical activity, relative to being in the high physical activity category

	**Sedentary physical activity**			**Low physical activity**			**Moderate physical activity**		
Social class (ref: Managerial & Prof)	**Log odds**	**Lower 95% CI**	**Upper 95% CI**	**Log odds**	**Lower 95% CI**	**Upper 95% CI**	**Log odds**	**Lower 95% CI**	**Upper 95% CI**
Intermediate occupations	-0.97	-2.46	0.52	-0.53	-1.09	0.02	-0.21	-0.60	0.17
Self-employed	0.09	-1.37	1.55	**-0.79**	-1.36	-0.22	**-0.47**	-0.85	-0.09
Lower supervisory & technical	**-1.85**	-3.52	-0.18	-0.65	-1.36	0.07	-0.06	-0.51	0.40
Semi-routine & routine	-1.23	-2.67	0.21	-0.35	-0.87	0.18	-0.19	-0.54	0.16
Urban/Rural (ref: Urban)									
Town & Fringe	-0.62	-1.81	0.57	0.41	-0.21	1.04	0.28	-0.20	0.76
Village	0.23	-1.22	1.69	0.12	-0.53	0.76	-0.42	-0.91	0.08
Hamlet/Isolated	-0.01	-1.77	1.76	-0.22	-1.16	0.73	-0.35	-1.09	0.39
Mobility difficulties (ref: none)									
1-3 difficulties	-0.05	-0.36	0.26	**0.25**	0.10	0.39	**0.15**	0.03	0.26
4+ difficulties	**0.86**	0.46	1.26	**0.97**	0.71	1.23	**0.52**	0.29	0.75
Activities of Daily living impairments (ref: none)									
At least one ADL impairment	**0.63**	0.36	0.90	**0.27**	0.07	0.48	0.07	-0.11	0.26
Marital status (ref: married)									
Separated/Divorced	0.48	-0.35	1.31	0.25	-0.29	0.79	0.23	-0.20	0.65
Widowed	0.12	-0.74	0.98	-0.02	-0.58	0.55	0.12	-0.34	0.59
Never married	1.07	-0.50	2.63	-0.06	-1.01	0.89	-0.05	-0.78	0.68
Cohabitation (ref: not living with partner)									
Living with partner	0.62	-0.23	1.47	0.45	-0.08	0.98	0.23	-0.19	0.65
CESD- Depression score	**0.19**	0.14	0.25	**0.14**	0.10	0.18	**0.06**	0.03	0.09
Smoking status (ref: never smoker)									
Ex-Smoker	-0.47	-1.31	0.36	-0.33	-0.81	0.15	-0.28	-0.67	0.11
Current smoker	-0.63	-1.60	0.34	-0.57	-1.16	0.01	-0.28	-0.76	0.20

For peer review only

For peer review only - http://bmjopen.bmj.com/site/about/guidelines.xhtml

Supplementary Table S8 continued Coefficients (95% CI) from the Fixed Effects multinomial model of physical activity, ELSA waves 2-6. Coefficients are log odds of sedentary/low/moderate physical activity, relative to being in the high physical activity category

	**Sedentary physical activity**			**Low physical activity**			**Moderate physical activity**		
Date/Day orientation (ref: no correct answer)	**Log odds**	**Lower 95% CI**	**Upper 95% CI**	**Log odds**	**Lower 95% CI**	**Upper 95% CI**	**Log odds**	**Lower 95% CI**	**Upper 95% CI**
1 correct answer	0.26	-1.42	1.94	-0.36	-1.91	1.18	-0.63	-2.12	0.85
2 correct answers	-0.70	-2.20	0.79	-0.63	-2.02	0.76	-0.70	-2.03	0.63
3 correct answers	-0.91	-2.34	0.52	-0.55	-1.88	0.78	-0.57	-1.84	0.71
All correct answers	-1.05	-2.48	0.37	-0.62	-1.95	0.70	-0.62	-1.89	0.65
Memory test	**-0.05**	-0.09	-0.02	**-0.03**	-0.05	-0.004	-0.02	-0.03	0.003
Number of observations	32,276								
Adjusted R-squared	0.06								

Boldface indicates statistical significance (p<0.05)

For peer review only

For peer review only - http://bmjopen.bmj.com/site/about/guidelines.xhtml

Supplementary Table S9 Coefficients (95% CI) from the Multilevel Growth Curve Model of walking speed (m/s), ELSA waves 2-6: Only participants with 3 or more waves of data

**Fixed Part**	**Coefficients**	**Lower 95% CI**	**Upper 95% CI**
Intercept	**0.80**	0.75	0.86
Age centered (linear term)	**-0.01**	-0.01	-0.01
Age (quadratic term)	**-0.0002**	-0.0003	-0.0002
Frequency of public transport use(ref: every day or nearly every day)			
two or three times a week	-0.01	-0.02	0.0003
once a week	**-0.01**	-0.03	-0.001
did not use because no need	**-0.02**	-0.03	-0.003
did not use because health problems	**-0.09**	-0.11	-0.08
did not use because structural reasons	**-0.011**	-0.023	0.001
Interaction between Age (linear term) and Frequency of public transport use (ref: nearly/every day use)			
Age*two or three times a week	-0.0003	-0.0018	0.0013
Age*once a week	-0.0016	-0.0032	0.00003
Age*did not use because no need	-0.0012	-0.0027	0.0004
Age*did not use because health problems	0.0005	-0.0014	0.0024
Age*did not use because structural reasons	**-0.0019**	-0.0034	-0.0004
Sex (ref: men)			
Women	-0.03	-0.03	-0.02
Wealth quintiles (ref: poorest quintile)			
Quintile 2	**0.02**	0.01	0.03
Quintile 3	**0.03**	0.02	0.04
Quintile 4	**0.05**	0.04	0.07
Richest quintile	**0.08**	0.06	0.09
Access to car/van (ref: yes access)			
No access to car/van	**-0.02**	-0.03	-0.01
Employment status (ref: employed)			
Retired	-0.01	-0.02	0.002
Other	**-0.02**	-0.03	-0.003
Social class (ref: Managerial & Prof)			
Intermediate occupations	**-0.02**	-0.04	-0.01
Self-employed	**-0.02**	-0.04	-0.01
Lower supervisory & technical	**-0.04**	-0.05	-0.02
Semi-routine & routine	**-0.05**	-0.06	-0.04
Urban/Rural (ref: Urban)			
Town & Fringe	0.01	-0.004	0.02
Village	**0.02**	0.01	0.03
Hamlet/Isolated	**0.02**	0.00	0.04
Mobility difficulties (ref: none)			
1-3 difficulties	**-0.03**	-0.04	-0.03
4+ difficulties	**-0.11**	-0.12	-0.10
Activities of Daily living impairments (ref: none)			
At least one ADL impairment	**-0.05**	-0.06	-0.04

For peer review only

Supplementary Table S9 continued Coefficients (95% CI) from the Multilevel Growth Curve Model of walking speed (m/s), ELSA waves 2-6: Only participants with 3 or more waves of data

Physical Activity levels (ref: sedentary)	**Coefficients**	**Lower 95% CI**	**Upper 95% CI**
Low	**0.04**	0.03	0.06
Moderate	**0.09**	0.07	0.10
High	**0.11**	0.09	0.12
Marital status (ref: married)			
Separated/Divorced	**0.03**	0.003	0.05
Widowed	0.02	-0.01	0.04
Never married	-0.004	-0.03	0.03
Cohabitation (ref: not living with partner)			
Living with partner	0.02	-0.002	0.04
CESD- Depression score	**-0.01**	-0.01	-0.01
Smoking status (ref: never smoker)			
Ex-Smoker	**-0.01**	-0.02	-0.002
Current smoker	**-0.03**	-0.04	-0.01
Date/Day orientation (ref: no correct answer)			
1 correct answer	0.02	-0.04	0.08
2 correct answers	0.04	-0.01	0.09
3 correct answers	0.05	-0.001	0.09
All correct answers	**0.05**	0.004	0.10
Memory test	**0.004**	0.003	0.01
**Random Part**			
Level 2 (Individual)			
Intercept variance	0.02		
Age centered (linear term) variance	0.00003		
Covariance of intercept and age centered	-0.0003		
Level 1 (wave)			
Intercept	0.02		
Number of observations (level 1)	5547		
Number of clusters (level 2)	21573		
-2*Log Likelihood	-11874.03		

Boldface indicates statistical significance ($p<0.05$)

For peer review only

For peer review only - http://bmjopen.bmj.com/site/about/guidelines.xhtml

STROBE 2007 (v4) Statement—Checklist of items that should be included in reports of *cohort studies*

Section/Topic	Item #	Recommendation	Reported on page #
**Title and abstract**	1	(*a*) Indicate the study's design with a commonly used term in the title or the abstract	2
		(*b*) Provide in the abstract an informative and balanced summary of what was done and what was found	2
**Introduction**			
Background/rationale	2	Explain the scientific background and rationale for the investigation being reported	4-5
Objectives	3	State specific objectives, including any prespecified hypotheses	5
**Methods**			
Study design	4	Present key elements of study design early in the paper	6
Setting	5	Describe the setting, locations, and relevant dates, including periods of recruitment, exposure, follow-up, and data collection	6
Participants	6	(*a*) Give the eligibility criteria, and the sources and methods of selection of participants. Describe methods of follow-up	6-9
		(*b*) For matched studies, give matching criteria and number of exposed and unexposed	NA
Variables	7	Clearly define all outcomes, exposures, predictors, potential confounders, and effect modifiers. Give diagnostic criteria, if applicable	6-7
Data sources/ measurement	8*	For each variable of interest, give sources of data and details of methods of assessment (measurement). Describe comparability of assessment methods if there is more than one group	6-7
Bias	9	Describe any efforts to address potential sources of bias	8-9
Study size	10	Explain how the study size was arrived at	8
Quantitative variables	11	Explain how quantitative variables were handled in the analyses. If applicable, describe which groupings were chosen and why	6-7
Statistical methods	12	(*a*) Describe all statistical methods, including those used to control for confounding	8-9
		(*b*) Describe any methods used to examine subgroups and interactions	8
		(*c*) Explain how missing data were addressed	8-9
		(*d*) If applicable, explain how loss to follow-up was addressed	8-9
		(*e*) Describe any sensitivity analyses	NA
**Results**			

For peer review only

Participants	13*	(a) Report numbers of individuals at each stage of study—eg numbers potentially eligible, examined for eligibility, confirmed eligible, included in the study, completing follow-up, and analysed	11
		(b) Give reasons for non-participation at each stage	8-9
		(c) Consider use of a flow diagram	NA
Descriptive data	14*	(a) Give characteristics of study participants (eg demographic, clinical, social) and information on exposures and potential confounders	9-10
		(b) Indicate number of participants with missing data for each variable of interest	9-10
		(c) Summarise follow-up time (eg, average and total amount)	NA
Outcome data	15*	Report numbers of outcome events or summary measures over time	NA
Main results	16	(*a*) Give unadjusted estimates and, if applicable, confounder-adjusted estimates and their precision (eg, 95% confidence interval). Make clear which confounders were adjusted for and why they were included	12
		(*b*) Report category boundaries when continuous variables were categorized	12
		(*c*) If relevant, consider translating estimates of relative risk into absolute risk for a meaningful time period	12
Other analyses	17	Report other analyses done—eg analyses of subgroups and interactions, and sensitivity analyses	Supplementary materials
**Discussion**			
Key results	18	Summarise key results with reference to study objectives	14
**Limitations**			
Interpretation	20	Give a cautious overall interpretation of results considering objectives, limitations, multiplicity of analyses, results from similar studies, and other relevant evidence	14-16
Generalisability	21	Discuss the generalisability (external validity) of the study results	15
**Other information**			
Funding	22	Give the source of funding and the role of the funders for the present study and, if applicable, for the original study on which the present article is based	17

*Give information separately for cases and controls in case-control studies and, if applicable, for exposed and unexposed groups in cohort and cross-sectional studies.

Note: An Explanation and Elaboration article discusses each checklist item and gives methodological background and published examples of transparent reporting. The STROBE checklist is best used in conjunction with this article (freely available on the Web sites of PLoS Medicine at http://www.plosmedicine.org/, Annals of Internal Medicine at http://www.annals.org/, and Epidemiology at http://www.epidem.com/). Information on the STROBE Initiative is available at www.strobe-statement.org.

For peer review only

BMJ Open

Does public transport use prevent declines in walking speed among older adults living in England? A prospective cohort study

Journal:	*BMJ Open*
Manuscript ID	bmjopen-2017-017702.R2
Article Type:	Research
Date Submitted by the Author:	04-Aug-2017
Complete List of Authors:	Rouxel, Patrick; University College London Institute of Education, Social Science<br>Webb, Elizabeth; University College London, International Centre for Lifecourse Studies, Epidemiology and Public Health<br>Chandola, Tarani; University of Manchester, The Cathie Mash Centre for Census and Survey Research
**Primary Subject Heading**:	Epidemiology
Secondary Subject Heading:	Public health
Keywords:	public transport, ageing, walking speed, physical activity

SCHOLARONE™
Manuscripts

For peer review only

Does public transport use prevent declines in walking speed among older adults living in England? A prospective cohort study

Patrick Rouxel [1], Elizabeth Webb [2] and Tarani Chandola [3]

[1]CLOSER, Department of Social Science, University College London Institute of Education, London, UK, ORCID: 0000-0003-0330-554X, [2] International Centre for Lifecourse Studies, Department of Epidemiology and Public Health, University College London, London, UK, ORCID: 0000-0001-6106-5367 and [3]CMIST and Social Statistics, University of Manchester, UK, ORCID: 0000-0002-1864-3413

Corresponding author

Patrick Rouxel, CLOSER, Department of Social Science, University College London Institute of Education, 55-59 Gordon Square, WC1H 0NU, London, UK. E-mail: patrick.rouxel@ucl.ac.uk; phone: +44 7527565347

Word count 4,111

For peer review only

Abstract

Objectives: Although there is some evidence that public transport use confers public health benefits, the evidence is limited by cross-sectional study designs and health-related confounding factors. This study examines the effect of public transport use on changes in walking speed among older adults living in England, comparing frequent users of public transport to their peers who did not use public transport because of structural barriers (poor public transport infrastructure), or through choice.

Design: Prospective cohort study.

Setting: England, UK

Participants: Older adults aged 60 or older eligible for the walking speed test. 6,246 individuals at wave 2 (2004-05); 5,909 individuals at wave 3 (2006-07); 7,321 individuals at wave 4 (2008-09); 7,535 individuals at wave 5 (2010-11); and 7,664 individuals at wave 6 (2012-13) of the English Longitudinal Study of Ageing.

Main outcome measure: The walking speed was estimated from the time taken to walk 2.4 metres. Fixed effects models and growth curve models were used to examine the associations between public transport use and walking speed.

Results: Older adults who did not use public transport through choice or because of structural reasons had slower walking speeds [-0.02 m/s (95%CI -0.03,-0.003) and -0.02 m/s (95%CI -0.03,-0.01), respectively] and took an extra 0.07 seconds to walk 2.4 meters compared to their peers who used public transport frequently. The age-related trajectories of decline in walking speed were slower for frequent users of public transport compared to non-users.

Conclusions: Frequent use of public transport may prevent age-related decline in physical capability by promoting physical activity and lower limb muscle strength among older adults. The association between public transport use and slower decline in walking speed among older adults is unlikely to be confounded by health related selection factors. Improving access to good quality public transport could improve the health of older adults.

Strengths and limitations of the study

- Previous cross-sectional research on the protective role of public transport use in relation to age related functional declines may have been biased by health selection processes as people age.
- Older adults with deteriorating health are less likely to use public transport and their poor health could determine their functional decline, rather than their lack of public transport use.

For peer review only - http://bmjopen.bmj.com/site/about/guidelines.xhtml

- This longitudinal study of over 7,000 adults living in England suggests that the inference that frequent use of public transport may prevent age-related decline in physical capability is robust to such potential biases.
- While this is not a causal analysis, we have controlled for within- and between-person factors that could bias the association between public transport use and walking speed decline.
- Given the current context of cuts in public transport availability in England, this research suggests that such cuts may result in faster declines in physical functioning as people age.

For peer review only

For peer review only - http://bmjopen.bmj.com/site/about/guidelines.xhtml

Introduction

Declines in walking speed and grip strength are markers of ageing and are associated with all-cause mortality [1] and poorer health in older populations. [2] Maintaining physical capability is a prerequisite for older people to engage independently in many social activities [3] and for reducing social exclusion. [4 5]

Functional capacity and muscle strength are key dimensions of sarcopenia. [6] Compelling evidence supports the efficacy of physical activity in maintaining muscle mass, strength and function in older adults. [7 8] Older adults may add regular physical activity into daily life by walking and maintaining balance on moving vehicles such as buses or trains. [7] Public transport related physical activity was associated with a larger reduction in mortality for older adults (above 70 years) compared to younger adults. [9] Public transport users are more physically active than non-users of public transport. [10-12]

Disability is not the only barrier to the use of public transport for many older people. Other barriers include the costs and poor quality of public transport. [5 13 14] These barriers, in turn, suppress leisure, social interactions and shopping activities. [13 15] Accessible, affordable and convenient transport is important to enable older people to access services and amenities. It is particularly important to consider such barriers to public transport use given the current context of cuts to local bus services in England. [16]

Despite concessionary bus passes in the UK offering free bus travel to those over the State Pension Age, in England a third of older adults aged over 65 never use public transport, whilst another third use it very infrequently. [17] Evidence from the English Longitudinal Study of Ageing (ELSA) [18 19] and other studies [5 20] showed that free bus travel for older people was associated with increased active travel and raised physical activity. A review has reported between 8 and 33 minutes of additional physical activity by walking through use of public transport, although most of the studies included were not focused on older adults. [11] Although walking speed declines with age, [21 22] there is some cross-sectional evidence that older women with a free bus pass use public transport more often and have faster walking speeds than those who do not hold a bus pass. [18] Moreover, public transport use is associated with lower levels of obesity and may have a protective effect against becoming obese. [19] A recent longitudinal study on the use of active or public transport versus cars to commute to work showed that people who changed from active or public commuting to car commuting had an increase in BMI of

For peer review only - http://bmjopen.bmj.com/site/about/guidelines.xhtml

0.3 kg/m^2 while those who changed from car commuting to active or public commuting had a decrease in BMI of 0.3 kg/m^2. [23]

However, the key limitation in the existing evidence is that negative health selection is not taken into account. Older adults with deteriorating health may be less likely to use public transport and their poor health could determine the decline in walking speed, rather than their lack of public transport use. There is also a lack of analyses on different reasons for not using public transport. Older adults may not use public transport because of health problems and disabilities [3 24] or because reliable public transport is not available, [13] or because they prefer using their own vehicles. [25] Separating out these reasons for not using public transport is important: if a negative effect of not using public transport on walking speed is observed among older adults who do not use public transport due to structural reasons (poor public transport infrastructure) or through choice, this suggests the observed association between public transport use and walking speed is not confounded by health.

An additional test of the specificity of the public transport-walking speed association is whether similar associations are observed between public transport use and upper body strength as measured by grip strength. As moderate to vigorous physical activity, including public transport use, does not increase grip strength, [26] any association between public transport use and grip strength could be caused by confounding factors such as stronger people being selected into using public transport. Moreover, if public transport use affects walking speed, the mechanisms are likely to be through walking related physical activity and slower declines in lower limb strength.

Our study will address the following research questions:

RQ1: Do older adults who frequently use public transport have faster walking speeds than those who do not use public transport for structural reasons or through choice? Is some of the association between public transport use and walking speed going through the mechanisms of physical activity and lower limb strength?

RQ2: Is the association of public transport use with muscle function deficit specific to lower limb muscle strength? Does public transport use also predict stronger grip strength?

RQ3: Are declines of walking speed with age slower for older adults who use public transport often, compared to their peers who do not use public transport for structural reasons or through choice?

For peer review only - http://bmjopen.bmj.com/site/about/guidelines.xhtml

Methods

Data

The data come from waves 2 to 6 of the English Longitudinal Study of Ageing (ELSA), where individuals aged 50 and over living in private households in England were followed and re-interviewed every two years. [27] The ELSA sample was refreshed at waves 3, 4, and 6 to ensure the sample remained representative of the population aged 50 and older. The National Research Ethics Service approved the study, and all participants gave their informed consent. We used data from older adults aged 60 or older - those who were eligible for the walking speed test - consisting of 6,246 individuals at wave 2 (2004-05); 5,909 individuals at wave 3 (2006-07); 7,321 individuals at wave 4 (2008-09); 7,535 individuals at wave 5 (2010-11); and 7,664 individuals at wave 6 (2012-13). Data from wave 1 were omitted, since the reasons why people did not use public transport were not asked. Data were collected though face-to-face interview and a self-completion questionnaire. In addition, there was a nurse visit at waves 2, 4 and 6. The ELSA data and documentation are publicly available from the UK Data Service. (http://www.esds.ac.uk/findingData/snDescription.asp?sn=5050).

Variables

Walking speed (m/s) was measured among participants aged 60 and older at every ELSA wave. They were asked to twice walk a distance of 8 feet (2.4m) at their usual pace. Walking aids were permitted. We used the mean walking speed based on the two timings.

Grip strength (kg) was used as an indicator of upper body strength. Participants were asked to squeeze a hand-held dynamometer up to three times with each hand in waves 2, 4 and 6. We used the mean of the three measurements for the dominant hand

Chair stands were used as an indicator of lower limb muscle strength. Respondents at waves 2, 4 and 6 aged under 70 were asked to do 10 chair stands and those aged 70+ were asked to do 5 chair stands. This was grouped into people who could not complete either 5 or 10 chair stands (including those who could not complete a single chair stand), those who took longer than the median time to complete 5/10 chair stands, and those who completed the task in less than the median time.

For peer review only

Participants were asked how often they used public transport. In addition, those who rarely or never used public transport were asked to provide the reasons of not using public transport more often. We then derived the frequency variable as follows: every day/nearly every day; 2-3 times a week; once a week; no use because did not need to (or in other words through "choice"); no use because of health problems; no use because of structural reasons. Structural reasons, for these purposes, include: not convenient, does not go where they want, infrequent, unreliable, too expensive, too dirty, fear of crime. At wave 2, the frequency of use variable had different responses which we mapped onto the later wave responses as follows: a lot=nearly every day; quite often=2-3 times a week; sometimes=once a week; rarely/never=no use. [19]

Covariates

A quadratic term for continuous age was specified to characterize non-linear age effects.

Other covariates included gender; marital status (married; divorced/separated; widowed; never married), cohabiting status (currently living with a partner or not); urban/rural areas (urban; town; village; hamlet) - the rural–urban definition is applied to the Census Output Area that each individual lives in; quintiles of non-pension wealth; access to car (whether driver or passenger); employment status (employed; retired; other); National Statistics Socio-economic Classification (NS-SEC) social class; smoking (never; former; current). Mobility difficulties were assessed by asking participants whether they had difficulties with 10 common functions (e.g. walking 100 yards; climbing several flights of stairs without resting; climbing one flight of stairs without resting; lifting or carrying weights). We derived a variable with three categories: no difficulties; 1-3 difficulties, 4 or more difficulties. The number of functional limitations in Activities of Daily Living (ADLs) provides an indication of disability. The ADLs scale [28] comprises problems with dressing, walking, bathing, eating (such as cutting up food), getting out of bed, and using the toilet. We derived a binary variable no limitations in ADLs; at least one limitation in ADLs. Depressive symptoms were measured using the eight-item version of the Center for Epidemiologic Study Depression (CES-D) scale. [29] Participants were asked how often they participated in mild, moderate or vigorous physical activity including participation in occupations that involve physical work. Based on their responses, they were classified into the following categories: sedentary; low; moderate or high level of physical activity. [30] Cognitive function was assessed through two memory tests. Study participants were asked to recall a list of 10 words immediately after reading them and then again after a 5-minute delay. We computed an overall memory score (range, 0– 20) using both the immediate and delayed

For peer review only

recall results (between-test correlation coefficient = 0.70). Orientation in time (day, month, year and day of the week) is another test of memory.

Analysis

For RQs 1 and 2: Fixed effects linear and multinomial regression models were used to regress walking speed, physical activity, chair stands and grip strength on the frequency of using public transport. These models investigate the effects of within individual (time varying) changes in public transport use on changes in walking speed, physical activity, lower limb and upper body muscle strength, taking into account other time varying covariates. We also used fixed effects multinomial logit models to examine whether the direction of association could be in the opposite direction, i.e. whether changes in the use/non-use of public transport (for different reasons) were predicted by changes in walking speed and other covariates. We estimated these models in Stata 14.0.

For RQ3: We used multilevel (random effects) growth curve models to estimate age-related trajectories of walking speed for different categories of public transport use. These models can be used to describe how different trajectories of walking speed change with age, by interacting age with the frequency of public transport use. Individual trajectories (at level 2) of walking speed (at level 1) are estimated, with a random slope of age (at level 2) characterizing differences in individual trajectories of walking speed. As participants with a single walking speed measurement can contribute to the overall growth curve model, we additionally estimated age-related trajectories of walking speed for those participants with at least three waves of walking speed measurements. We estimated these models in MLwiN 2.1.

Missing data

For the walking speed analyses, there are 29,894 observations of walking speed between waves 2 to 6, which reduced to 27,525 observations in the statistical models due to missing data in the covariates. Attrition between waves was not strictly monotonic- some ELSA participants returned to the study after missing one or two waves of data collection. Hence rather than analysing factors only associated with attrition, we analysed factors associated with missing data in any of the independent and dependent variables and covariates, conditional on observation of a participant's walking speed at baseline.

For peer review only

Analyses of the pattern of missingness in the cohort with a baseline walking speed measurement revealed that 33% of that cohort had subsequently dropped out by wave 6, 16% were missing a walking speed measure at wave 6, 5% were missing both a walking speed and wealth measurement, and 3% were missing a wealth measurement. Other covariates accounted for less than 2% of the missing data.

We modelled the odds of having any missing data (conditional on having a baseline walking speed measurement) as shown in Supplementary Table S1. Women and older participants were less likely to have any missing data, especially older women participants. Participants who did not use public transport because of health problems were more likely to be missing compared to those who used public transport frequently, but those who did not use public transport for other reasons were not more likely to be missing. Socio-economic disadvantage (not having access to a car/van and being in the semi-routine and routine occupational class) was associated with higher odds of being missing, as was having a disability, low physical activity, low memory scores and higher levels of depressive symptoms.

The ELSA study team provides longitudinal weights for the core ELSA members present at each wave from the first wave. We did not use these for our analyses as, since they are not available for the ELSA refreshment sample members, using the longitudinal weights would have reduced our sample size by more than half. Instead, we used the wave specific cross-sectional weights in both the fixed effects and multilevel growth curve models, in order to make our analyses representative of non-institutionalized older adults living in England. These cross-sectional weights take into account the greater likelihood of non-response by participants who have poorer health and who are more socio-economically disadvantaged.[31]

Results

Table 1 shows the distribution of all the variables used in the analysis. The mean walking speed was 0.85 m/s and the mean age was 70.5. 34% of ELSA respondents across waves 2-6 reported taking public transport at least once a week, while 33% reported not using public transport because of structural reasons. Most of the sample (73%) lived in urban areas, and almost 60% reported at least one difficulty with mobility.

For peer review only

Table 1 Distribution (percentage/mean) of all the variables in the analysis, observations (n) across ELSA waves 2-6 among respondents with walking speed data

Variables	%/Mean (sd)	n obs across 6 waves	Variables	%/Mean (sd)	n obs across 6 waves
**Walking speed (m/s)**	0.9 (0.3)	29894	**Urban/rural**		
**Chair stands**			urban	72.6%	21,679
could not complete test	17.5%	2780	town	12.6%	3,771
completed test slower	45.8%	7288	village	10.8%	3,213
completed test faster	36.8%	5857	hamlet	4.0%	1,198
**Frequency of public transport use**			**Marital status**		
every day or nearly every day	8.5%	2,540	married	65.0%	19,429
two or three times a week	14.4%	4,300	separated/divorced	9.8%	2,923
once a week	11.2%	3,342	widowed	20.5%	6,117
no use: no need	27.9%	8,337	never married	4.8%	1,421
no use: health problems	5.5%	1,631	**Mobility difficulties**		
no use: structural reasons	32.6%	9,741	none	40.7%	12,155
**Age**	70.5 (7.7)	29,894	1 to 3	37.6%	11,233
**Gender**			4 or more	21.8%	6,502
male	45.9%	13,728	**Functional status (ADLS)**		
female	54.1%	16,166	no limitation	82.1%	24,538
**Wealth quintiles**			at least 1 limitation	17.9%	5,355
poorest	16.4%	4,660	**CES-D depression score**	1.3 (1.8)	29579
2nd	18.8%	5,327	**Physical activity**		
3rd	21.2%	5,999	sedentary	5.0%	1,503
4th	21.6%	6,135	low	26.1%	7,770
richest	22.0%	6,245	moderate	51.1%	15,236
**Employment status**			high	17.8%	5,321
employed	16.8%	5,010	**Smoking status**		
retired	74.0%	22,081	never smoked	36.6%	10,931
other	9.2%	2,748	ex-smoker	52.1%	15,551
**Social class**			current smoker	11.2%	3,351
managerial	32.2%	9,458	**Date/Day orientation**		
intermediate	14.0%	4,093	All dates/day incorrect	0.7%	198
self-employed	11.7%	3,434	3 incorrect	0.6%	192
lower supervisory	10.5%	3,083	2 incorrect	1.9%	579
semi-routine	31.6%	9,270	1 incorrect	17.9%	5357
**Cohabiting status**			All dates/day correct	78.8%	23548
living alone	33.2%	9,909	**Access to car**		
living with partner	66.9%	19,985	yes access to car	83.4%	24,944
**Memory test (n of words)**	9.9 (3.6)	29,833	no access to car	16.6%	4,950

For peer review only

Table 2 reports the mean walking speed by frequency of public transport use at waves 2-6. Those who did not use public transport due to health problems had the slowest walking speed, while those who did not use public transport because of structural reasons had the fastest walking speed. There was no pattern of slower walking speed in later waves due to refreshment samples which resulted in the mean age at wave 6 being younger than at wave 2.

Table 2 Weighted mean (95% CI) of walking speed (m/s) by frequency of public transport use at waves 2-6 of ELSA

	every day or nearly every day	two or three times a week	once a week	no use: no need	no use: health problems	no use: structural reasons
wave 2	0.79 (0.77, 0.81)	0.79 (0.77, 0.81)	0.85 (0.84, 0.87)	0.84 (0.83, 0.86)	0.45 (0.42, 0.47)	0.90 (0.89, 0.92)
n	652	695	1,071	1,475	333	1,252
wave 3	0.77 (0.75, 0.80)	0.79 (0.77, 0.80)	0.79 (0.76, 0.81)	0.84 (0.82, 0.85)	0.44 (0.42, 0.47)	0.90 (0.88, 0.91)
n	389	694	462	1,431	289	1,863
wave 4	0.78 (0.76, 0.81)	0.80 (0.78, 0.82)	0.85 (0.83, 0.88)	0.84 (0.83, 0.85)	0.47 (0.44, 0.5)	0.89 (0.88, 0.91)
n	499	956	556	1,584	295	2,157
wave 5	0.81 (0.78, 0.83)	0.84 (0.83, 0.86)	0.86 (0.84, 0.88)	0.88 (0.87, 0.89)	0.47 (0.44, 0.49)	0.91 (0.90, 0.93)
n	472	935	624	1,610	315	2,148
wave 6	0.81 (0.79, 0.84)	0.84 (0.83, 0.86)	0.84 (0.82, 0.87)	0.90 (0.88, 0.91)	0.49 (0.46, 0.51)	0.93 (0.91, 0.94)
n	450	906	547	1,884	346	1,881

Table 3 reports the results of the fixed effects and multilevel growth curve (random effects) models of walking speed- the full models are shown in online Supplementary Tables S2 and S3. In the fixed effects model, the linear and quadratic terms of age were negative, suggesting that as age increased, walking speed declined faster. Compared to the reference group who used public transport nearly every day, the coefficients for all the other frequency groups were negative, suggesting that using public transport nearly every day had a protective effect on walking speed. Those who did not use public transport because of health problems had the biggest decline in walking speed (-0.06 m/s), but those who did not use public transport because they did not need to, or because of structural reasons were also more likely to have a decline in walking speed (-0.02 m/s). A difference of 0.02 m/s is an extra 0.07 seconds taken to do the walking speed test. The interaction between age and frequency of use of public transport was not significant in the fixed effects model.

For peer review only

Table 3 Selected coefficients (95% CI) from the Fixed Effects and Growth Curve Models of walking speed (m/s), ELSA waves 2-6

	Fixed Effects Model	Growth Curve Model 1	Growth Curve Model 2
**Fixed Part**			
Intercept	**0.820 (0.756,0.884)**	**0.823 (0.779,0.866)**	**0.830 (0.791,0.87)**
Age centered (linear term)	**-0.008 (-0.01,-0.007)**	**-0.007 (-0.008,-0.006)**	**-0.006 (-0.007,-0.004)**
Age (quadratic term)	**-0.0003 (-0.0004,-0.0003)**	**-0.0002 (-0.0002,-0.0001)**	**-0.0002 (-0.0002,-0.0002)**
p, 2df	*<0.001*	*<0.001*	*<0.001*
Frequency of public transport use(ref: every day or nearly every day)			
two or three times a week	-0.012 (-0.024,0.0001)	-0.006 (-0.016,0.003)	-0.005 (-0.018,0.008)
once a week	**-0.020 (-0.034,-0.005)**	**-0.011 (-0.022,-0.0002)**	-0.006 (-0.019,0.008)
no use: no need	**-0.018 (-0.032,-0.003)**	**-0.011 (-0.022,-0.001)**	-0.005 (-0.018,0.008)
no use: health problems	**-0.058 (-0.075,-0.040)**	**-0.090 (-0.104,-0.077)**	**-0.115 (-0.135,-0.095)**
no use: structural reasons	**-0.020 (-0.035,-0.006)**	**-0.009 (-0.019,0.002)**	-0.002 (-0.014,0.011)
p, 5df		*<0.001*	*<0.001*
Interaction between Age (linear term) and Frequency of public transport use			
Age*two or three times a week			-0.0003 (-0.002,0.001)
Age*once a week			-0.001 (-0.002,0.0004)
Age*no use: no need			**-0.001 (-0.002,-0.00002)**
Age*no use: health problems			**0.002 (0.0001,0.003)**
Age*no use: structural reasons			**-0.001 (-0.003,-0.0002)**
p, 5df			*<0.001*
**Random Part**			
Level 2 (Individual)			
Intercept variance		0.0238	0.0238
Age centered (linear term) variance		0.00002	0.00002
Covariance of intercept and age centered		-0.0004	-0.0004
Level 1 (wave)			
Intercept		0.020	0.020
N observations (level 1)	27509	27509	27509
N clusters (level 2)	9656	9656	9656
Goodness of fit	Adj R-sq: 0.7273	Deviance: -13719.59	Deviance: -13746.54

Boldface indicates statistical significance (p<0.05)

We additionally examined whether the association between public transport use and walking speed decreased when taking into account lower limb muscle strength (chair stands) and the interaction between physical activity and age (Supplementary Table S4). Similar to the coefficients reported in Table 3, the coefficients in the model without controlling for these potential mechanisms for those who did not use public transport because they did not need to or because of structural reasons was -0.02 m/s; although due to the smaller sample size (chair stands were only collected at waves 2,4 and 6), the 95% confidence intervals overlapped 0. Once chair stands and the interaction between physical activity and age were controlled for, these coefficients reduced by about half to -

For peer review only - http://bmjopen.bmj.com/site/about/guidelines.xhtml

0.01, suggesting that some of the association between public transport use and walking speed among older adults is statistically explained by lower limb muscle strength and physical activity.

We also used fixed effects models to examine whether changes in the use/non-use of public transport (for different reasons) were predicted by walking speed and the other covariates (Supplementary Table S5). For this analysis, we grouped the frequency variable (the dependent variable) into fewer groups: (1) used public transport, the reference category (2) did not use because of no need (3) did not use because of health problems and (4) did not use because of structural reasons. Slower walking speed and poorer health did not predict changes in the use/non-use of public transport because of the lack of need or structural reasons, but slower respondents were more likely not to use public transport for health reasons. Respondents with access to a car/van were less likely to use public transport. Respondents with mobility difficulties, low physical activity levels and higher levels of depressive symptoms were more likely to not use public transport for health reasons. Living in an isolated area increased the likelihood of a person reporting not using public transport because of structural reasons.

The results of the fixed effects model predicting grip strength are shown in online Supplementary Table S6. Unsurprisingly, older adults who did not use public transport due to health problems had weaker grip strength than those who used public transport nearly/every day. However, there were no differences in grip strength between the latter group and those who did not use public transport due to structural reasons or because they did not need to. In contrast, not using public transport because of structural barriers was associated with decrements in lower limb muscle strength. Such ELSA participants were more likely to become unable to complete the chair stand test, relative to those who completed the test quicker (Supplementary Table S7). Furthermore, older ELSA respondents who did not use public transport because they did not need to or because of structural barriers were also more likely to become sedentary relative to those engaging in high physical activity (Supplementary Table S8).

Turning to RQ3, in the 1st growth curve model (Table 3, column 3), we see similar estimates for the intercept and age coefficients compared to the fixed effects model. All the coefficients for the frequency variable were also negative, although the negative effect of not using public transport due to structural reasons on walking speed was small and not different from those who used public transport nearly every day (the reference group).

For peer review only - http://bmjopen.bmj.com/site/about/guidelines.xhtml

The second growth curve model added in the interaction between the frequency and age. With increasing age, the effect on walking speed of not using public transport for structural reasons or because the respondent did not feel the need to, became increasingly more negative. The trajectories of these three groups (those who used public transport nearly every day, those who did not use because of structural reasons, and those who did not use because they did not need to) are shown in Figures 1 and 2. In both figures, the decline in walking speed with age started to diverge around age 75, when there was a slower decline in walking speed for those who used public transport nearly every day, and a much steeper decline for those who did not use public transport because of structural reasons, or a lack of need. The upper 95% confidence intervals of the latter two groups clearly did not overlap with the estimated trajectories of the frequent public transport users after about the age of 75. We also estimated the growth curve model for those participants with at least three waves of walking speed measurements (Supplementary file Table S9) and found very similar estimates to the sample including all ELSA participants with at least one walking speed measurement (Table S3).

[Fig.1 and Fig.2 here]

Discussion

This study found evidence that older adults living in England who frequently used public transport had faster walking speeds than their peers who did not use public transport. Results from fixed effects and multilevel growth curve models showed similar patterns. In fixed effects models, frequent public transport use among older adults had a protective effect on walking speed. Unsurprisingly, not using public transport due to health reasons had the largest negative effect on walking speed. However, not using public transport due to other reasons also had a negative effect on walking speed. While the effect size of 0.02 m/s associated with not using public transport due to structural reasons may appear small, the predicted levels of walking speed in this cohort of older adults were well below the recommended 1.2 m/s walking speed needed for standard pedestrian crossings. [32] Any increase in the walking speed of older adults through factors such as physical activity and increased public transport use may help them cross the road safely.

The results of the fixed effects models were corroborated by the trajectories of walking speed decline shown in the growth curve models. In the growth curve models, older adults who did not use public transport due to structural reasons or because of a lack of need (“through choice”) had a faster decline in walking speed after the age of 75 than those who used public transport nearly every day.

For peer review only - http://bmjopen.bmj.com/site/about/guidelines.xhtml

The association between public transport use and muscle function deficits was specific to lower limb muscle strength, and did not extend to another aging related upper body muscle function deficit, grip strength. Frequent use of public transport appears to delay declines in muscles involved in walking, which in turn impacts on walking speed and related physical activity, not other aging related muscle function declines. The specificity of the association also suggests that potential confounders related to strength, fitness, and health were unlikely to cause the public transport use-walking speed association.

Existing studies have found that use of public transport contributes to better health by increasing physical activity [11 12 18] and reducing obesity. [18 19 23] However these studies have not examined the reasons why people do not use public transport. Limiting health is potentially a key factor that could confound any association between public transport use and subsequent health. The use of repeated measurements of public transport use (and the underlying reasons for non-use) and walking speed from a large, representative sample of older adults has been useful in taking account of this key confounding factor. Respondents who “use public transport nearly every day” may be positively health selected. In the analyses, we take account of changes in health conditions in a number of ways. First we control for different health conditions (depression, mobility problems, ADL) that vary across waves. Secondly, respondents could select limiting health as the main reason why they could not use public transport- this is the main negative health selection group. Moreover, we found little evidence that respondents with poorer health and slower walking speeds were more likely to report not using public transport because they did not need to or because of structural barriers.

Limitations

Longitudinal attrition across waves and other missing data may have resulted in a biased sample. The longitudinal sample tended to be older, healthier and more socio-economically advantaged. We used the wave specific cross sectional survey weights, which takes account of such predictors of non-response, in order to make the analyses representative of the older population but this may not adequately deal with attrition biases. Furthermore, there may be unobserved factors that cause the association between public transport use and walking speed.

Conclusion

For peer review only

It has become increasingly important for research to show a positive health impact from public transport use, especially among older adults, given cuts to public transport availability in England. [16] Savings to local government from cutting public transport may result in future increased expenditure on aging related conditions. Older adults who do not use public transport frequently are at risk of faster declines in their physical activity, lower limb muscle strength and walking speed compared to those who use public transport every day. This risk was evident not just among older adults who did not use public transport because of health problems, but also among those who did not use public transport because of structural barriers.

For peer review only

Acknowledgements

The data were made available through the UK Data Archive. ELSA was developed by a team of researchers based at the NatCen Social Research, University College London and the Institute for Fiscal Studies. The data were collected by NatCen Social Research. The funding is provided by the National Institute of Aging in the United States, and a consortium of UK government departments co-ordinated by the Office for National Statistics. The developers and funders of ELSA and the Archive do not bear any responsibility for the analyses or interpretations presented here.

PR participated in a Newton Fund/British Council/CONFAP Researcher Links Workshop, "Indicators for a Healthy City", in Belo Horizonte, Brazil, May 2016, which was coordinated by Professor Jennifer Mindell (UCL) and Professor Waleska Caiaffa (Federal University of Minas Gerais, UFMG).

Contributors: PR is responsible for study conception, study design, data analysis, and manuscript preparation and revision; had access to all of the data in the study; and takes responsibility for the integrity of the data and the accuracy of the data analysis. TC contributed to conception and design of the study, data analysis, data interpretation, manuscript preparation and revision. EW contributed to the design of the study, manuscript revision and provided input on the analysis of the study. All authors read and approved the final manuscript.

Funding: PR was supported by a grant from the Economic and Social Research Council [ES/P010075/1]. EW and TC were supported by a grant from the Economic and Social Research Council [ES/J019119/1]. TC was supported by a grant from the Medical Research Council [G1001375].

Disclaimer: The sponsors had no role in the study design; collection, analysis, and interpretation of the data; writing the report; or the decision to submit the report for publication.

Ethics approval: Ethical approval for all the ELSA waves was granted from NHS Research Ethics Committees under the National Research and Ethics Service (NRES). For further information see: http://www.nres.nhs.uk/.

Competing interest: None declared.

Data sharing statement: No additional data are available.

For peer review only

References

1. Cooper R, Kuh D, Hardy R. Objectively measured physical capability levels and mortality: systematic review and meta-analysis. *BMJ* 2010;341:c4467. doi: 10.1136/bmj.c4467 [published Online First: 2010/09/11]

2. Cooper R, Kuh D, Cooper C, et al. Objective measures of physical capability and subsequent health: a systematic review. *Age Ageing* 2011;40(1):14-23. doi: 10.1093/ageing/afq117 [published Online First: 2010/09/17]

3. Risser R, Lexell EM, Bell D, et al. Use of local public transport among people with cognitive impairments – A literature review. *Transportation Research Part F: Traffic Psychology and Behaviour* 2015;29:83-97. doi: 10.1016/j.trf.2015.01.002

4. Schwanen T, Lucas K, Akyelken N, et al. Rethinking the links between social exclusion and transport disadvantage through the lens of social capital. *Transportation Research Part A: Policy and Practice* 2015;74:123-35. doi: 10.1016/j.tra.2015.02.012

5. Green J, Jones A, Roberts H. More than A to B: the role of free bus travel for the mobility and wellbeing of older citizens in London. *Ageing and Society* 2012;34(3):472-94. doi: 10.1017/S0144686X12001110

6. Correa-de-Araujo R, Hadley E. Skeletal muscle function deficit: a new terminology to embrace the evolving concepts of sarcopenia and age-related muscle dysfunction. *J Gerontol A Biol Sci Med Sci* 2014;69(5):591-4. doi: 10.1093/gerona/glt208 [published Online First: 2014/04/17]

7. Montero-Fernandez N, Serra-Rexach JA. Role of exercise on sarcopenia in the elderly. *Eur J Phys Rehabil Med* 2013;49(1):131-43. [published Online First: 2013/04/12]

8. Freiberger E, Sieber C, Pfeifer K. Physical activity, exercise, and sarcopenia - future challenges. *Wien Med Wochenschr* 2011;161(17-18):416-25. doi: 10.1007/s10354-011-0001-z [published Online First: 2011/07/28]

9. Samitz G, Egger M, Zwahlen M. Domains of physical activity and all-cause mortality: systematic review and dose-response meta-analysis of cohort studies. *Int J Epidemiol* 2011;40(5):1382-400. doi: 10.1093/ije/dyr112

10. Barr A, Bentley R, Simpson JA, et al. Associations of public transport accessibility with walking, obesity, metabolic syndrome and diabetes. *Journal of Transport & Health* 2016;3(2):141-53. doi: 10.1016/j.jth.2016.01.006

11. Rissel C, Curac N, Greenaway M, et al. Physical Activity Associated with Public Transport Use—A Review and Modelling of Potential Benefits. *Int J Environ Res Public Health* 2012;9(7):2454-78. doi: 10.3390/ijerph9072454

12. Saelens BE, Vernez Moudon A, Kang B, et al. Relation Between Higher Physical Activity and Public Transit Use. *Am J Public Health* 2014;104(5):854-59. doi: 10.2105/AJPH.2013.301696

13. Mackett R. Improving accessibility for older people – Investing in a valuable asset. *Journal of Transport & Health* 2015;2(1):5-13. doi: 10.1016/j.jth.2014.10.004

14. Mackett R. Overcoming the Barriers to Access for Older People. Report Produced for the Age Action Alliance 2014. http://ageactionalliance.org/wordpress/wp-content/uploads/2014/11/Overcoming-the-barriers-to-access-Nov-14.pdf (accessed 10 Nov 2016).

15. Syed ST, Gerber BS, Sharp LK. Traveling towards disease: transportation barriers to health care access. *J Community Health* 2013;38(5):976-93. doi: 10.1007/s10900-013-9681-1

For peer review only

16. Department for Transport. Annual bus statistics: England 2015/16 2016. https://www.gov.uk/government/uploads/system/uploads/attachment_data/file/561060/annual-bus-statistics-year-ending-march-2016.pdf (accessed 10 Nov 2016).

17. Holley-Moore G, Creighton H. The Future of Transport in an Ageing Society London: ILC-UK; 2015. http://www.ilcuk.org.uk/index.php/publications/publication_details/the_future_of_transport_in_an_ageing_society (accessed 10 Nov 2016).

18. Webb E, Laverty A, Mindell J, et al. Free Bus Travel and Physical Activity, Gait Speed, and Adiposity in the English Longitudinal Study of Ageing. *Am J Public Health* 2016;106(1):136-42. doi: 10.2105/AJPH.2015.302907

19. Webb E, Netuveli G, Millett C. Free bus passes, use of public transport and obesity among older people in England. *J Epidemiol Community Health* 2012;66(2):176-80. doi: 10.1136/jech.2011.133165

20. Coronini-Cronberg S, Millett C, Laverty AA, et al. The impact of a free older persons' bus pass on active travel and regular walking in England. *Am J Public Health* 2012;102(11):2141-8. doi: 10.2105/ajph.2012.300946 [published Online First: 2012/09/22]

21. Weber D. Differences in physical aging measured by walking speed: evidence from the English Longitudinal Study of Ageing. *BMC Geriatr* 2016;16:31. doi: 10.1186/s12877-016-0201-x [published Online First: 2016/01/30]

22. Zaninotto P, Sacker A, Head J. Relationship between wealth and age trajectories of walking speed among older adults: evidence from the English Longitudinal Study of Ageing. *J Gerontol A Biol Sci Med Sci* 2013;68(12):1525-31. doi: 10.1093/gerona/glt058 [published Online First: 2013/05/18]

23. Flint E, Webb E, Cummins S. Change in commute mode and body-mass index: prospective, longitudinal evidence from UK Biobank. *The Lancet Public Health* 2016 doi: 10.1016/S2468-2667(16)30006-8

24. Broome K, McKenna K, Fleming J, et al. Bus use and older people: A literature review applying the Person–Environment–Occupation model in macro practice. *Scand J Occup Ther* 2009;16(1):3-12. doi: 10.1080/11038120802326222

25. Broome K, Nalder E, Worrall L, et al. Age-friendly buses? A comparison of reported barriers and facilitators to bus use for younger and older adults. *Australasian journal on ageing* 2010;29(1):33-8. doi: 10.1111/j.1741-6612.2009.00382.x [published Online First: 2010/04/20]

26. Cooper AJM, Lamb MJE, Sharp SJ, et al. Bidirectional association between physical activity and muscular strength in older adults: Results from the UK Biobank study. *Int J Epidemiol* 2017;46(1):141-48. doi: 10.1093/ije/dyw054

27. Steptoe A, Breeze E, Banks J, et al. Cohort Profile: The English Longitudinal Study of Ageing. *Int J Epidemiol* 2013,6(42):1640-09. doi:10.1093/ije/dys168.

28. Katz S, Ford AB, Moskowitz RW, et al. Studies of illness in the aged. The index of ADL: A standardized measure of biological and psychological function. *JAMA. 1963;185(12):914-919. doi:10.1001/jama.1963.03060120024016.*

29. Radloff LS. The CES-D Scale: A Self-Report Depression Scale for Research in the General Population. *Appl Psychol Meas* 1977;1(3):385-401. doi: 10.1177/014662167700100306

30. Demakakos P, Hamer M, Stamatakis E, et al. Low-intensity physical activity is associated with reduced risk of incident type 2 diabetes in older adults: evidence from the English Longitudinal Study of Ageing. *Diabetologia* 2010;53(9):1877-85. doi: 10.1007/s00125-010-1785-x [published Online First: 2010/05/25]

31. Bridges S, Hussey D, Blake M. The dynamics of ageing: The 2012 English Longitudinal Study of Ageing (Wave 6) Technical Report. 2015.

For peer review only - http://bmjopen.bmj.com/site/about/guidelines.xhtml

http://doc.ukdataservice.ac.uk/doc/5050/mrdoc/pdf/5050_elsa_w6_technical_report_v1.pdf (accessed 15 June 2017).

32. Bohannon RW, Williams Andrews A. Normal walking speed: a descriptive meta-analysis. *Physiotherapy* 2011;97(3):182-9. doi: 10.1016/j.physio.2010.12.004 [published Online First: 2011/08/09]

For peer review only

Figure Captions

Figure 1

Predicted decline in walking speed with age by public transport use, comparing ELSA respondents who use public transport every day and those who do not use public transport because of structural reasons

Figure 2

Predicted decline in walking speed with age by public transport use, comparing ELSA respondents who use public transport every day and those who do not use public transport because they do not need to

For peer review only

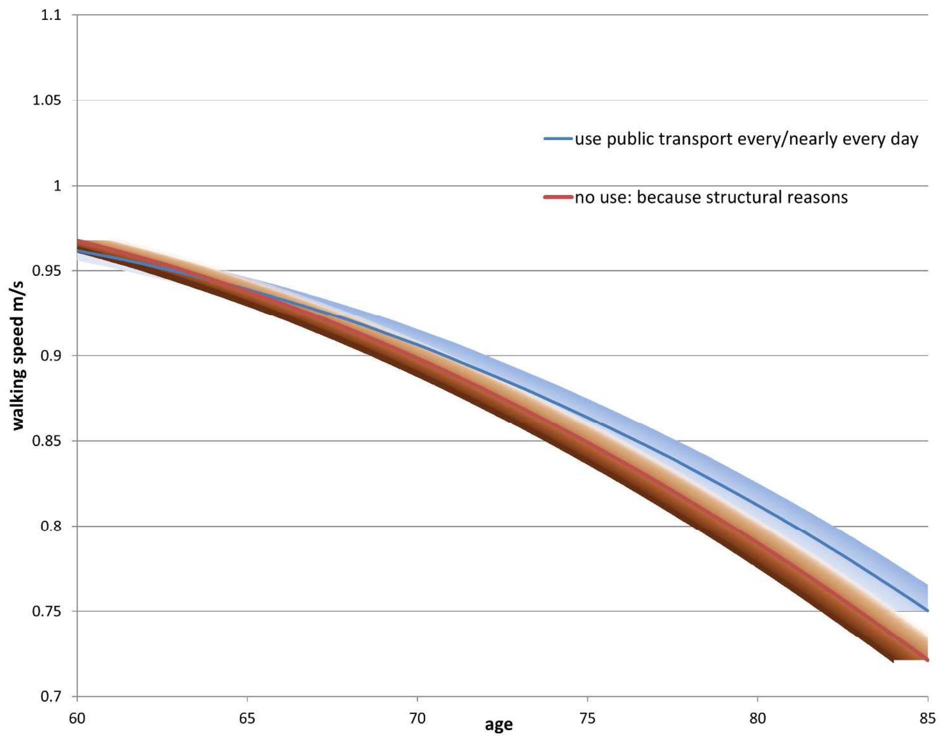

Predicted decline in walking speed with age by public transport use, comparing ELSA respondents who use public transport every day and those who do not use public transport because of structural reasons

254x190mm (300 x 300 DPI)

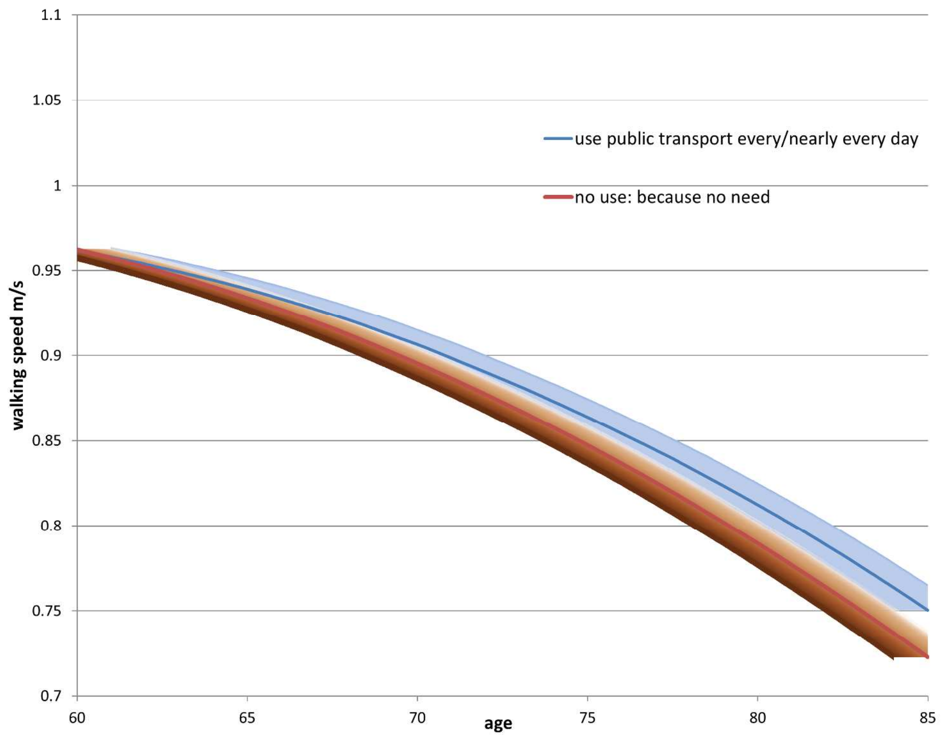

Predicted decline in walking speed with age by public transport use, comparing ELSA respondents who use public transport every day and those who do not use public transport because they do not need to

254x190mm (300 x 300 DPI)

For peer review only

Does public transport use prevent declines in walking speed among older adults living in England? A prospective cohort study

Patrick Rouxel, PhD[1], Elizabeth Webb, PhD [2] and Tarani Chandola PhD [3]

[1]CLOSER, Department of Social Science, University College London Institute of Education, London, UK, ORCID: 0000-0003-0330-554X, [2] International Centre for Lifecourse Studies, Department of Epidemiology and Public Health, University College London, London, UK, ORCID: 0000-0001-6106-5367, and [3]CMIST and Social Statistics, University of Manchester, UK, ORCID: 0000-0002-1864-3413

To BMJ Open

Corresponding author

Patrick Rouxel, PhD, CLOSER, Department of Social Science, University College London Institute of Education, 55-59 Gordon Square, WC1H 0NU, London, UK. E-mail: patrick.rouxel@ucl.ac.uk

For peer review only

Supplementary Table S1 Log odds (95% CI) from logit model of any missing data in the walking speed analyses, conditional on having a baseline walking speed measurement: ELSA waves 2-6

	Log odds	Lower 95% CI	Upper 95% CI
Intercept	**0.54**	-0.17	1.26
Gender (ref: Male)	**-0.22**	-0.31	-0.13
Age centered (linear term)	**-0.02**	-0.03	-0.01
*Gender*Age (ref: Male)*	**-0.01**	-0.02	-0.004
Age (quadratic term)	**0.01**	0.01	0.01
Frequency of public transport use(ref: every day or nearly every day)			
two or three times a week	-0.07	-0.24	0.09
once a week	**-0.32**	-0.48	-0.16
did not use because no need	0.02	-0.12	0.17
did not use because health problems	**0.69**	0.43	0.95
did not use because structural reasons	0.04	-0.11	0.19
Access to car/van (ref: yes access)			
No access to car/van	**0.25**	0.11	0.38
Employment status (ref: employed)			
Retired	**0.14**	0.02	0.25
Other	0.03	-0.10	0.15
Social class (ref: Managerial & Prof)			
Intermediate occupations	0.03	-0.09	0.16
Self-employed	**0.14**	0.01	0.27
Lower supervisory & technical	0.05	-0.09	0.18
Semi-routine & routine	**0.21**	0.11	0.31
Urban/Rural (ref: Urban)			
Town & Fringe	0.02	-0.10	0.13
Village	-0.13	-0.25	0.002
Hamlet/Isolated	0.08	-0.12	0.27
Mobility difficulties (ref: none)			
1-3 difficulties	-0.05	-0.14	0.04
4+ difficulties	**0.15**	0.01	0.29
Activities of Daily living impairments (ref: none)			
At least one ADL impairment	**0.16**	0.03	0.28
Physical Activity levels (ref: sedentary)			
Low	**-0.80**	-1.04	-0.55
Moderate	**-0.98**	-1.22	-0.74
High	**-1.00**	-1.25	-0.74
Marital status (ref: married)			
Separated/Divorced	**0.43**	0.25	0.61
Widowed	**0.45**	0.24	0.65
Never married	**0.66**	0.43	0.89
Cohabitation (ref: not living with partner)			
Living with partner	**0.69**	0.51	0.88
CESD- Depression score	**0.04**	0.01	0.06

For peer review only

Supplementary Table S1 continued Log odds (95% CI) from logit model of any missing data in the walking speed analyses, conditional on having a baseline walking speed measurement: ELSA waves 2-6

	**Log odds**	**Lower 95% CI**	**Upper 95% CI**
Smoking status (ref: never smoker)			
Ex-Smoker	-0.02	-0.10	0.06
Current smoker	**0.25**	0.13	0.36
Date/Day orientation (ref: no correct answer)			
1 correct answer	0.005	-0.93	0.94
2 correct answers	0.04	-0.67	0.75
3 correct answers	-0.47	-1.10	0.16
All correct answers	-0.52	-1.14	0.11
Memory test	**-0.06**	-0.07	-0.04

Boldface indicates statistical significance ($p<0.05$)

For peer review only

Supplementary Table S2 Coefficients (95% CI) from the Fixed Effects Model of walking speed (m/s), ELSA waves 2-6

	Coefficients	Lower 95% CI	Upper 95% CI
Intercept	**0.82**	0.76	0.88
Age centered (linear term)	**-0.01**	-0.01	-0.01
Age (quadratic term)	**-0.0003**	-0.0004	-0.0003
Frequency of public transport use(ref: every day or nearly every day)			
two or three times a week	-0.01	-0.02	0.0001
once a week	**-0.02**	-0.03	-0.01
did not use because no need	**-0.02**	-0.03	-0.003
did not use because health problems	**-0.06**	-0.08	-0.04
did not use because structural reasons	**-0.02**	-0.03	-0.01
Wealth quintiles (ref: poorest quintile)			
Quintile 2	0.01	-0.01	0.02
Quintile 3	0.01	-0.01	0.03
Quintile 4	0.02	-0.01	0.04
Richest quintile	**0.03**	0.003	0.05
Access to car/van (ref: yes access)			
No access to car/van	-0.01	-0.02	0.0005
Employment status (ref: employed)			
Retired	0.00	-0.01	0.01
Other	0.00	-0.01	0.02
Social class (ref: Managerial & Prof)			
Intermediate occupations	-0.02	-0.06	0.02
Self-employed	0.01	-0.03	0.05
Lower supervisory & technical	-0.02	-0.06	0.03
Semi-routine & routine	0.02	-0.02	0.06
Urban/Rural (ref: Urban)			
Town & Fringe	0.02	-0.03	0.06
Village	0.01	-0.03	0.05
Hamlet/Isolated	0.03	-0.03	0.08
Mobility difficulties (ref: none)			
1-3 difficulties	**-0.01**	-0.02	-0.01
4+ difficulties	**-0.05**	-0.06	-0.04
Activities of Daily living impairments (ref: none)			
At least one ADL impairment	**-0.03**	-0.04	-0.02
Physical Activity levels (ref: sedentary)			
Low	**0.03**	0.02	0.04
Moderate	**0.05**	0.04	0.07
High	**0.06**	0.05	0.08
Marital status (ref: married)			
Separated/Divorced	**0.04**	0.01	0.07
Widowed	0.01	-0.02	0.04
Never married	0.02	-0.04	0.07
Cohabitation (ref: not living with partner)			
Living with partner	0.01	-0.02	0.05
CESD- Depression score	-0.01	-0.01	-0.01

For peer review only

Supplementary Table S2 continued Coefficients (95% CI) from the Fixed Effects Model of walking speed (m/s), ELSA waves 2-6

Smoking status (ref: never smoker)	**Coefficients**	**Lower 95% CI**	**Upper 95% CI**
Ex-Smoker	0.00	-0.02	0.03
Current smoker	0.01	-0.02	0.04
Date/Day orientation (ref: no correct answer)			
1 correct answer	0.02	-0.03	0.07
2 correct answers	0.04	-0.01	0.08
3 correct answers	**0.05**	0.01	0.09
All correct answers	**0.05**	0.01	0.09
Memory test	**0.001**	0.00002	0.002
Number of observations	27509		
Adjusted R-squared	0.73		

Boldface indicates statistical significance (p<0.05)

For peer review only

Supplementary Table S3 Coefficients (95% CI) from the Multilevel Growth Curve Model of walking speed (m/s), ELSA waves 2-6

**Fixed Part**	**Coefficients**	**Lower 95% CI**	**Upper 95% CI**
Intercept	**0.83**	0.79	0.87
Age centered (linear term)	**-0.01**	-0.01	-0.004
Age (quadratic term)	**-0.0002**	-0.0002	-0.0002
Frequency of public transport use(ref: every day or nearly every day)			
two or three times a week	-0.01	-0.02	0.01
once a week	-0.01	-0.02	0.01
did not use because no need	-0.005	-0.02	0.01
did not use because health problems	**-0.12**	-0.14	-0.09
did not use because structural reasons	-0.002	-0.014	0.011
Interaction between Age (linear term) and Frequency of public transport use (ref: nearly/every day use)			
Age*two or three times a week	-0.0003	-0.0017	0.0011
Age*once a week	-0.0010	-0.0024	0.0004
Age*did not use because no need	**-0.0012**	-0.0024	-0.00002
Age*did not use because health problems	**0.0017**	0.0001	0.0033
Age*did not use because structural reasons	**-0.0014**	-0.0026	-0.0002
Sex (ref: men)			
Women	**-0.02**	-0.03	-0.02
Wealth quintiles (ref: poorest quintile)			
Quintile 2	**0.02**	0.01	0.03
Quintile 3	**0.04**	0.03	0.05
Quintile 4	**0.06**	0.05	0.07
Richest quintile	**0.09**	0.08	0.10
Access to car/van (ref: yes access)			
No access to car/van	**-0.02**	-0.03	-0.02
Employment status (ref: employed)			
Retired	-0.01	-0.02	0.00002
Other	**-0.02**	-0.03	-0.01
Social class (ref: Managerial & Prof)			
Intermediate occupations	**-0.02**	-0.03	-0.01
Self-employed	**-0.02**	-0.03	-0.01
Lower supervisory & technical	**-0.05**	-0.06	-0.04
Semi-routine & routine	**-0.04**	-0.05	-0.03
Urban/Rural (ref: Urban)			
Town & Fringe	**0.01**	0.003	0.02
Village	**0.02**	0.01	0.03
Hamlet/Isolated	**0.02**	0.00	0.04
Mobility difficulties (ref: none)			
1-3 difficulties	**-0.04**	-0.04	-0.03
4+ difficulties	**-0.12**	-0.13	-0.11
Activities of Daily living impairments (ref: none)			
At least one ADL impairment	**-0.06**	-0.06	-0.05

For peer review only

Supplementary Table S3 continued Coefficients (95% CI) from the Multilevel Growth Curve Model of walking speed (m/s), ELSA waves 2-6

Physical Activity levels (ref: sedentary)	**Coefficients**	**Lower 95% CI**	**Upper 95% CI**
Low	**0.05**	0.04	0.06
Moderate	**0.10**	0.08	0.11
High	**0.12**	0.11	0.13
Marital status (ref: married)			
Separated/Divorced	0.001	-0.01	0.01
Widowed	-0.003	-0.01	0.01
Never married	**-0.03**	-0.05	-0.01
Cohabitation (ref: not living with partner)			
Living with partner	0.01	-0.01	0.03
CESD- Depression score	**-0.01**	-0.01	-0.01
Smoking status (ref: never smoker)			
Ex-Smoker	-0.01	-0.01	0.001
Current smoker	**-0.03**	-0.04	-0.02
Date/Day orientation (ref: no correct answer)			
1 correct answer	0.01	-0.03	0.05
2 correct answers	**0.03**	-0.01	0.07
3 correct answers	**0.05**	0.02	0.08
All correct answers	**0.05**	0.02	0.09
Memory test	**0.01**	0.004	0.01
**Random Part**			
Level 2 (Individual)			
Intercept variance	0.02		
Age centered (linear term) variance	0.00002		
Covariance of intercept and age centered	-0.0004		
Level 1 (wave)			
Intercept	0.02		
Number of observations (level 1)	9656		
Number of clusters (level 2)	27509		
-2*Log Likelihood	-13746.54		

Boldface indicates statistical significance (p<0.05)

For peer review only

Supplementary Table S4 Selected coefficients (95% CI) from two Fixed Effects Models of walking speed (m/s), ELSA waves 2-6

**Model 1: excluding chair stand and physical activity**	**Coefficients**	**Lower 95% CI**	**Upper 95% CI**
Frequency of public transport use(ref: every day or nearly every day)			
two or three times a week	-0.02	-0.04	0.01
once a week	**-0.03**	-0.05	-0.001
did not use because no need	-0.02	-0.04	0.01
did not use because health problems	**-0.08**	-0.11	-0.05
did not use because structural reasons	-0.02	-0.04	0.01

Model 1 includes age, age squared, frequency of public transport use, gender, wealth, car/van access, employment status, social class, urban/rural, mobility difficulties, disability, marital status, cohabitation, CES-D depression score, smoking status memory test, time/date orientation

**Model 2: including chair stand and physical activity**	**Coefficients**	**Lower 95% CI**	**Upper 95% CI**
Frequency of public transport use(ref: every day or nearly every day)			
two or three times a week	-0.01	-0.03	0.01
once a week	-0.02	-0.05	0.003
did not use because no need	-0.01	-0.04	0.01
did not use because health problems	**-0.06**	-0.09	-0.03
did not use because structural reasons	-0.01	-0.04	0.01

Model 2 includes all the variables in Model 1 as well as chair stand, physical activity and the interaction between physical activity and age. Model 1 is nested within Model 2.

Boldface indicates statistical significance (p<0.05)

For peer review only

Supplementary Table S5 Coefficients (95% CI) from the Fixed Effects multinomial model of public transport use, ELSA waves 2-6. Coefficients are log odds of not using public transport for different reasons relative to using public transport at least once a week

	**no use: no need**			**no use: health problems**			**no use: structural reasons**		
	Log odds	Lower 95% CI	Upper 95% CI	Log odds	Lower 95% CI	Upper 95% CI	Log odds	Lower 95% CI	Upper 95% CI
Walking speed	-0.16	-0.55	0.22	**-2.39**	-3.29	-1.48	-0.26	-0.64	0.12
Age centered (linear term)	**0.05**	0.02	0.08	**0.12**	0.04	0.19	-0.003	-0.03	0.03
Age (quadratic term)	**0.004**	0.002	0.01	**0.01**	0.01	0.01	**0.005**	0.003	0.01
Wealth quintiles (ref: poorest quintile)									
Quintile 2	-0.33	-0.76	0.10	**-0.96**	-1.80	-0.11	-0.20	-0.69	0.29
Quintile 3	-0.24	-0.70	0.22	-0.73	-1.64	0.17	-0.18	-0.69	0.34
Quintile 4	-0.15	-0.64	0.34	-0.82	-1.81	0.16	-0.02	-0.56	0.52
Richest quintile	-0.38	-0.91	0.14	-1.12	-2.28	0.04	-0.30	-0.87	0.28
Access to car/van (ref: yes access)									
No access to car/van	**-1.20**	-1.49	-0.90	**-0.57**	-0.97	-0.16	**-1.02**	-1.35	-0.68
Employment status (ref: employed)									
Retired	-0.18	-0.44	0.09	0.42	-0.61	1.45	**-0.36**	-0.62	-0.10
Other	-0.16	-0.49	0.17	0.51	-0.57	1.59	**-0.45**	-0.78	-0.12
Social class (ref: Managerial & Prof)									
Intermediate occupations	-0.11	-0.97	0.74	-14.63	-1940.9	1911.7	-0.23	-1.05	0.58
Self-employed	-0.35	-1.19	0.48	-2.87	-6.39	0.65	**-0.89**	-1.72	-0.06
Lower supervisory & technical	0.89	-0.16	1.94	-0.50	-5.30	4.29	0.51	-0.49	1.50
Semi-routine & routine	-0.36	-1.15	0.44	-2.35	-4.81	0.11	-0.72	-1.47	0.04
Urban/Rural (ref: Urban)									
Town & Fringe	**0.75**	0.01	1.49	-1.20	-2.78	0.38	0.60	-0.06	1.25
Village	0.38	-0.62	1.37	1.23	-0.39	2.84	0.59	-0.24	1.43
Hamlet/Isolated	0.29	-1.19	1.77	-0.90	-4.16	2.35	**2.01**	0.66	3.37

For peer review only - http://bmjopen.bmj.com/site/about/guidelines.xhtml

Supplementary Table S5 continued Coefficients (95% CI) from the Fixed Effects multinomial model of public transport use, ELSA waves 2-6. Coefficients are log odds of not using public transport for different reasons relative to using public transport at least once a week

	**no use: no need**			**no use: health problems**			**no use: structural reasons**		
Mobility difficulties (ref:none)	Log odds	Lower 95% CI	Upper 95% CI	Log odds	Lower 95% CI	Upper 95% CI	Log odds	Lower 95% CI	Upper 95% CI
1-3 difficulties	-0.10	-0.26	0.06	**0.60**	0.07	1.14	0.03	-0.13	0.20
4+ difficulties	-0.06	-0.33	0.21	**1.43**	0.83	2.03	0.26	-0.01	0.54
Activities of Daily living impairments (ref: none)									
At least one ADL impairment	0.10	-0.11	0.31	0.16	-0.15	0.46	0.06	-0.15	0.27
Physical Activity levels (ref: sedentary)									
Low	0.01	-0.32	0.35	-0.26	-0.67	0.14	-0.24	-0.58	0.11
Moderate	0.09	-0.24	0.43	**-0.57**	-1.04	-0.09	-0.26	-0.62	0.09
High	0.10	-0.27	0.48	**-0.98**	-1.77	-0.19	-0.27	-0.65	0.12
Marital status (ref: married)									
Separated/Divorced	0.17	-0.56	0.90	-0.98	-2.41	0.45	0.44	-0.30	1.18
Widowed	-0.01	-0.67	0.65	-0.19	-1.39	1.01	-0.28	-0.96	0.39
Never married	0.57	-0.78	1.92	-0.68	-3.87	2.52	0.29	-1.13	1.71
Cohabitation (ref: not living with partner)									
Living with partner	0.61	-0.02	1.24	0.22	-1.00	1.44	0.34	-0.30	0.98
CESD- Depression score	-0.04	-0.08	0.01	**0.10**	0.02	0.18	0.002	-0.04	0.05
Smoking status (ref: never smoker)									
Ex-Smoker	-0.24	-0.77	0.29	**-1.18**	-2.21	-0.14	-0.27	-0.82	0.27
Current smoker	0.12	-0.55	0.79	-0.67	-1.94	0.59	-0.04	-0.73	0.65
Date/Day orientation (ref: no correct answer)									
1 correct answer	1.02	-0.23	2.27	1.06	-0.45	2.57	0.69	-0.60	1.99
2 correct answers	0.70	-0.40	1.79	0.38	-1.01	1.77	0.53	-0.61	1.67
3 correct answers	0.49	-0.53	1.52	0.52	-0.82	1.86	0.13	-0.94	1.19
All correct answers	0.56	-0.46	1.58	0.55	-0.79	1.88	0.11	-0.95	1.17
Memory test	0.00	-0.03	0.02	0.00	-0.05	0.05	-0.01	-0.04	0.01

Boldface indicates statistical significance (p<0.05)

For peer review only - http://bmjopen.bmj.com/site/about/guidelines.xhtml

Supplementary Table S6 Coefficients (95% CI) from the Fixed Effects Model of grip strength (kg), ELSA waves 2-6

	Coefficients	Lower 95% CI	Upper 95% CI
Intercept	**28.47**	26.21	30.72
Age centered (linear term)	**-0.44**	-0.47	-0.41
Age (quadratic term)	**-0.005**	-0.007	-0.003
Frequency of public transport use(ref: every day or nearly every day)			
two or three times a week	-0.37	-0.88	0.14
once a week	-0.12	-0.68	0.44
did not use because no need	-0.03	-0.63	0.56
did not use because health problems	**-1.35**	-2.09	-0.61
did not use because structural reasons	-0.13	-0.70	0.45
Wealth quintiles (ref: poorest quintile)			
Quintile 2	-0.42	-1.17	0.33
Quintile 3	-0.35	-1.15	0.46
Quintile 4	-0.32	-1.17	0.54
Richest quintile	-0.06	-1.00	0.88
Access to car/van (ref: yes access)			
No access to car/van	0.08	-0.40	0.56
Employment status (ref: employed)			
Retired	-0.22	-0.67	0.22
Other	-0.96	-1.59	-0.33
Social class (ref: Managerial & Prof)			
Intermediate occupations	0.28	-1.43	1.99
Self-employed	-0.39	-1.44	0.65
Lower supervisory & technical	-0.76	-1.98	0.47
Semi-routine & routine	0.22	-0.86	1.31
Urban/Rural (ref: Urban)			
Town & Fringe	0.31	-0.65	1.27
Village	0.08	-1.16	1.32
Hamlet/Isolated	0.05	-1.35	1.45
Mobility difficulties (ref: none)			
1-3 difficulties	-0.19	-0.47	0.08
4+ difficulties	-1.03	-1.50	-0.57
Activities of Daily living impairments (ref: none)			
At least one ADL impairment	**-0.69**	-1.07	-0.30
Physical Activity levels (ref: sedentary)			
Low	**0.69**	0.07	1.30
Moderate	**0.73**	0.10	1.37
High	**0.95**	0.24	1.67
Marital status (ref: married)			
Separated/Divorced	-0.81	-1.90	0.28
Widowed	-0.23	-1.32	0.87
Never married	-1.47	-3.75	0.82
Cohabitation (ref: not living with partner)			
Living with partner	-0.47	-1.56	0.63
CESD- Depression score	-0.05	-0.12	0.03

For peer review only - http://bmjopen.bmj.com/site/about/guidelines.xhtml

Supplementary Table S6 continued Coefficients (95% CI) from the Fixed Effects Model of grip strength (kg), ELSA waves 2-6

Smoking status (ref: never smoker)	Coefficients	Lower 95% CI	Upper 95% CI
Ex-Smoker	0.46	-0.35	1.27
Current smoker	0.76	-0.36	1.87
Date/Day orientation (ref: no correct answer)			
1 correct answer	0.70	-1.31	2.71
2 correct answers	**1.94**	0.17	3.71
3 correct answers	**1.93**	0.35	3.51
All correct answers	**1.87**	0.30	3.44
Memory test	0.02	-0.02	0.07
Number of observations	21835		
Adjusted R-squared	0.87		

Boldface indicates statistical significance (p<0.05)

For peer review only

Supplementary Table S7 Coefficients (95% CI) from the Fixed Effects multinomial model of chair stand outcomes, ELSA waves 2-6. Coefficients are log odds of not completing the test or taking longer than the median time to complete the test relative to using completing the chair stand test faster than the median time

	**Did not complete chair stand test**			**Took longer to complete 5/10 chair rises**		
Intercept	**Log odds**	**Lower 95% CI**	**Upper 95% CI**	**Log odds**	**Lower 95% CI**	**Upper 95% CI**
Age centered (linear term)	**0.09**	0.06	0.11	**0.10**	0.09	0.12
Age (quadratic term)	**0.004**	0.003	0.006	**0.002**	0.001	0.003
Frequency of public transport use(ref: every day or nearly every day)						
two or three times a week	0.00	-0.38	0.39	-0.01	-0.31	0.30
once a week	0.10	-0.34	0.53	-0.12	-0.46	0.21
did not use because no need	0.31	-0.12	0.75	0.15	-0.18	0.47
did not use because health problems	**1.28**	0.63	1.92	0.43	-0.16	1.02
did not use because structural reasons	**0.45**	0.02	0.89	0.05	-0.27	0.38
Wealth quintiles (ref: poorest quintile)						
Quintile 2	-0.39	-0.92	0.15	-0.29	-0.73	0.15
Quintile 3	-0.12	-0.70	0.47	-0.42	-0.89	0.04
Quintile 4	-0.32	-0.95	0.31	-0.46	-0.94	0.03
Richest quintile	-0.24	-0.94	0.46	-0.42	-0.93	0.10
Access to car/van (ref: yes access)						
No access to car/van	-0.20	-0.58	0.19	-0.28	-0.62	0.05
Employment status (ref: employed)						
Retired	**0.50**	0.16	0.84	0.05	-0.16	0.26
Other	**0.41**	0.01	0.81	0.09	-0.18	0.37
Social class (ref: Managerial & Prof)						
Intermediate occupations	0.78	-0.13	1.70	-0.08	-0.67	0.50
Self-employed	0.44	-0.42	1.29	0.19	-0.37	0.74
Lower supervisory & technical	-0.14	-1.19	0.90	-0.25	-0.97	0.46
Semi-routine & routine	0.16	-0.66	0.99	0.48	-0.05	1.01

For peer review only - http://bmjopen.bmj.com/site/about/guidelines.xhtml

Supplementary Table S7 continued Coefficients (95% CI) from the Fixed Effects multinomial model of chair stand outcomes, ELSA waves 2-6. Coefficients are log odds of not completing the test or taking longer than the median time to complete the test relative to using completing the chair stand test faster than the median time

	**Did not complete chair stand test**			**Took longer to complete 5/10 chair rises**		
Urban/Rural (ref: Urban)	**Log odds**	**Lower 95% CI**	**Upper 95% CI**	**Log odds**	**Lower 95% CI**	**Upper 95% CI**
Town & Fringe	0.09	-0.85	1.03	0.26	-0.40	0.92
Village	-0.69	-1.63	0.26	0.19	-0.55	0.93
Hamlet/Isolated	**1.54**	0.03	3.05	0.47	-0.49	1.43
Mobility difficulties (ref: none)						
1-3 difficulties	0.10	-0.15	0.34	0.11	-0.05	0.26
4+ difficulties	**0.83**	0.46	1.19	**0.37**	0.10	0.63
Activities of Daily living impairments (ref: none)						
At least one ADL impairment	**0.46**	0.18	0.73	**0.06**	-0.17	0.29
Physical Activity levels (ref: sedentary)						
Low	-0.14	-0.63	0.35	0.20	-0.25	0.66
Moderate	-0.48	-0.98	0.01	-0.09	-0.55	0.36
High	-0.21	-0.76	0.33	-0.11	-0.58	0.37
Marital status (ref: married)						
Separated/Divorced	-0.38	-1.22	0.46	-0.38	-1.00	0.25
Widowed	-0.55	-1.37	0.28	-0.37	-0.97	0.23
Never married	-0.31	-1.93	1.30	-1.22	-2.45	0.01
Cohabitation (ref: not living with partner)						
Living with partner	-0.66	-1.45	0.13	-0.29	-0.85	0.27
CESD- Depression score	**0.07**	0.02	0.13	0.01	-0.04	0.05
Smoking status (ref: never smoker)						
Ex-Smoker	-0.30	-0.99	0.39	0.15	-0.35	0.66
Current smoker	-0.58	-1.43	0.27	-0.01	-0.63	0.61

For peer review only - http://bmjopen.bmj.com/site/about/guidelines.xhtml

For peer review only

Supplementary Table S7 continued Coefficients (95% CI) from the Fixed Effects multinomial model of chair stand outcomes, ELSA waves 2-6. Coefficients are log odds of not completing the test or taking longer than the median time to complete the test relative to using completing the chair stand test faster than the median time

	Did not complete chair stand test			Took longer to complete 5/10 chair rises		
Date/Day orientation (ref: no correct answer)	**Log odds**	**Lower 95% CI**	**Upper 95% CI**	**Log odds**	**Lower 95% CI**	**Upper 95% CI**
1 correct answer	0.01	-2.15	2.17	-0.02	-1.72	1.67
2 correct answers	-0.89	-2.59	0.80	-0.01	-1.43	1.40
3 correct answers	-0.58	-2.19	1.03	0.34	-1.01	1.69
All correct answers	-0.68	-2.28	0.92	0.21	-1.14	1.55
Memory test	-0.03	-0.06	0.01	0.00	-0.03	0.02
Number of observations	12463					
Adjusted R-squared	0.10					

Boldface indicates statistical significance (p<0.05)

For peer review only - http://bmjopen.bmj.com/site/about/guidelines.xhtml

Supplementary Table S8 Coefficients (95% CI) from the Fixed Effects multinomial model of physical activity, ELSA waves 2-6. Coefficients are log odds of sedentary/low/moderate physical activity, relative to being in the high physical activity category

	Sedentary physical activity			Low physical activity			Moderate physical activity		
	Log odds	Lower 95% CI	Upper 95% CI	Log odds	Lower 95% CI	Upper 95% CI	Log odds	Lower 95% CI	Upper 95% CI
Age centered (linear term)	**0.17**	0.11	0.22	**0.10**	0.06	0.13	0.02	-0.01	0.05
Age (quadratic term)	**0.006**	0.004	0.007	**0.005**	0.004	0.006	**0.002**	0.001	0.003
Frequency of public transport use(ref: every day or nearly every day)									
two or three times a week	-0.18	-0.64	0.28	**-0.33**	-0.62	-0.03	-0.15	-0.40	0.10
once a week	0.06	-0.47	0.60	-0.24	-0.57	0.08	-0.10	-0.37	0.17
did not use because no need	-0.22	-0.75	0.30	-0.16	-0.49	0.16	-0.08	-0.35	0.19
did not use because health problems	1.37	0.65	2.09	0.81	0.23	1.39	0.39	-0.17	0.94
did not use because structural reasons	0.10	-0.43	0.63	-0.10	-0.42	0.22	-0.08	-0.35	0.19
Interaction between Age (linear term) and Frequency of public transport use (ref: Nearly/Every day use)									
Age*two or three times a week	0.04	-0.01	0.08	0.03	-0.001	0.07	0.03	-0.001	0.06
Age*once a week	0.04	-0.01	0.09	0.02	-0.02	0.06	0.02	-0.01	0.05
Age*did not use because no need	**0.06**	0.01	0.11	0.02	-0.01	0.06	**0.04**	0.01	0.07
Age*did not use because health problems	0.06	-0.003	0.13	0.02	-0.03	0.08	0.02	-0.04	0.07
Age*did not use because structural reasons	**0.06**	0.01	0.11	0.03	-0.01	0.06	**0.04**	0.01	0.07
Wealth quintiles (ref: poorest quintile)									
Quintile 2	0.21	-0.36	0.78	0.34	-0.03	0.72	**0.35**	0.04	0.66
Quintile 3	-0.02	-0.64	0.60	0.21	-0.20	0.61	0.30	-0.04	0.63
Quintile 4	-0.28	-0.95	0.39	0.02	-0.40	0.45	0.26	-0.09	0.61
Richest quintile	-0.66	-1.45	0.13	0.03	-0.43	0.49	0.29	-0.08	0.67
Access to car/van (ref: yes access)									
No access to car/van	**0.40**	0.05	0.76	0.08	-0.21	0.38	-0.01	-0.28	0.25
Employment status (ref: employed)									
Retired	**0.79**	0.24	1.33	0.06	-0.15	0.28	0.01	-0.15	0.16
Other	**0.73**	0.16	1.30	0.08	-0.18	0.34	0.02	-0.18	0.23

For peer review only - http://bmjopen.bmj.com/site/about/guidelines.xhtml

Supplementary Table S8 continued Coefficients (95% CI) from the Fixed Effects multinomial model of physical activity, ELSA waves 2-6. Coefficients are log odds of sedentary/low/moderate physical activity, relative to being in the high physical activity category

	**Sedentary physical activity**			**Low physical activity**			**Moderate physical activity**		
Social class (ref: Managerial & Prof)	**Log odds**	**Lower 95% CI**	**Upper 95% CI**	**Log odds**	**Lower 95% CI**	**Upper 95% CI**	**Log odds**	**Lower 95% CI**	**Upper 95% CI**
Intermediate occupations	-0.97	-2.46	0.52	-0.53	-1.09	0.02	-0.21	-0.60	0.17
Self-employed	0.09	-1.37	1.55	**-0.79**	-1.36	-0.22	**-0.47**	-0.85	-0.09
Lower supervisory & technical	**-1.85**	-3.52	-0.18	-0.65	-1.36	0.07	-0.06	-0.51	0.40
Semi-routine & routine	-1.23	-2.67	0.21	-0.35	-0.87	0.18	-0.19	-0.54	0.16
Urban/Rural (ref: Urban)									
Town & Fringe	-0.62	-1.81	0.57	0.41	-0.21	1.04	0.28	-0.20	0.76
Village	0.23	-1.22	1.69	0.12	-0.53	0.76	-0.42	-0.91	0.08
Hamlet/Isolated	-0.01	-1.77	1.76	-0.22	-1.16	0.73	-0.35	-1.09	0.39
Mobility difficulties (ref: none)									
1-3 difficulties	-0.05	-0.36	0.26	**0.25**	0.10	0.39	**0.15**	0.03	0.26
4+ difficulties	**0.86**	0.46	1.26	**0.97**	0.71	1.23	**0.52**	0.29	0.75
Activities of Daily living impairments (ref: none)									
At least one ADL impairment	**0.63**	0.36	0.90	**0.27**	0.07	0.48	0.07	-0.11	0.26
Marital status (ref: married)									
Separated/Divorced	0.48	-0.35	1.31	0.25	-0.29	0.79	0.23	-0.20	0.65
Widowed	0.12	-0.74	0.98	-0.02	-0.58	0.55	0.12	-0.34	0.59
Never married	1.07	-0.50	2.63	-0.06	-1.01	0.89	-0.05	-0.78	0.68
Cohabitation (ref: not living with partner)									
Living with partner	0.62	-0.23	1.47	0.45	-0.08	0.98	0.23	-0.19	0.65
CESD- Depression score	**0.19**	0.14	0.25	**0.14**	0.10	0.18	**0.06**	0.03	0.09
Smoking status (ref: never smoker)									
Ex-Smoker	-0.47	-1.31	0.36	-0.33	-0.81	0.15	-0.28	-0.67	0.11
Current smoker	-0.63	-1.60	0.34	-0.57	-1.16	0.01	-0.28	-0.76	0.20

For peer review only

Supplementary Table S8 continued Coefficients (95% CI) from the Fixed Effects multinomial model of physical activity, ELSA waves 2-6. Coefficients are log odds of sedentary/low/moderate physical activity, relative to being in the high physical activity category

	**Sedentary physical activity**			**Low physical activity**			**Moderate physical activity**		
Date/Day orientation (ref: no correct answer)	**Log odds**	**Lower 95% CI**	**Upper 95% CI**	**Log odds**	**Lower 95% CI**	**Upper 95% CI**	**Log odds**	**Lower 95% CI**	**Upper 95% CI**
1 correct answer	0.26	-1.42	1.94	-0.36	-1.91	1.18	-0.63	-2.12	0.85
2 correct answers	-0.70	-2.20	0.79	-0.63	-2.02	0.76	-0.70	-2.03	0.63
3 correct answers	-0.91	-2.34	0.52	-0.55	-1.88	0.78	-0.57	-1.84	0.71
All correct answers	-1.05	-2.48	0.37	-0.62	-1.95	0.70	-0.62	-1.89	0.65
Memory test	**-0.05**	-0.09	-0.02	**-0.03**	-0.05	-0.004	-0.02	-0.03	0.003
Number of observations	32,276								
Adjusted R-squared	0.06								

Boldface indicates statistical significance (p<0.05)

For peer review only

For peer review only - http://bmjopen.bmj.com/site/about/guidelines.xhtml

Supplementary Table S9 Coefficients (95% CI) from the Multilevel Growth Curve Model of walking speed (m/s), ELSA waves 2-6: Only participants with 3 or more waves of data

**Fixed Part**	**Coefficients**	**Lower 95% CI**	**Upper 95% CI**
Intercept	**0.80**	0.75	0.86
Age centered (linear term)	**-0.01**	-0.01	-0.01
Age (quadratic term)	**-0.0002**	-0.0003	-0.0002
Frequency of public transport use(ref: every day or nearly every day)			
two or three times a week	-0.01	-0.02	0.0003
once a week	**-0.01**	-0.03	-0.001
did not use because no need	**-0.02**	-0.03	-0.003
did not use because health problems	**-0.09**	-0.11	-0.08
did not use because structural reasons	**-0.011**	-0.023	0.001
Interaction between Age (linear term) and Frequency of public transport use (ref: nearly/every day use)			
Age*two or three times a week	-0.0003	-0.0018	0.0013
Age*once a week	-0.0016	-0.0032	0.00003
Age*did not use because no need	-0.0012	-0.0027	0.0004
Age*did not use because health problems	0.0005	-0.0014	0.0024
Age*did not use because structural reasons	**-0.0019**	-0.0034	-0.0004
Sex (ref: men)			
Women	-0.03	-0.03	-0.02
Wealth quintiles (ref: poorest quintile)			
Quintile 2	**0.02**	0.01	0.03
Quintile 3	**0.03**	0.02	0.04
Quintile 4	**0.05**	0.04	0.07
Richest quintile	**0.08**	0.06	0.09
Access to car/van (ref: yes access)			
No access to car/van	**-0.02**	-0.03	-0.01
Employment status (ref: employed)			
Retired	-0.01	-0.02	0.002
Other	**-0.02**	-0.03	-0.003
Social class (ref: Managerial & Prof)			
Intermediate occupations	**-0.02**	-0.04	-0.01
Self-employed	**-0.02**	-0.04	-0.01
Lower supervisory & technical	**-0.04**	-0.05	-0.02
Semi-routine & routine	**-0.05**	-0.06	-0.04
Urban/Rural (ref: Urban)			
Town & Fringe	0.01	-0.004	0.02
Village	**0.02**	0.01	0.03
Hamlet/Isolated	**0.02**	0.00	0.04
Mobility difficulties (ref: none)			
1-3 difficulties	**-0.03**	-0.04	-0.03
4+ difficulties	**-0.11**	-0.12	-0.10
Activities of Daily living impairments (ref: none)			
At least one ADL impairment	**-0.05**	-0.06	-0.04

For peer review only - http://bmjopen.bmj.com/site/about/guidelines.xhtml

Supplementary Table S9 continued Coefficients (95% CI) from the Multilevel Growth Curve Model of walking speed (m/s), ELSA waves 2-6: Only participants with 3 or more waves of data

Physical Activity levels (ref: sedentary)	**Coefficients**	**Lower 95% CI**	**Upper 95% CI**
Low	**0.04**	0.03	0.06
Moderate	**0.09**	0.07	0.10
High	**0.11**	0.09	0.12
Marital status (ref: married)			
Separated/Divorced	**0.03**	0.003	0.05
Widowed	0.02	-0.01	0.04
Never married	-0.004	-0.03	0.03
Cohabitation (ref: not living with partner)			
Living with partner	0.02	-0.002	0.04
CESD- Depression score	**-0.01**	-0.01	-0.01
Smoking status (ref: never smoker)			
Ex-Smoker	**-0.01**	-0.02	-0.002
Current smoker	**-0.03**	-0.04	-0.01
Date/Day orientation (ref: no correct answer)			
1 correct answer	0.02	-0.04	0.08
2 correct answers	0.04	-0.01	0.09
3 correct answers	0.05	-0.001	0.09
All correct answers	**0.05**	0.004	0.10
Memory test	**0.004**	0.003	0.01
**Random Part**			
Level 2 (Individual)			
Intercept variance	0.02		
Age centered (linear term) variance	0.00003		
Covariance of intercept and age centered	-0.0003		
Level 1 (wave)			
Intercept	0.02		
Number of observations (level 1)	5547		
Number of clusters (level 2)	21573		
-2*Log Likelihood	-11874.03		

Boldface indicates statistical significance (p<0.05)

For peer review only

For peer review only - http://bmjopen.bmj.com/site/about/guidelines.xhtml

STROBE 2007 (v4) Statement—Checklist of items that should be included in reports of *cohort studies*

Section/Topic	Item #	Recommendation	Reported on page #
**Title and abstract**	1	(*a*) Indicate the study's design with a commonly used term in the title or the abstract	2
		(*b*) Provide in the abstract an informative and balanced summary of what was done and what was found	2
**Introduction**			
Background/rationale	2	Explain the scientific background and rationale for the investigation being reported	4-5
Objectives	3	State specific objectives, including any prespecified hypotheses	5
**Methods**			
Study design	4	Present key elements of study design early in the paper	6
Setting	5	Describe the setting, locations, and relevant dates, including periods of recruitment, exposure, follow-up, and data collection	6
Participants	6	(*a*) Give the eligibility criteria, and the sources and methods of selection of participants. Describe methods of follow-up	6-9
		(*b*) For matched studies, give matching criteria and number of exposed and unexposed	NA
Variables	7	Clearly define all outcomes, exposures, predictors, potential confounders, and effect modifiers. Give diagnostic criteria, if applicable	6-7
Data sources/ measurement	8*	For each variable of interest, give sources of data and details of methods of assessment (measurement). Describe comparability of assessment methods if there is more than one group	6-7
Bias	9	Describe any efforts to address potential sources of bias	8-9
Study size	10	Explain how the study size was arrived at	8
Quantitative variables	11	Explain how quantitative variables were handled in the analyses. If applicable, describe which groupings were chosen and why	6-7
Statistical methods	12	(*a*) Describe all statistical methods, including those used to control for confounding	8-9
		(*b*) Describe any methods used to examine subgroups and interactions	8
		(*c*) Explain how missing data were addressed	8-9
		(*d*) If applicable, explain how loss to follow-up was addressed	8-9
		(*e*) Describe any sensitivity analyses	NA
**Results**			

For peer review only

Participants	13*	(a) Report numbers of individuals at each stage of study—eg numbers potentially eligible, examined for eligibility, confirmed eligible, included in the study, completing follow-up, and analysed	11
		(b) Give reasons for non-participation at each stage	8-9
		(c) Consider use of a flow diagram	NA
Descriptive data	14*	(a) Give characteristics of study participants (eg demographic, clinical, social) and information on exposures and potential confounders	9-10
		(b) Indicate number of participants with missing data for each variable of interest	9-10
		(c) Summarise follow-up time (eg, average and total amount)	NA
Outcome data	15*	Report numbers of outcome events or summary measures over time	NA
Main results	16	(*a*) Give unadjusted estimates and, if applicable, confounder-adjusted estimates and their precision (eg, 95% confidence interval). Make clear which confounders were adjusted for and why they were included	12
		(*b*) Report category boundaries when continuous variables were categorized	12
		(*c*) If relevant, consider translating estimates of relative risk into absolute risk for a meaningful time period	12
Other analyses	17	Report other analyses done—eg analyses of subgroups and interactions, and sensitivity analyses	Supplementary materials
**Discussion**			
Key results	18	Summarise key results with reference to study objectives	14
**Limitations**			
Interpretation	20	Give a cautious overall interpretation of results considering objectives, limitations, multiplicity of analyses, results from similar studies, and other relevant evidence	14-16
Generalisability	21	Discuss the generalisability (external validity) of the study results	15
**Other information**			
Funding	22	Give the source of funding and the role of the funders for the present study and, if applicable, for the original study on which the present article is based	17

*Give information separately for cases and controls in case-control studies and, if applicable, for exposed and unexposed groups in cohort and cross-sectional studies.

Note: An Explanation and Elaboration article discusses each checklist item and gives methodological background and published examples of transparent reporting. The STROBE checklist is best used in conjunction with this article (freely available on the Web sites of PLoS Medicine at http://www.plosmedicine.org/, Annals of Internal Medicine at http://www.annals.org/, and Epidemiology at http://www.epidem.com/). Information on the STROBE Initiative is available at www.strobe-statement.org.

For peer review only
